# Blue-Winged Teals in Guatemala and Their Potential Role in the Ecology of H14 Subtype Influenza a Viruses

**DOI:** 10.3390/v15020483

**Published:** 2023-02-09

**Authors:** Lucia Ortiz, Ginger Geiger, Lucas Ferreri, David Moran, Dione Mendez, Ana Silvia Gonzalez-Reiche, Danilo Alvarez, Mayra Motta, Francisco Escobar, Daniela Rajao, Celia Cordon-Rosales, Martha I. Nelson, Daniel R. Perez

**Affiliations:** 1 Poultry Diagnostic and Research Center, Department of Population Health, College of Veterinary Medicine, University of Georgia, Athens, GA 30602, USA; 2 Centro de Estudios en Salud, Universidad del Valle de Guatemala, Guatemala City 01015, Guatemala; 3 Department of Microbiology and Immunology, Emory University School of Medicine, Atlanta, GA 30322, USA; 4 Department of Genetics and Genomic Sciences, Icahn School of Medicine at Mount Sinai, New York, NY 10029, USA; 5 Laboratorio de Referencia Regional de Salud Animal, Facultad de Medicina Veterinaria y Zootecnia, Universidad del San Carlos de Guatemala, Guatemala City 01012, Guatemala; 6 Computational Biology Branch, National Center for Biotechnology Information, National Library of Medicine, National Institutes of Health, Bethesda, MD 20894, USA

**Keywords:** avian influenza, blue-winged teals, H14 subtype, Guatemala, surveillance

## Abstract

Wild aquatic birds are considered the natural hosts of 16 HA (H1–H16) and 9 NA (N1–N9) subtypes of influenza A viruses (FLUAV) found in different combinations. H14 FLUAVs are rarely detected in nature. Since 2011, H14 FLUAVs have been consistently detected in Guatemala, leading to the largest collection of this subtype from a single country. All H14 FLUAVs in Guatemala were detected from blue-winged teal samples. In this report, 17 new full-length H14 FLUAV genome sequences detected from 2014 until 2019 were analyzed and compared to all published H14 sequences, including Guatemala, North America, and Eurasia. The H14 FLUAVs identified in Guatemala were mostly associated with the N3 subtype (*n* = 25), whereas the rest were paired with either N4 (*n* = 7), N5 (*n* = 4), N6 (*n* = 1), and two mixed infections (N3/N5 *n* = 2, and N2/N3 *n* = 1). H14 FLUAVs in Guatemala belong to a distinct H14 lineage in the Americas that is evolving independently from the Eurasian H14 lineage. Of note, the ORF of the H14 HA segments showed three distinct motifs at the cleavage site, two of these containing arginine instead of lysine in the first and fourth positions, not previously described in other countries. The effects of these mutations on virus replication, virulence, and/or transmission remain unknown and warrant further studies.

## 1. Introduction

Influenza A viruses (FLUAV) infect a wide range of bird species and mammals, including humans [1]. The virus genome contains 8 segments of negative single-stranded RNA encoding 6 internal (PB2, PB1, PA, NP, M, and NS) and 2 surface (HA and NA) gene segments. Subtype classification is based on the antigenic properties of the surface glycoproteins. To date, 18 HA (H1–H18) and 11 NA (N1–N11) subtypes have been described [2,3,4]. Wild aquatic birds in the orders Anseriformes and Charadriiformes are considered the natural hosts for 16 HA (H1–H16) and 9 NA (N1–N9) subtypes, playing an important role in the perpetuation of FLUAVs in nature. H3, H4, and H6 subtypes are usually detected at high frequencies in the breeding grounds of North America, while others such as H8, H12, H14, and H15 are rarely found [5,6].

The H14 subtype was initially detected in 1982 in 4 virus isolates obtained from 3 mallard ducks and 1 herring gull in the former Soviet Union (Russia and Kazakhstan) [7]. After no further detections of H14 for almost three decades, H14 viruses surprisingly appeared in sea ducks in Wisconsin, USA, in 2010 [8], whereas serological studies show no evidence of H14 subtype FLUAVs in North American ducks prior to 2010 [9]. Four H14 sequences from Eurasia were subsequently made available in the public databases Bacterial and Viral Bioinformatics Resource Center (BV-BRC) and Global Initiative on Sharing Avian Influenza Data (GISAID), accessed on 8 July 2022, including samples from 1 garganey duck in Ukraine in 2006, 1 goose in Pakistan in 2014, and 1 sandpiper and 1 common teal in Russia in 2019. In North America, 16 additional H14 FLUAVs were detected sporadically in the period 2010–2018 from different species of dabbling ducks such as blue-winged-teal, northern-shoveler, long-tailed-duck, mallards, and scoter within the Pacific, Central, and Mississippi flyways [8,10,11,12].

Central America provides a wintering ground for over a hundred species of aquatic birds within the four major flyways of North America (Pacific, Central, Mississippi, and Atlantic). We have previously provided compelling evidence of the diversity of FLUAV subtypes detected in Guatemala in overwintering ducks, particularly blue-wing teals (BWT, *Anas discors*). These previous studies have highlighted the evolution of diverse FLUAV lineages in Guatemala, including divergent variants rarely detected in the United States [13]. H14 subtype FLUAVs were first detected in 2011 from hunter-harvested BWTs and quickly became the most prominent subtype detected in areas of the Southern Pacific coast of Guatemala. Since then, Guatemala has reported the highest number of H14 subtype FLUAVs from a single location [13,14,15,16], from a single species (BWT), raising questions about H14 ecology and the role of Guatemala in the persistence of this otherwise low-frequency subtype. Using whole-genome sequence data from 17 new H14 FLUAVs from Guatemala that were identified from 2014 to 2019, we demonstrate that the H14 FLUAVs in Guatemala are not monophyletic and belong to a distinct H14 lineage in the Americas that is evolving independently from the Eurasian H14 lineage, including in the HA cleavage site. How H14 viruses persist in the Eastern hemisphere remains unknown, but it appears that Guatemala has an outsized role in the subtype’s persistence in the Americas.

## 2. Materials and Methods

### 2.1. Sample Collection

Tracheal and cloacal swabs were collected from hunter-harvested birds during the winter migration seasons (November–March) 2014–2015, 2015–2016, 2016–2017, 2017–2018, and 2018–2019. Samples included the following species of migratory and non-migratory species: *Anas acuta* (*n* = 1), *Anas americana* (*n* = 2), *Anas clypeata* (*n* = 37), *Anas crecca* (*n* = 3), *Anas discors* (*n* = 2208), *Aythya americana* (*n* = 3), *Aythya* sp. (*n* = 4), *Dendrocygna autumnalis* (*n* = 1), *Dendrocygna bicolor* (*n* = 6), *Egretta thula* (*n* = 1), *Fulica americana* (*n* = 2), *Mycteria americana* (*n* = 2), *Numenius americanus* (*n* = 1), *Nycticorax nyctocorax* (*n* = 1), *Patagioenas flavirostris* (*n* = 3), *Phalacrocorax brasilianus* (*n* = 13), *Platalea ajaja* (*n* = 2), *Zenaida asiatica* (*n* = 4). After sampling, tracheal and cloacal swabs from the same bird were pooled in 1 mL of virus transport medium (VTM, Medium 199 with Hanks balanced salt solution, 2 mM l-glutamine, 0.5% bovine serum albumin, 0.35 g/L sodium bicarbonate, 2 × 106 IU/L penicillin, 200 mg/L streptomycin, 2 × 106 IU/L polymyxin B, 250 mg/L gentamycin, 0.5 × 106 IU/L nystatin, 60 mg/L ofloxacin, and 0.2 g/L sulfamethoxazole) as previously described [15] and transported to the laboratory on ice and stored at −70 °C until used. Permits for sampling bird species at sampling sites were obtained from the Center for Conservation Studies (CECON) and the National Council of Protected Areas (CONAP).

### 2.2. Viral RNA Extraction

Viral RNA from combined tracheal and cloacal swabs was extracted from 250 µL of supernatant with Trizol LS reagent (Invitrogen, Carlsbad, CA, USA) using an organic extraction protocol described previously [16,17]. Extracted RNA was resuspended in 100 µL of DEPC-treated water. All extracted RNA was stored at −70 °C until use.

### 2.3. FLUAV Detection by RRT-PCR

Swabs were screened in duplicate for FLUAV using real-time reverse-transcriptase polymerase chain reaction (RRT-PCR) targeting the matrix gene, as described previously [13,18,19]. Briefly, a QuantiTect Probe RT-PCR (reverse transcription polymerase chain reaction) Kit (QIAGEN, Hilden, Germany) was used to perform RRT-PCR reactions in the ABI 7300 Real-Time PCR System (Applied Biosystems, Foster City, CA, USA). Each reaction contained 12.5 µL of kit-supplied 2X RT-PCR Master mix, 10 pmol of each primer, 0.3 μM probe, 0.25 µL of kit-supplied enzyme mix, 6.5 U RNase inhibitor, and 8 µL of RNA template, as described previously [13,15,16]. Thermal cycling conditions were as follows: one cycle of reverse transcription at 50 °C for 30 min and 94 °C for 15 min, followed by 45 cycles of denaturation at 94 °C for 1 s and combined annealing and extension at 60 °C for 27 s. Fluorescence signals were obtained at the end of each cycle after the annealing/extension step. Swabs showing Ct values < 40 were considered positives.

### 2.4. Multi-Segment RT-Amplification of FLUAV Gene Segments (MS-RTPCR)

MS-RTPCR was performed in all FLUAV-positive swabs, as described previously [20] with minor modifications. Briefly, 2.5 µL of extracted RNA was used as a template in a 25 µL MS-RTPCR reaction (Superscript III high-fidelity RT-PCR kit, ThermoFisher, Waltham, MA, USA), using Opti1-F1 (0.06 µM), Opti1-F2 (0.14 µM), and Opti1-R1 (0.2 µM) primers [14]. The cycling conditions were: 55 °C for 2 min, 42 °C for 1 h, 5 cycles (94 °C for 30 s, 44 °C for 30 s, 68 °C for 3 min), followed by 31 cycles (94 °C for 30 s, 57 °C for 30 s, 68 °C for 3 min) with a final extension at 68 °C for 10 min. The MS-RTPCR final product was analyzed in 1% agarose gel to corroborate whole genome amplification.

### 2.5. Virus Isolation

Influenza virus isolation was attempted in triplicate from samples that tested positive using RRT-PCR FLUAV but failed or yielded incomplete genomes after direct NGS sequencing. Each positive bird sample was tested in triplicate. For each bird sample, a pool of the tracheal/cloacal swab sample (200 µL) was inoculated into the allantoic cavity 9-day-old specific-pathogen-free (SPF) embryonated chicken eggs following the protocol described in the World Health Organization’s Manual on Animal Influenza Diagnosis and Surveillance [21]. Collected allantoic fluids were tested using hemagglutination assay to assess for the presence of FLUAV. Allantoic fluids that tested negative were diluted 1:2 with sterile phosphate-buffered saline (pH 7.4) with 1X antibiotic and antimycotic solution (Corning, Corning, NY, USA) and inoculated in a new batch of three SPF eggs. The process was repeated three times as needed. Samples were considered negative for the presence of viable virus if no FLUAV growth was detected after three serial passages. Positive allantoic fluid was aliquoted and stored at −70 °C until later use.

### 2.6. Sequencing and Genome Assembly

MS-RTPCR products were sequenced using the Illumina platform as described previously [14] with minor modifications. Briefly, amplicons from MS-RTPCR reactions were cleaned using 0.45X of Agencourt AMPure XP Magnetic Beads (Beckman Coulter, Brea, CA, USA), according to the manufacturer’s protocol, and eluted in 30 µL of HyClone molecular biology water (Genesee Scientific, San Diego, CA, USA). Amplicons were quantified using a Qubit buffer kit (ThermoFisher, Waltham, MA, USA) in a Qubit 3.0 fluorometer (ThermoFisher, Waltham, MA, USA) and normalized to 0.2 ng/µL. Adaptors were added via tagmentation using the Nextera XT DNA library preparation kit (Illumina, San Diego, CA, USA). The reaction was set as 60% of the suggested final volume. Samples were purified using 0.7X of Agencourt AMPure XP Magnetic Beads and analyzed on a Bioanalyzer using a High Sensitivity DNA kit (Agilent, Santa Clara, CA, USA) to determine the distribution of fragment size. Libraries were pooled and normalized to 1–4 nM. After denaturation, the final loading concentration of the pooled libraries was 14 pM. Libraries were sequenced using a MiSeq Reagent Kit V2, 300 cycles (Illumina, San Diego, CA, USA). Genome assembly was performed using a customized pipeline developed at the Icahn School of Medicine at Mount Sinai [22].

### 2.7. Phylogenetic Analyses

Independent phylogenetic analyses were performed for the surface (HA and NA) and internal (PB2, PB1, PA, NS, NP, and M) gene segments. Sequences were aligned with MUSCLE 5.1 [23] and manually trimmed to keep the open reading frame (ORF) of the gene of interest. Background sequences were subsampled to remove identical sequences and those sequences with less than 80% of the total length of the ORF using a SeqKit 0.16.1 bioinformatic tool [24]. The best-fit model of nucleotide substitution was determined for each gene using the Bayesian information criterion (BIC) obtained using jModelTest 2.1.10 [25]. For N3, N4, and N5, as well as the internal gene segments, phylogenetic trees were constructed using maximum-likelihood (ML) inference methods, using RAxML 8.2.12 [26] under the general time reversible GTR + G nucleotide substitution model with 1000 bootstrap replicates. Trees were run at least twice to confirm topology. H14 phylogeny was inferred via time-scaled phylogenetic analysis using the Bayesian Markov chain Monte Carlo (MCMC) approach as implemented in BEAST 1.10.4 [27,28]. A relaxed uncorrelated lognormal (UCLN) molecular clock was used, with a constant population size, and a general-time reversible (GTR + G) model of nucleotide substitution with gamma-distributed rate variation among sites. Three independent analyses of 50 million generations were performed to ensure convergence, sampling every 5000 states. The burn-in percentage of each dataset was identified using Tracer v1.7.1 [29] and after its removal, results were combined using LogCombiner v1.10.4 [27,28]. The MCC trees were annotated using TreeAnnotator v1.10.4 [27,28]. Outlier sequences were identified using TemEst V1.5.3 [30]. When necessary, outlier sequences were removed, and the datasets were run again as described above. The resulting trees were visualized in FigTree 1.4.4 (http://tree.bio.ed.ac.uk/software/Figtree/) and aesthetically modified using Inkscape v0.48.1 (https://inkscape.org). H14 virus phylodynamic analysis was done in BEAST 1.10.4 [27,28] considering a discrete space of three locations (Eurasia, North America, and Guatemala), as well as discrete traits (HA cleavage site and NA), with an asymmetric substitution model. Significant transition rates were identified using Bayes factors (BF): ≥1000 was deemed as decisive support, 100 ≤ BF < 1000 as very strong support, 10 ≤ BF < 100 as strong support, and 3 ≤ BF < 10 as supported. The resulting MCC tree was visualized on SpreaD3 [31] and aesthetically modified using Inkscape v0.48.1 (https://inkscape.org). Gene lineages and genotype assignments were performed based on the phylogenetic trees as described previously [32]. Briefly, FLUAV genes with ≥95% nucleotide identity supported by bootstrap values >70% were classified in the same phylogenetic clade, whereas with ≥90% nucleotide identity within the same lineage.

### 2.8. Pairwise Percentage of Identity

Pairwise percentage of identity was calculated in Geneious Prime 2021.2.2 (https://www.geneious.com), and resulting values were plotted in GraphPad Prism v.9.1.0 (www.graphpad.com).

### 2.9. Amino Acid Sequence Analysis

Nucleotide sequences were translated into amino acids using Geneious Prime 2021.2.2 (https://www.geneious.com). The resulting amino acid sequences were used to identify virulence markers and specific motifs in the sequences.

## 3. Results

### 3.1. Genetic and Phylogenetic Characterization of H14 FLUAVs from Guatemala

During five wintering seasons (November–March) of FLUAV surveillance in avian species on the Pacific Coast of Guatemala (seasons 2014–2015, 2015–2016, 2016–2017, 2017–2018, and 2018–2019), 2294 paired tracheal/cloacal swab samples were obtained from hunter-harvested wild aquatic birds. From those samples, 96.5% corresponded to BWT. Swab samples that tested positive (*n* = 234) for FLUAV’s matrix (M) gene using RRT-PCR screening were used for MS-RT-PCR followed by NGS sequencing. Whole virus genome sequences were obtained from 89 out of 234 FLUAV-positive samples (38.2%) either from the swab material (*n* = 45) or virus isolate (*n* = 44), following the approach described by Ferreri et al. [14]. The HA and NA gene segments were successfully sequenced from an additional 12 swab samples that produced incomplete internal gene segment sequences. In addition, 15 additional swab samples produced at least one gene segment sequence; overall, 116 samples produced at least one complete gene segment.

**Figure 1:**
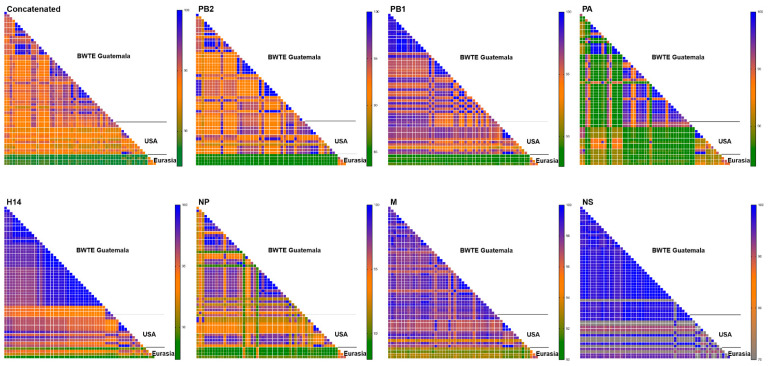
Pairwise percentage identity of major ORF sequences of H14 full-length genomes isolated from Guatemala (*n* = 40), North America (USA, *n* = 12), and Eurasia (*n* = 4) during 1982–2019. The gradient background color represents the increase in mean pairwise nucleotide distances, going from the smallest distance in dark green, to the largest, in dark blue.

In this report, we focus the attention on the 17 H14 subtype strains identified (7 from swab material and 10 from viral isolate), all of which were found exclusively in BWT samples. The proportion of other identified subtypes can be found in Figure S1. In combination with previously reported H14 sequences for the 2011–2013 period, this new set of sequences brings the total number of Guatemalan H14 strains to *n* = 40 full H14 HA gene segment sequences (all of them with complete virus genome sequences). Subtype combinations of all H14 from Guatemala included H14N3 (*n* = 25), H14N4 (*n* = 7), H14N5 (*n* = 4), H14N6 (*n* = 1), and three mixed infections (2 H14N3/N5 and 1 H14N2/N3). To determine the genetic similarity among the Guatemalan H14 FLUAVs, mean pairwise percentage nucleotide identities were established using either concatenated or the major ORF sequences of each gene segment. Genetic comparisons across the concatenated gene segments (PB2, PB1, PA, HA, NP, M, and NS) from the 40 full genome sequences revealed 95.4% mean pairwise nucleotide identity (Figure 1).

At the individual gene level, the M1 ORF sequences presented the highest % mean pairwise identity (98.0%), followed by HA 97.7%, PB1 96.9%, NS1 96.6%, and NP 95.2%. The PA of H14 viruses from Guatemala belongs to the two North American lineages with 95.6% (*n* = 12) and 96.8% (*n* = 28) pairwise identity within each lineage. Identical H14 HA sequences were detected within the Guatemalan BWT samples (28 out 40), most of them identified from samples collected the same day. Detailed % mean pairwise identity can be found in Table S1–S11.

The 40 full-genome H14 virus sequences from Guatemala were compared to the 16 H14 full-length virus genomes from North America (*n* = 12, from Arkansas, Ohio, Texas, Wisconsin, California, Mississippi, and Missouri) and Eurasia (*n* = 4, from Russia, Ukraine, and Pakistan) available on BV-BRC (Bacterial and Viral Bioinformatics Resource Center, https://www.bv-brc.org/, accessed on 1 July 2022) and GISAID (https://gisaid.org/, accessed on 8 July 2022) databases. The similarity of the Guatemalan and North American H14 FLUAVs is less than 95% based on concatenated internal gene segment sequences, suggesting active virus reassortment in Guatemala. Similar analyses revealed mean pairwise identity of 88.5% compared to H14 Eurasian sequences (Figure 1). Analyses using a single gene segment sequence showed mean pairwise percent identity between Guatemalan and North American sequences as follows: PB2 93.1%, PB1 95.9%, H14 HA 95.8%, NP 94.1%, and M1 97.2%. PA for the two circulating North American lineages showed 95.5% and 92.0% within each lineage. Similar analyses revealed lower identity of Guatemalan viruses with Eurasian lineage viruses: PB2 84.6%, PB1 88.7%, PA 88.8%, H14 HA 90.5%, NP 89.5%, and M1 92.7%. The Guatemalan H14 samples had both NS alleles: 39 strains carried allele A, while 1 H14N4 virus (sample collected in 2015) carried allele B. Mean pairwise NS segment identification revealed closer relationship of both alleles (Allele A and Allele B) to North American sequences (95.8% for Allele A and 97.5% for Allele B) compared to Eurasian sequences (94.1% for Allele A and 91.2% for Allele B).

### 3.2. H14 FLUAVs Were Introduced in Guatemala at Least Twice

**Figure 2:**
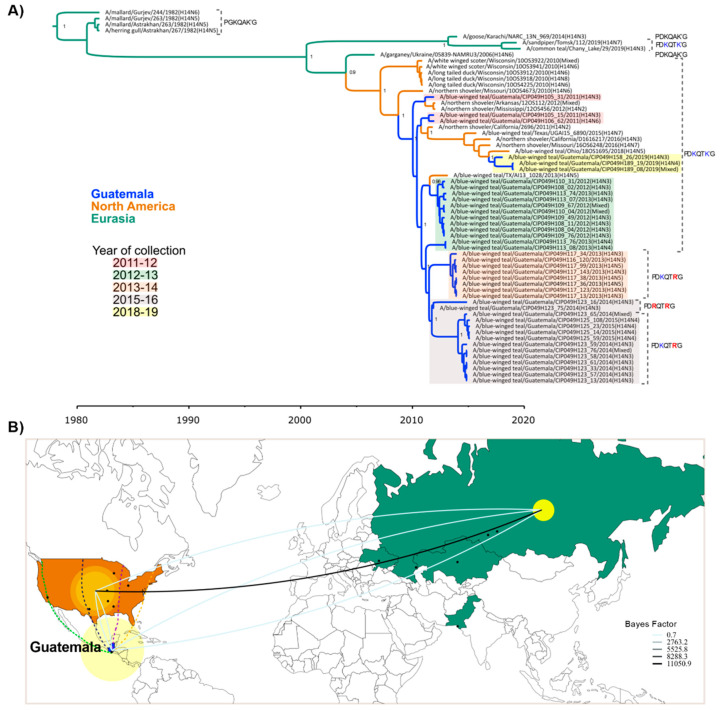
Spatiotemporal diffusion of H14 viruses reported worldwide (*n* = 62) during 1982–2019. Geographic location of H14 viruses from Guatemala (blue), North America (orange), and Eurasia (green) are shown. (**A**) Time-scaled maximum clade credibility (MCC) tree inferred for the hemagglutinin gene (HA). Branches are colored according to geographic location. Collection season of Guatemalan H14 is indicated by color. HA cleavage motif is shown for each isolate. Alternative amino acids in the HA cleavage motifs from Guatemalan H14 are shown in red, while the amino acid HA cleavage motif found in North America and Eurasia is shown in blue. Posterior probabilities > 0.9 are included for key nodes. (**B**) Spatial diffusion of H14 viruses. Yellow circles represent discrete geographical locations of H14 viruses and lines represent branches in the MCC tree along with where the relevant location transition occurs. The diameter of circles represents the number of lineages with that location. H14 viruses’ location sites were coded as discrete geographical locations: Guatemala (*n* = 40), North America (Arkansas = 1, California = 2, Mississippi = 1, Missouri = 2, Ohio = 1, Texas = 2, and Wisconsin = 5), and Eurasia (Russia = 4, Ukraine = 1, Kazakhstan = 2, Pakistan = 1). Location where H14 viruses have been identified were plotted as a centroid of a state or city to enable its visualization. Main North American migratory flyways (Pacific flyway in green, Central flyway in dark blue, Mississippi flyway in purple, and Atlantic flyway in yellow) are depicted from the US through Guatemala. Representation of the discrete geographic location transitions of H14 viruses are included showing the highest Bayes factor supported transition rates among these locations. Westward and eastward movements are depicted by upward and downward curvature lines, respectively.

Phylogenetic analyses were performed with all H14 HA sequences available to date (Figure 2A). The phylogenetic tree showed segregation of H14 FLUAVs into Eurasian and North American clades [11,13]. The Guatemalan H14 FLUAVs clustered together with viruses from North America with similar dates of collection (Figure 2A). These analyses further revealed one major introduction in 2011 [13] and then a second introduction in 2019. The closest ancestors of the HA of the H14 Guatemalan viruses were identified in the Mississippi flyway as previously observed [11,33]. After its first detection in 2011, the H14 viruses persisted in Guatemala until ~2014.6 to 2014.9 (95% highest posterior density (HPD)). The most recent H14 virus identified in Guatemala (samples collected in 2019) clustered in a separate clade with other North American viruses, indicating a recent independent introduction of the H14 subtype in the country. Phylodynamic analysis considering a discrete space of three locations (Guatemala, North America, and Eurasia) with an asymmetric substitution model supports the Eurasian movement of H14 strains into the Americas (Bayes factor, BF = 11,050.9); however, due to the limited number of H14 sequences, it is not possible to establish significant movements between North America and Guatemala (Figure 2B and Table S12).

**Figure 3:**
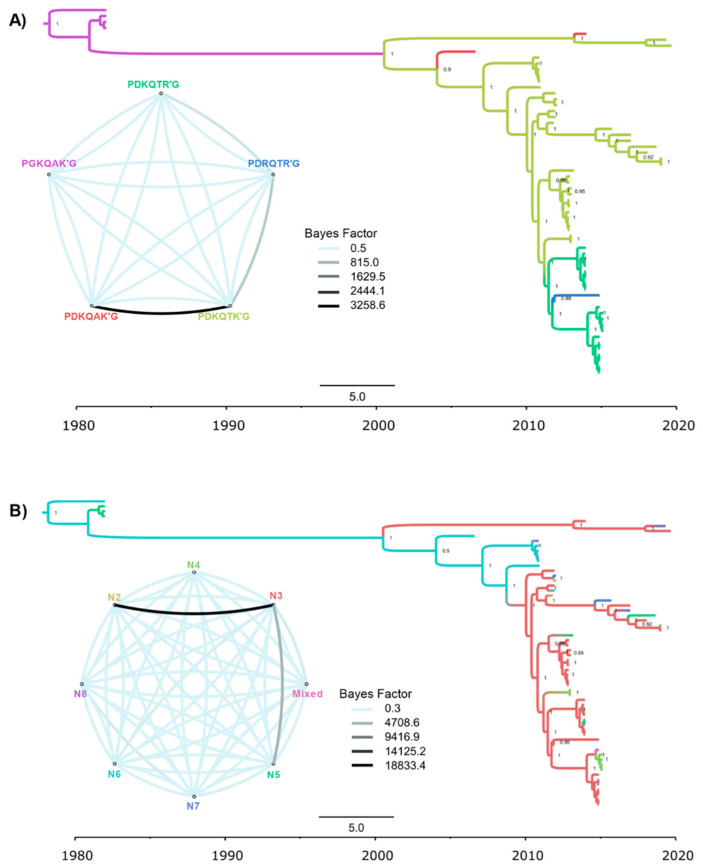
Time-scaled maximum clade credibility (MCC) tree inferred for the Hemagglutinin gene (HA) of global H14 viruses (*n* = 62) during 1982–2019, showing the highest Bayes factor supported transition rates among these traits. Posterior probabilities > 0.9 are shown. Branches are colored according to 2 discrete traits: (**A**) HA cleavage site motif. (**B**) Neuraminidase subtype.

It is well accepted that most FLUAVs rely on the host’s trypsin-like proteases for cleavage of the HA into subunits, HA1 and HA2, to render the virus infectious. Among the H14 Guatemalan HA ORF sequences, three different predicted motifs at the HA cleavage site were identified. Sequences from samples collected in seasons 2010–2011 and 2012–2013 and samples (*n* = 3) collected in season 2018–2019 showed the motif PDKQTK’GLF, typical of H14 HA sequences previously identified in North American bird samples [10,11] and two other samples from Eurasia (Figure 2A and Figure 3A). The cleavage site of these H14 HA sequences are unusual compared to other HA subtypes since they contained the amino acid lysine (K) instead of arginine (R) at the −4 and −1 positions on the HA1/HA2 cleavage site [7]. However, we have also detected additional alternative motifs: H14 HAs collected from late 2013 to mid-2016 (seasons 2013–2014, 2014–2015, and 2015–2016) contained either the motif PDKQT**R**’G (R, at the −1 position, *n* = 12 sequences) or PD**R**QT**R**’G (R, at the −1 and −4 positions, *n* = 2 sequences), the latter more typical of other HA subtypes.

A discrete traits analysis (Figure 3A and Table S13) inferred that a PGKQAKG-to-PDKQTKG transition in the HA cleavage site occurred in Eurasia between the 1980s and early 2000s. The PDKQTKG motif persisted in Eurasian birds, where it was detected as recently as 2019. Two independent PDKQTKG-to-PDKQAKG transitions also occurred in Eurasia, but no PDKQAKG motifs have been observed since 2014. The first H14 viruses identified in North America retained the Eurasian PDKQTKG motif, which also persisted in North America until as recently as 2019. A PDKQTKG-to-PDKQTRG transition was observed in Guatemala in the early 2010s, followed by a PDKQTRG-to-PDRQTRG transition, although the latter transition was only observed in only two viruses. NA subtypes were swapped more frequently (Figure 3B and Table S14). Decisive Bayes factor support (BF > 1000) was observed for the transition from N3 to N2 (BF= 18,833.4) and N3 to N5 (BF= 4703.6). There was no apparent association between the HA cleavage site motif and NA subtype.

### 3.3. Diverse Gene Constellations Detected in Blue-Winged Teals in Guatemala

**Figure 4:**
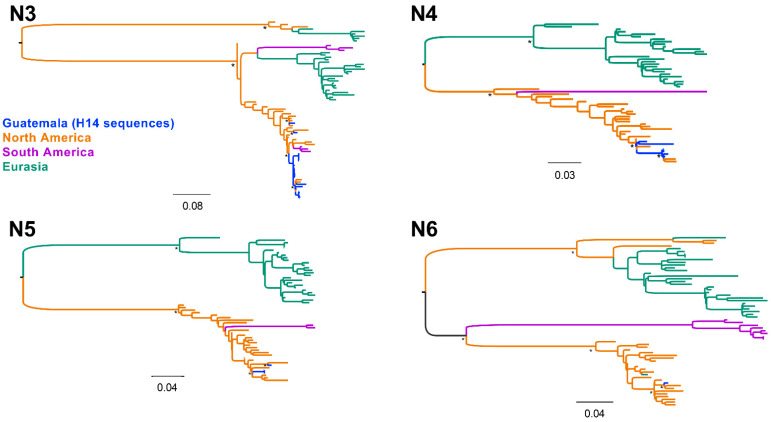
Neuraminidase gene (NA) phylogenetic inference for all H14 virus coding sequences from Guatemala (2011–2019) and other randomly sampled viruses from North America, South America, and Eurasia. Maximum likelihood phylogenetic inference using the best-fit model. H14 viruses from Guatemala are indicated in blue. Identical H14 viral sequences were removed. The tree is midpoint rooted, and all branch lengths are drawn to scale. Viruses are shaded according to geographic location: North America (orange), Eurasia (green), South America (purple). Bootstrap values > 70% for key nodes are indicated by asterisks (*).

**Figure 5:**
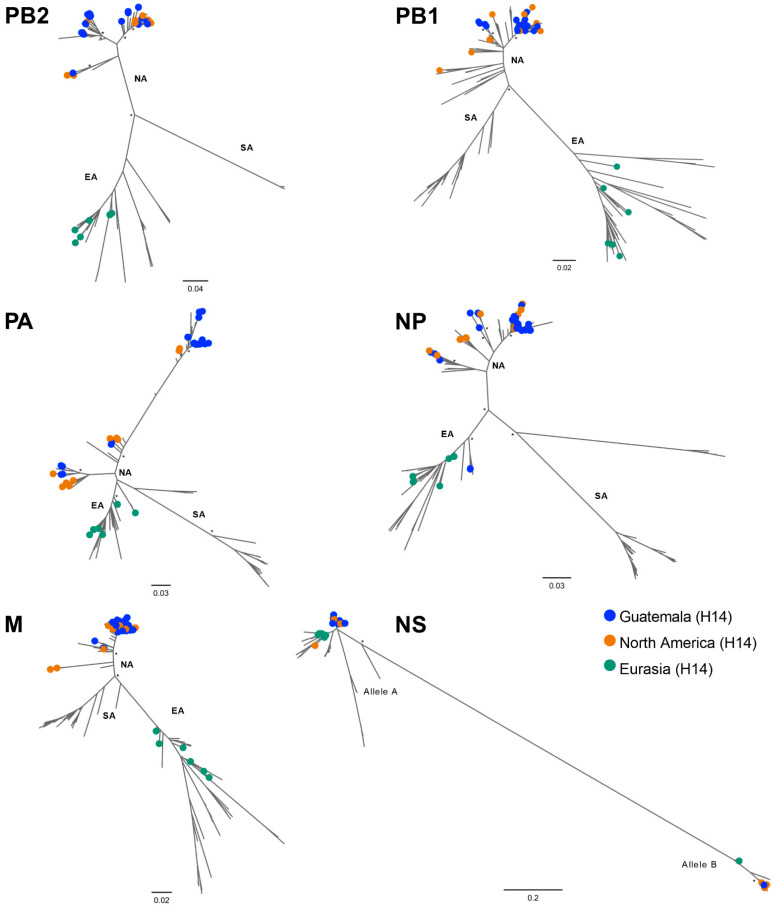
Internal genes (PB2, PB1, PA, NP, M, and NS) phylogenetic inference for all H14 virus coding sequences available worldwide. Maximum likelihood phylogenetic inference using the best-fit model. Identical H14 viral sequences were removed. Geographic region of H14 viruses is indicated by colored circles. Scale bar on the bottom-left indicates the number of nucleotide substitutions per site. The different lineages from North America (NA), Eurasia (EA), and South America (SA) are shown. Scale bar on the bottom-left indicates the number of nucleotide substitutions per site. Bootstrap values > 70% for key nodes are indicated by asterisks (*) for key nodes.

All NAs and internal gene segments of the H14 FLUAVs from Guatemala grouped within the North American lineage (Figure 4 and Figure S2–S5). Mean pairwise comparisons among the ORFs of distinct NA subtypes showed 97.9% sequence identity within the N3 (*n* = 27), 97.3% (*n* = 7) within N4, and 97.4% (*n* = 4) within N5. These analyses also revealed high nucleotide identity (>95%) with contemporary sequences of viruses circulating within the Pacific and Mississippi flyways, consistent with previous observations [13,14,15]. Of note, the NP gene of two H14N3 viruses collected in 2013 are recent introductions from the Eurasian lineage along with those from some contemporary North American viruses isolated from multiple flyways (Figure 5) and >98% similar to a H3N8 virus from Alaska isolated in 2009 (Table S15) whose introduction from the Eurasian lineage has been previously documented [34].

**Figure 6:**
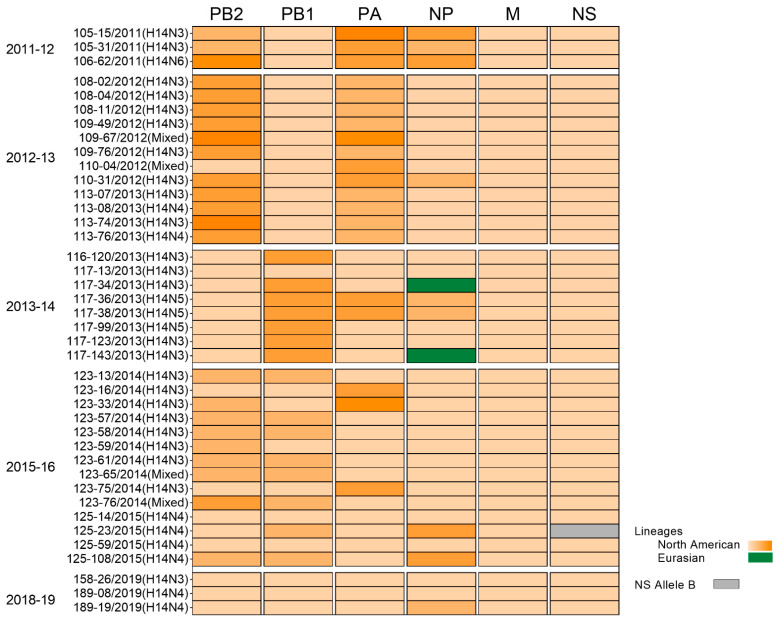
Internal gene (PB2, PB1, PA, NP, M, and NS) constellations of H14 viruses from Guatemala (*n* = 40) during 2011–2019. Orange and green represent the North American and Eurasian lineages respectively. Grey corresponds to the Allele B of the NS segment. Different clades of the North American linage are represented by gradient orange color depending on the number of phylogenetic clades with bootstrap support values of >70% of each segment (PB2 = 5, PB1 = 3, PA = 4, NP = 3, M = 1, NS allele A = 1, and NS allele B = 1). Collection season and sample ID are indicated. Sequences from seasons 2015–16 and 2018–19 were sequenced for this study. All samples were collected from blue-winged teals.

Phylogenetic inference via the maximum likelihood method revealed internal genes of H14 sequences grouped in subclades of reference sequences within a particular lineage (Figure 5, Figure 6 and Figure S7–S11). Overall, the 40 H14 Guatemalan FLUAVs were associated with 20 clades with bootstrap support values of >70% of each segment (PB2 = 5, PB1 = 3, PA = 5, NP = 4, M = 1, and NS = 2) (Figure 6). Interestingly, the internal gene segments of samples collected the same day and with identical H14 HA sequences were positioned in different clades on the phylogenetic tree, highlighting the high virus diversity found in these viruses in Guatemala.

Further analyses of the remaining predicted ORFs revealed neither mammalian-associated virulence markers in PB2 (E627K and/or D701N) [35,36] and PA (S409N) [37], nor NS1 protein (T92E) [38] or antiviral resistance markers (V27A and S31N on M2 [39] or H274Y on NA). The PB1-F2 N66 (asparagine) marker associated with increased virulence in mammals [40] was detected in 15 out 40 samples. The samples with the PB1-F2 N66 marker encoded an 87 aa long protein, whereas the rest carrying the PB1-F2 S66 (serine) signature encoded a 90 aa long protein. PB1-F2 proteins with either one of those lengths have been previously described in FLUAVs from ducks [39]. All Guatemalan H14 FLUAVs encoded a full-length PA-X protein of 252 amino acids in length.

## 4. Discussion

During more than a decade of FLUAV surveillance in wild birds in Guatemala, an unusually large number of H14 subtype viruses were detected [13,14,15,16,41]. Prior to its initial isolation in North America in 2010, there was no evidence of significant circulation of this subtype in the Western hemisphere [9]. The H14 subtype FLUAVs appeared predominantly in Guatemala from the first detection in 2011 [13,14,16] through 2019. In this study, we analyzed a small data set of available sequences from H14 viruses worldwide (*n* = 62); however, our current observations are consistent with our initial findings [13]. Due to the limited information about this subtype, its ecology and how it is maintained in this region is unknown. One hypothesis for the high level of H14 detected in Guatemala is that it may be due to epizootic events originated from a single introduction, followed by local clonal expansion of the newly introduced subtype that later was maintained in local populations [42]. Studies of H3 subtype FLUAV epizootic events in mallards suggest that migrant birds play an important role as vectors of novel strains that are introduced and amplified by local bird populations [42]. It is feasible that H14 FLUAVs are maintained in susceptible hosts during non-migration seasons and later transmitted during migration season by a routinely sampled host, such as BWT. H14 viruses from Guatemala were exclusively detected from hunter-harvested BWT. Therefore, our sampling is biased towards game bird species that are in large numbers. BWT is one of the most abundant species of dabbling ducks in North America and has been found in breeding grounds from North America to wintering grounds in Mexico, Central America, and parts of South America [43]. Due to the wide distribution of BWT across the American continent, it is intriguing as to why the H14 subtype has become predominant only in Central America in BWT. We believe that yet to be defined local conditions in Guatemala may have provided the adequate environment for this subtype to thrive. We speculate that such conditions may explain in part the detection of H14 in Guatemala from 2011 to 2015, when all H14 HAs shared the same common ancestor. However, the distinct NA subtype combinations and internal gene segment constellations of Guatemalan H14 viruses within and between migratory seasons indicate active reassortment and independent introductions from unknown sources.

When the first H14 HA subtype was initially sequenced, one feature that stood out was the presence of K instead of R at the −1 position in the HA cleavage site [7,8,10,11,33]. Thus, previously described H14 HA from North America showed the PDKQTK’G motif, which is unusual among FLUAV subtypes. This rarer motif was detected initially during our surveillance, but it was also still present in Guatemala in 2019. Our continuous surveillance and sequencing approach directly from swabs revealed two additional cleavage site motifs in the H14 HAs, PDKQTR’G and PDRQTR’G, which place them closer to the more typical cleavage sites observed in other HA subtypes. It is commonly accepted that the HA cleavage is predicted to be a substrate for trypsin-like proteases found in the lumen of the intestinal tract of natural FLUAV hosts. Cleavage of HA is necessary for virus infection that modulates virulence. At this stage it is unclear how these different cleavage site motifs in the HA affect replication, virulence, and/or transmission of H14 viruses in natural and/or in potential accidental hosts (poultry, mammals) [44]. To our knowledge, it remains to be determined whether H14 FLAUV infections cause any disease in wild birds. Similarly, to our knowledge, there have been no reports of outbreaks of H14 FLUAVs in either poultry or mammals. It is also unclear whether our ability to detect different motifs is related to sequencing directly from swabs as opposed to sequencing from virus isolates obtained through passage in chicken eggs (an artificial substrate with potential bottleneck effects) [14]. Overall, continued detection of H14 FLUAVs in Guatemala appears dictated by potential multiple drivers in the avian reservoir that warrant further studies.

## Data Availability

Nucleotide genomic sequences from all Guatemalan viruses have been deposited at the NCBI Database under the following accession numbers OP144065–OP144203.

## Supplementary Materials

The following supporting information can be downloaded at: https://www.mdpi.com/article/10.3390/v15020483/s1, Figure S1: Number of HA and NA identified in Guatemala; Figure S2: Detailed ML phylogenetic tree for N3; Figure S3: Detailed ML phylogenetic tree for N4; Figure S4: Detailed ML phylogenetic tree for N5; Figure S5: Detailed ML phylogenetic tree for N6; Figure S6: Detailed ML phylogenetic tree for PB2; Figure S7: Detailed ML phylogenetic tree for PB1; Figure S8: Detailed ML phylogenetic tree for PA; Figure S9: Detailed ML phylogenetic tree for NP; Figure S10: Detailed ML phylogenetic tree for M; Figure S11: Detailed ML phylogenetic tree for NS; Table S1: Detailed nucleotide pairwise identity of ORF sequences of concatenated genes; Table S2: Detailed nucleotide pairwise identity of ORF sequences of PB2; Table S3: Detailed nucleotide pairwise identity of ORF sequences of PB1; Table S4: Detailed nucleotide pairwise identity of ORF sequences of PA; Table S5: Detailed nucleotide pairwise identity of ORF sequences of HA; Table S6: Detailed nucleotide pairwise identity of ORF sequences of NP; Table S7: Detailed nucleotide pairwise identity of ORF sequences of M; Table S8: Detailed nucleotide pairwise identity of ORF sequences of NS; Table S9: Detailed nucleotide pairwise identity of ORF sequences of N3; Table S10: Detailed nucleotide pairwise identity of ORF sequences of N4; Table S11: Detailed nucleotide pairwise identity of ORF sequences of N5; Table S12: Bayes factor and posterior probability values using geographic locations; Table S13: Bayes factor and posterior probability values using HA cleavage site; Table S14: Bayes factor and posterior probability values using NA subtype; Table S15: Best ten BLAST hits of Guatemalan isolates.

## References

[1] Webster R.G., Bean W., Gorman O.T., Chambers T.M., Kawaoka Y. (1992). Evolution and ecology of influenza A viruses. Microbiol. Rev..

[2] Gamblin S.J., Skehel J. (2010). Influenza hemagglutinin and neuraminidase membrane glycoproteins. J. Biol. Chem..

[3] Tong S., Li Y., Rivailler P., Conrardy C., Castillo D.A., Chen L.M., Recuenco S., Ellison J.A., Davis C.T., York I.A. (2012). A distinct lineage of influenza A virus from bats. Proc. Natl. Acad. Sci. USA.

[4] Tong S., Zhu X., Li Y., Shi M., Zhang J., Bourgeois M., Yang H., Chen X., Recuenco S., Gomez J. (2013). New world bats harbor diverse influenza A viruses. PLoS Pathog..

[5] Diskin E.R., Friedman K., Krauss S., Nolting J.M., Poulson R.L., Slemons R.D., Stallknecht D.E., Webster R.G., Bowman A.S. (2020). Subtype diversity of influenza A virus in North American waterfowl: A multidecade study. J. Virol..

[6] Wille M., Latorre-Margalef N., Tolf C., Halpin R., Wentworth D., Fouchier R.A.M., Raghwani J., Pybus O.G., Olsen B., Waldenström J. (2018). Where do all the subtypes go? Temporal dynamics of H8-H12 influenza A viruses in waterfowl. Virus Evol..

[7] Kawaoka Y., Yamnikova S., Chambers T.M., Lvov D.K., Webster R.G. (1990). Molecular characterization of a new hemagglutinin, subtype H14, of influenza A virus. Virology.

[8] Nolting J., Fries A.C., Slemons R.D., Courtney C., Hines N., Pedersen J. (2012). Recovery of H14 influenza A virus isolates from sea ducks in the Western Hemisphere. PLoS Curr..

[9] Latorre-Margalef N., Ramey A.M., Fojtik A., Stallknecht D.E. (2015). Serologic evidence of influenza A (H14) virus introduction into North America. Emerg. Infect. Dis..

[10] Boyce W.M., Schobel S., Dugan V.G., Halpin R., Lin X., Wentworth D.E., Lindsay L.L., Mertens E., Plancarte M. (2013). Complete Genome Sequence of a Reassortant H14N2 Avian Influenza Virus from California. Genome Announc..

[11] Ramey A.M., Poulson R.L., González-Reiche A.S., Perez D.R., Stallknecht D.E., Brown J.D. (2014). Genomic characterization of H14 subtype Influenza A viruses in new world waterfowl and experimental infectivity in mallards (*Anas platyrhynchos*). PLoS ONE.

[12] Bowman A.S., Nolting J.M., Massengill R., Baker J., Workman J.D., Slemons R.D. (2015). Influenza A virus surveillance in waterfowl in Missouri, USA, 2005–2013. Avian Dis..

[13] Gonzalez-Reiche A.S., Nelson M.I., Angel M., Müller M.L., Ortiz L., Dutta J., van Bakel H., Cordon-Rosales C., Perez D.R. (2017). Evidence of intercontinental spread and uncommon variants of low-pathogenicity avian influenza viruses in ducks overwintering in Guatemala. mSphere.

[14] Ferreri L.M., Ortiz L., Geiger G., Barriga G.P., Poulson R., Gonzalez-Reiche A.S., Crum J.A., Stallknecht D., Moran D., Cordon-Rosales C. (2019). Improved detection of influenza A virus from blue-winged teals by sequencing directly from swab material. Ecol. Evol..

[15] Gonzalez-Reiche A.S., Morales-Betoulle M.E., Alvarez D., Betoulle J.L., Muller M.L., Sosa S.M., Perez D.R. (2012). Influenza a viruses from wild birds in Guatemala belong to the North American lineage. PLoS ONE.

[16] Gonzalez-Reiche A.S., Müller M.L., Ortiz L., Cordón-Rosales C., Perez D.R. (2016). Prevalence and diversity of low pathogenicity avian influenza viruses in wild birds in Guatemala, 2010–2013. Avian Dis..

[17] Spackman E., Suarez D. (2008). Avian influenza virus RNA extraction from tissue and swab material. Methods Mol. Biol..

[18] Slomka M.J., Densham A.L., Coward V.J., Essen S., Brookes S.M., Irvine R.M., Spackman E., Ridgeon J., Gardner R., Hanna A. (2010). Real time reverse transcription (RRT)-polymerase chain reaction (PCR) methods for detection of pandemic (H1N1) 2009 influenza virus and European swine influenza A virus infections in pigs. Influenza Other Respir. Viruses.

[19] Spackman E., Senne D.A., Myers T.J., Bulaga L.L., Garber L.P., Perdue M.L., Lohman K., Daum L.T., Suarez D.L. (2002). Development of a real-time reverse transcriptase PCR assay for type A influenza virus and the avian H5 and H7 hemagglutinin subtypes. J. Clin. Microbiol..

[20] Zhou B., Donnelly M.E., Scholes D.T., St George K., Hatta M., Kawaoka Y., Wentworth D.E. (2009). Single-reaction genomic amplification accelerates sequencing and vaccine production for classical and Swine origin human influenza a viruses. J. Virol..

[21] WHO (2002). WHO Manual on Animal Influenza Diagnosis and Surveillance.

[22] Mena I., Nelson M.I., Quezada-Monroy F., Dutta J., Cortes-Fernández R., Lara-Puente J.H., Castro-Peralta F., Cunha L.F., Trovão N.S., Lozano-Dubernard B. (2016). Origins of the 2009 H1N1 influenza pandemic in swine in Mexico. eLife.

[23] Edgar R.C. (2004). MUSCLE: Multiple sequence alignment with high accuracy and high throughput. Nucleic Acids Res..

[24] Shen W., Le S., Li Y., Hu F. (2016). SeqKit: A cross-platform and ultrafast toolkit for FASTA/Q file manipulation. PLoS ONE.

[25] Posada D. (2008). jModelTest: Phylogenetic model averaging. Mol. Biol. Evol..

[26] Stamatakis A. (2014). RAxML version 8: A tool for phylogenetic analysis and post-analysis of large phylogenies. Bioinformatics.

[27] Drummond A.J., Rambaut A. (2007). BEAST: Bayesian evolutionary analysis by sampling trees. BMC Evol. Biol..

[28] Suchard M.A., Lemey P., Baele G., Ayres D.L., Drummond A.J., Rambaut A. (2018). Bayesian phylogenetic and phylodynamic data integration using BEAST 1.10. Virus Evol..

[29] Rambaut A., Drummond A.J., Xie D., Baele G., Suchard M.A. (2018). Posterior summarization in Bayesian phylogenetics using Tracer 1.7. Syst. Biol..

[30] Rambaut A., Lam T.T., Max Carvalho L., Pybus O.G. (2016). Exploring the temporal structure of heterochronous sequences using TempEst (formerly Path-O-Gen). Virus Evol..

[31] Bielejec F., Baele G., Vrancken B., Suchard M.A., Rambaut A., Lemey P. (2016). SpreaD3: Interactive visualization of spatiotemporal history and trait evolutionary processes. Mol. Biol. Evol..

[32] Huang Y., Robertson G.J., Ojkic D., Whitney H., Lang A.S. (2014). Diverse inter-continental and host lineage reassortant avian influenza A viruses in pelagic seabirds. Infect. Genet. Evol..

[33] Fries A.C., Nolting J.M., Danner A., Webster R.G., Bowman A.S., Krauss S., Slemons R.D. (2013). Evidence for the circulation and inter-hemispheric movement of the H14 subtype influenza A virus. PLoS ONE.

[34] Koehler A.V., Pearce J.M., Flint P.L., Franson J.C., Ip H.S. (2008). Genetic evidence of intercontinental movement of avian influenza in a migratory bird: The northern pintail (*Anas acuta*). Mol. Ecol..

[35] Hatta M., Gao P., Halfmann P., Kawaoka Y. (2001). Molecular basis for high virulence of Hong Kong H5N1 influenza A viruses. Science.

[36] Li Z., Chen H., Jiao P., Deng G., Tian G., Li Y., Hoffmann E., Webster R.G., Matsuoka Y., Yu K. (2005). Molecular basis of replication of duck H5N1 influenza viruses in a mammalian mouse model. J. Virol..

[37] Yamayoshi S., Yamada S., Fukuyama S., Murakami S., Zhao D., Uraki R., Watanabe T., Tomita Y., Macken C., Neumann G. (2014). Virulence-affecting amino acid changes in the PA protein of H7N9 influenza A viruses. J. Virol..

[38] Ayllon J., Garcia-Sastre A. (2015). The NS1 protein: A multitasking virulence factor. Current Topics in Microbiology and Immunology.

[39] Furuse Y., Suzuki A., Oshitani H. (2009). Large-scale sequence analysis of M gene of influenza A viruses from different species: Mechanisms for emergence and spread of amantadine resistance. Antimicrob. Agents Chemother..

[40] Krumbholz A., Philipps A., Oehring H., Schwarzer K., Eitner A., Wutzler P., Zell R. (2011). Current knowledge on PB1-F2 of influenza A viruses. Med. Microbiol. Immunol..

[41] Gonzalez-Reich A.S., Perez D. (2012). Where do avian influenza viruses meet in the Americas?. Avian Dis..

[42] Verhagen J.H., van Dijk J.G.B., Vuong O., Bestebroer T., Lexmond P., Klaassen M., Fouchier R.A.M. (2014). Migratory birds reinforce local circulation of avian influenza viruses. PLoS ONE.

[43] Rohwer F.C., Johnson W., Loos E. (2002). Blue-winged Teal: Anas Discors. The Birds of North America.

[44] Veits J., Weber S., Stech O., Breithaupt A., Gräber M., Gohrbandt S., Bogs J., Hundt J., Teifke J.P., Mettenleiter T.C. (2012). Avian influenza virus hemagglutinins H2, H4, H8, and H14 support a highly pathogenic phenotype. Proc. Natl. Acad. Sci. USA.

