# Supplementary material for: Blue-Winged Teals in Guatemala and Their Potential Role in the Ecology of H14 Subtype Influenza a Viruses
Source: Viruses. 2023 Feb 9;15(2):483. doi: 10.3390/v15020483 (PMC9961055; doi:10.3390/v15020483)
Supplement: Supplementary file 1 [file viruses-15-00483-s001.zip › Suppl_Table S10.pdf]

Suppl. Table S10. Detailed nucleotide pairwise identity of ORF sequences of NA N4 gene segment of full-length H14 viruses from Guatemala (n=40), North America (n=12), and Eurasia (n=4) during 1982-2019.

# virus	Strain	Reference ID	13	15	34	35	36	37	40
13	A/blue_winged_teal/Guatemala/CIP049H113_08/2013	KY644436							
15	A/blue_winged_teal/Guatemala/CIP049H113_76/2013	KY644370	100						
34	A/blue-winged_teal/Guatemala/CIP049H125_14/2015	OP144152	96.8	96.8					
35	A/blue-winged_teal/Guatemala/CIP049H125_23/2015	OP144160	97	97	99.6				
36	A/blue-winged_teal/Guatemala/CIP049H125_59/2015	OP144168	96.7	96.7	99.4	99.3			
37	A/blue-winged_teal/Guatemala/CIP049H125_108/2015	OP144176	96.9	96.9	99.5	99.9	99.2		
40	A/blue-winged_teal/Guatemala/CIP049H189_19/2019	OP144201	96.5	96.5	94.8	95	94.7	94.9	
