# Supplementary material for: Blue-Winged Teals in Guatemala and Their Potential Role in the Ecology of H14 Subtype Influenza a Viruses
Source: Viruses. 2023 Feb 9;15(2):483. doi: 10.3390/v15020483 (PMC9961055; doi:10.3390/v15020483)
Supplement: Supplementary file 1 [file viruses-15-00483-s001.zip › Suppl_Table S11.pdf]

Suppl. Table S11. Detailed nucleotide pairwise identity of ORF sequences of NA N5 gene segment of full-length H14 viruses from Guatemala (n=40), North America (n=12), and Eurasia (n=4) during 1982-2019.

# virus	Strain	Reference ID	19	20	21	31	41
**19**	A/blue_winged_teal/Guatemala/CIP049H117_36/2013	MK327704					
**20**	A/blue_winged_teal/Guatemala/CIP049H117_38/2013	MK326658	100				
**21**	A/blue_winged_teal/Guatemala/CIP049H117_99/2013	MK327736	99.9	99.9			
**31**	A/blue-winged_teal/Guatemala/CIP049H123_65/2014	OP144127	96.3	96.3	96.2		
41	A/Blue_winged_Teal/Ohio/18OS1695/2018	MN431051	94.7	94.7	94.7	97.3	
