# Supplementary material for: Blue-Winged Teals in Guatemala and Their Potential Role in the Ecology of H14 Subtype Influenza a Viruses
Source: Viruses. 2023 Feb 9;15(2):483. doi: 10.3390/v15020483 (PMC9961055; doi:10.3390/v15020483)
Supplement: Supplementary file 1 [file viruses-15-00483-s001.zip › Suppl_Table S12.pdf]

Table S12. Bayes Factor and Posterior Probability values using geographic location as a discrete trait

FROM	TO	BAYES FACTOR	POSTERIOR PROBABILITY
Guatemala	North America	0.691560319	0.360293
Guatemala	Eurasia	40.63620911	0.97067
North America	Eurasia	14.62882148	0.922564
North America	Guatemala	2.473435592	0.668259
Eurasia	Guatemala	1.785241748	0.59249
Eurasia	North America	11050.89079	1
