# Supplementary material for: Blue-Winged Teals in Guatemala and Their Potential Role in the Ecology of H14 Subtype Influenza a Viruses
Source: Viruses. 2023 Feb 9;15(2):483. doi: 10.3390/v15020483 (PMC9961055; doi:10.3390/v15020483)
Supplement: Supplementary file 1 [file viruses-15-00483-s001.zip › Suppl_Table S13.pdf]

Table S13. Bayes Factor and Posterior Probability values using HA cleavage site as a discrete trait

FROM	TO	BAYES FACTOR	POSTERIOR PROBABILITY
PDKQTRG	PDRQTRG	209.4707535	0.984668
PDKQTRG	PDKQTKG	0.581013868	0.151205
PDKQTRG	PDKQAKG	0.537749788	0.14154
PDKQTRG	PGKQAKG	0.583026727	0.15165
PDRQTRG	PDKQTKG	550.6451748	0.994112
PDRQTRG	PDKQAKG	0.462564191	0.124208
PDRQTRG	PGKQAKG	0.541194733	0.142318
PDKQTKG	PDKQAKG	3258.633522	0.999
PDKQTKG	PGKQAKG	0.975306058	0.230197
PDKQAKG	PGKQAKG	2.184035042	0.401067
PDRQTRG	PDKQTRG	0.771583905	0.191312
PDKQTKG	PDKQTRG	1.066501466	0.246417
PDKQAKG	PDKQTRG	3.079084075	0.485613
PGKQAKG	PDKQTRG	19.54892227	0.857016
PDKQTKG	PDRQTRG	0.786599645	0.194312
PDKQAKG	PDRQTRG	1.265384145	0.279524
PGKQAKG	PDRQTRG	0.790510884	0.195089
PDKQAKG	PDKQTKG	1.074171886	0.24775
PGKQAKG	PDKQTKG	0.836897534	0.2042
PGKQAKG	PDKQAKG	0.973472458	0.229863
