# Supplementary material for: Blue-Winged Teals in Guatemala and Their Potential Role in the Ecology of H14 Subtype Influenza a Viruses
Source: Viruses. 2023 Feb 9;15(2):483. doi: 10.3390/v15020483 (PMC9961055; doi:10.3390/v15020483)
Supplement: Supplementary file 1 [file viruses-15-00483-s001.zip › Suppl_Table S14.pdf]

Table S14. Bayes Factor and Posterior Probability values using Neuraminidase subtype as a discrete trait

FROM	TO	BAYES FACTOR	POSTERIOR PROBABILITY
N4	N3	4.554440094	0.420398
N4	Mixed	0.610010587	0.088546
N4	N5	0.690720359	0.0991
N4	N7	0.86787705	0.121431
N4	N6	13.87021671	0.688368
N4	N8	0.665882825	0.095878
N4	N2	0.581577309	0.084768
N3	Mixed	564.621093	0.989001
N3	N5	4703.648258	0.998667
N3	N7	7.082290755	0.530052
N3	N6	0.975207133	0.13443
N3	N8	0.396807819	0.059438
N3	N2	18833.43065	0.999667
Mixed	N5	0.99668647	0.136985
Mixed	N7	1.251657771	0.166204
Mixed	N6	1.255673696	0.166648
Mixed	N8	0.868780944	0.121542
Mixed	N2	1.105054865	0.14965
N5	N7	59.28823009	0.904233
N5	N6	0.709681453	0.101544
N5	N8	0.502547412	0.074103
N5	N2	0.496043693	0.073214
N7	N6	3.591407322	0.363848
N7	N8	507.5310631	0.987779
N7	N2	0.3374099	0.050994
N6	N8	0.418167264	0.062438
N6	N2	0.412616517	0.06166
N8	N2	1.177143951	0.157871
N3	N4	0.858850677	0.12032
Mixed	N4	0.99762324	0.137096
N5	N4	0.589915645	0.085879
N7	N4	7.187348982	0.533718
N6	N4	4.390820752	0.41151
N8	N4	1.848365524	0.227419
N2	N4	1.091572908	0.148095
Mixed	N3	1.931010785	0.235196
N5	N3	0.612530725	0.088879
N7	N3	1.214712406	0.162093
N6	N3	289.6324675	0.97878

N8	N3	1.502582862	0.19309
N2	N3	1.122461451	0.15165
N5	Mixed	0.478222616	0.07077
N7	Mixed	0.330445856	0.049994
N6	Mixed	0.411032279	0.061438
N8	Mixed	1.10988181	0.150206
N2	Mixed	5.802694696	0.48028
N7	N5	0.648856376	0.093656
N6	N5	0.466930662	0.069215
N8	N5	1.369884886	0.179091
N2	N5	1.207763482	0.161315
N6	N7	0.543480252	0.079658
N8	N7	1.777690992	0.220642
N2	N7	1.425146853	0.184979
N8	N6	2.06433287	0.247417
N2	N6	1.320516008	0.173758
N2	N8	0.994813653	0.136763
