# Supplementary material for: Blue-Winged Teals in Guatemala and Their Potential Role in the Ecology of H14 Subtype Influenza a Viruses
Source: Viruses. 2023 Feb 9;15(2):483. doi: 10.3390/v15020483 (PMC9961055; doi:10.3390/v15020483)
Supplement: Supplementary file 1 [file viruses-15-00483-s001.zip › Suppl_Table S15.pdf]

Table S15. Best ten Blast hits of A/blue-winged-teal/Guatemala/CIP049H117-34/2013 and A/blue-winged-teal/Guatemala/CIP049H117-143/2013

**Identity (%)**	**Blast hit**	**GeneBank accession no.**
99.666	A/mallard/Ohio/13OS1979/2013(H10N7)	KX013057.1
99.599	A/blue-winged teal/Iowa/13OS2367/2013(H4N8)	KJ568242.1
99.532	A/American black duck/Maryland/13OS2956/2013(H11N9)	CY191278.1
99.532	A/blue-winged teal/Iowa/13OS2339/2013(H4N8)	KJ568074.1
99.465	A/mallard/Ontario/13OS4098/2013(H10N3)	KX013063.1
99.465	A/mallard/Maryland/13OS2946/2013(H5N2)	CY191270.1
99.198	A/blue-winged teal/Ohio/13OS2064/2013(H4N6)	KJ568290.1
99.198	A/American green-winged teal/Ohio/13OS2065/2013(H4N6)	KJ568418.1
99.132	A/American green-winged teal/Ohio/13OS2063/2013(H4N6)	KJ567994.1
98.93	A/American green-winged teal/Alaska/137916/2009(H3N8)	KX714451.1
