# Supplementary material for: Blue-Winged Teals in Guatemala and Their Potential Role in the Ecology of H14 Subtype Influenza a Viruses
Source: Viruses. 2023 Feb 9;15(2):483. doi: 10.3390/v15020483 (PMC9961055; doi:10.3390/v15020483)
Supplement: Supplementary file 1 [file viruses-15-00483-s001.zip › Suppl_Table S4.pdf]

Suppl. Table S4. Detailed nucleotide pairwise identity of ORF sequences of PA gene segment of full-length H14 viruses from Guatemala (n=40), North America (n=12), and Eurasia (n=4) during 1982-2019.

# virus	Strain	Reference ID	1-A	1-B	2	3	4	5	6	7	8	9	10	11	12	13	14	15	16	17	18	19	20	21	22	23	24	25	26	27	28	29	30	31	32	33	34	35	36	37	38	39	40	41	42	43	44	45	46	47	48	49	50	51	52	53	54	55	56
**1-A (variant #1)**	A/blue_winged_teal/Guatemala/CIP049H105_15/2011	KJ195675
**1-B (variant #2)**	A/blue_winged_teal/Guatemala/CIP049H105_15/2011	KJ195681	95.9
**2**	A/blue_winged_teal/Guatemala/CIP049H105_31/2011	KY644429	87.5	87.7
**3**	A/blue_winged_teal/Guatemala/CIP049H106_62/2011	KJ195667	88.1	88.3	94.7
**4**	A/blue_winged_teal/Guatemala/CIP049H108_02/2012	KY644494	90.7	90	87.5	88
**5**	A/blue_winged_teal/Guatemala/CIP049H108_04/2012	KX960446	90.6	89.9	87.4	88	99.7
**6**	A/blue_winged_teal/Guatemala/CIP049H108_11/2012	KX960391	90.5	89.8	87.3	87.9	99.6	99.8
**7**	A/blue_winged_teal/Guatemala/CIP049H109_49/2012	KY644237	90.6	89.9	87.4	88	99.7	99.9	99.9
**8**	A/blue_winged_teal/Guatemala/CIP049H109_67/2012	KY673174	90.9	90.6	88.3	88.7	94.7	94.7	94.6	94.7
**9**	A/blue_winged_teal/Guatemala/CIP049H109_76/2012	KX960370	90.7	89.9	87.4	87.9	99.7	99.9	99.9	100	94.7
**10**	A/blue_winged_teal/Guatemala/CIP049H110_04/2012	KY644201	88	88	97.6	95.1	87.8	87.7	87.6	87.7	88.5	87.6
**11**	A/blue_winged_teal/Guatemala/CIP049H110_31/2012	KY644507	88	88.1	94.6	99.2	88	87.9	87.8	87.9	88.6	87.8	95
**12**	A/blue_winged_teal/Guatemala/CIP049H113_07/2013	KY644322	90.7	90	87.5	88.1	99.8	99.6	99.5	99.6	94.7	99.6	87.8	88
**13**	A/blue_winged_teal/Guatemala/CIP049H113_08/2013	KY644375	90.7	90	87.6	88.1	99.8	99.7	99.6	99.7	94.9	99.7	87.9	88	99.7
**14**	A/blue_winged_teal/Guatemala/CIP049H113_74/2013	KY673163	90.7	90	87.4	88	99.8	99.7	99.6	99.7	94.7	99.7	87.7	87.9	99.7	99.8
**15**	A/blue_winged_teal/Guatemala/CIP049H113_76/2013	KY644229	90.7	90	87.6	88.1	99.8	99.7	99.6	99.7	94.9	99.7	87.9	88	99.7	100	99.8
**16**	A/blue_winged_teal/Guatemala/CIP049H116_120/2013	MK327717	87.9	88.1	97.3	94.6	87.9	87.9	87.8	87.9	88.4	87.8	97.2	94.6	87.9	88	87.8	88
**17**	A/blue_winged_teal/Guatemala/CIP049H117_13/2013	MK327685	87.8	88	97.3	94.6	87.8	87.8	87.7	87.8	88.3	87.8	97.2	94.5	87.8	87.9	87.7	87.9	100
**18**	A/blue_winged_teal/Guatemala/CIP049H117_34/2013	MK327693	87.6	88	97.2	94.6	87.6	87.6	87.5	87.6	88.3	87.5	97.2	94.6	87.6	87.7	87.5	87.7	98.5	98.6
**19**	A/blue_winged_teal/Guatemala/CIP049H117_36/2013	MK327701	88.2	88.4	94	98.1	88.3	88.2	88.1	88.2	89.1	88.2	94.4	98.1	88.4	88.4	88.2	88.4	94.2	94.1	94.1
**20**	A/blue_winged_teal/Guatemala/CIP049H117_38/2013	MK326655	88.2	88.4	94	98.1	88.3	88.2	88.1	88.2	89.1	88.2	94.4	98.1	88.4	88.4	88.2	88.4	94.2	94.1	94.1	100
**21**	A/blue_winged_teal/Guatemala/CIP049H117_99/2013	MK327733	87.6	88	97.3	94.7	87.5	87.5	87.4	87.5	88.4	87.4	97.2	94.7	87.5	87.6	87.4	87.6	98.6	98.6	99.7	94.3	94.3
**22**	A/blue_winged_teal/Guatemala/CIP049H117_123/2013	MK327725	87.8	88	97.3	94.6	87.8	87.8	87.7	87.8	88.3	87.8	97.2	94.5	87.8	87.9	87.7	87.9	100	99.9	98.5	94.1	94.1	98.5
**23**	A/blue_winged_teal/Guatemala/CIP049H117_143/2013	MK327757	87.6	88	97.1	94.5	87.6	87.6	87.5	87.6	88.3	87.5	97.1	94.6	87.6	87.7	87.5	87.7	98.5	98.5	100	94.1	94.1	99.6	98.4
**24**	A/blue-winged_teal/Guatemala/CIP049H123_13/2014	OP144067	88.1	88.3	96.7	94	87.5	87.5	87.4	87.5	88.4	87.5	96.8	94.1	87.5	87.6	87.4	87.6	98	98	99.1	93.6	93.6	99.1	98	99
**25**	A/blue-winged_teal/Guatemala/CIP049H123_16/2014	OP144075	88	87.9	94.2	97.7	87.6	87.5	87.4	87.5	88.4	87.5	94.9	97.8	87.6	87.7	87.6	87.7	94.3	94.2	94.2	96.7	96.7	94.4	94.2	94.2	94
**26**	A/blue-winged_teal/Guatemala/CIP049H123_33/2014	OP144083	90.7	90.4	88.1	88.4	94.7	94.7	94.6	94.7	99.1	94.7	88.3	88.3	94.7	94.9	94.7	94.9	88.1	88	88	88.8	88.8	88	88	88	88.1	88.2
**27**	A/blue-winged_teal/Guatemala/CIP049H123_57/2014	OP144091	88.1	88.3	96.7	94	87.5	87.5	87.4	87.5	88.4	87.5	96.8	94.1	87.5	87.6	87.4	87.6	98	98	99.1	93.6	93.6	99.1	98	99	100	94	88.1
**28**	A/blue-winged_teal/Guatemala/CIP049H123_58/2014	OP144099	88.1	88.2	96.7	94	87.5	87.5	87.4	87.5	88.3	87.4	96.8	94.1	87.5	87.6	87.4	87.6	98	98	99	93.6	93.6	99.1	97.9	99	100	94	88	100
**29**	A/blue-winged_teal/Guatemala/CIP049H123_59/2014	OP144107	88.1	88.3	96.7	94	87.5	87.5	87.4	87.5	88.4	87.5	96.8	94.1	87.5	87.6	87.4	87.6	98	98	99.1	93.6	93.6	99.1	98	99	100	94	88.1	100	100
**30**	A/blue-winged_teal/Guatemala/CIP049H123_61/2014	OP144115	88.1	88.3	96.7	94	87.5	87.5	87.4	87.5	88.4	87.5	96.8	94.1	87.5	87.6	87.4	87.6	98	98	99.1	93.6	93.6	99.1	98	99	100	94	88.1	100	100	100
**31**	A/blue-winged_teal/Guatemala/CIP049H123_65/2014	OP144123	88.1	88.3	96.7	94	87.5	87.5	87.4	87.5	88.4	87.5	96.8	94.1	87.5	87.6	87.4	87.6	98	98	99.1	93.6	93.6	99.1	98	99	100	94	88.1	100	100	100	100
**32**	A/blue-winged_teal/Guatemala/CIP049H123_75/2014	OP144132	88	87.9	94.2	97.7	87.6	87.5	87.4	87.5	88.4	87.5	94.9	97.8	87.6	87.7	87.6	87.7	94.3	94.2	94.2	96.7	96.7	94.4	94.2	94.2	94	100	88.2	94	94	94	94	94
**33**	A/blue-winged_teal/Guatemala/CIP049H123_76/2014	OP144140	88.1	88.3	96.7	94	87.5	87.5	87.4	87.5	88.4	87.5	96.8	94.1	87.5	87.6	87.4	87.6	98	98	99.1	93.6	93.6	99.1	98	99	100	94	88.1	100	100	100	100	100	94
**34**	A/blue-winged_teal/Guatemala/CIP049H125_14/2015	OP144149	88	88.1	96.8	94.3	87.7	87.7	87.6	87.7	88.7	87.7	96.8	94.3	87.7	87.8	87.6	87.8	98.2	98.3	99.2	94	94	99.3	98.2	99.2	99	94	88.2	99	99	99	99	99	94	99
**35**	A/blue-winged_teal/Guatemala/CIP049H125_23/2015	OP144157	87.9	88	96.7	94.1	87.6	87.6	87.5	87.6	88.6	87.6	96.7	94.1	87.5	87.7	87.5	87.7	98	98.1	99	93.9	93.9	99.1	98	99	98.8	93.9	88.1	98.8	98.8	98.8	98.8	98.8	93.9	98.8	99.6
**36**	A/blue-winged_teal/Guatemala/CIP049H125_59/2015	OP144165	87.9	88	96.7	94.1	87.5	87.5	87.4	87.5	88.6	87.6	96.8	94.1	87.4	87.6	87.4	87.6	98.1	98.1	99.1	93.8	93.8	99.1	98	99	98.9	93.9	88.1	98.9	98.8	98.9	98.9	98.9	93.9	98.9	99.6	100
**37**	A/blue-winged_teal/Guatemala/CIP049H125_108/2015	OP144173	88.2	88.3	96.6	94	87.5	87.5	87.4	87.5	88.2	87.4	96.6	94	87.5	87.5	87.4	87.5	97.8	97.8	98.8	93.5	93.5	98.9	97.7	98.8	99.8	93.8	88	99.8	99.7	99.8	99.8	99.8	93.8	99.8	98.9	98.7	98.7
**38**	A/blue-winged_teal/Guatemala/CIP049H158_26/2019	OP144181	88	88	95.7	93.6	87.5	87.5	87.4	87.5	88.1	87.5	96	93.7	87.5	87.6	87.4	87.6	97.1	97.1	97.8	93.4	93.4	97.9	97.1	97.8	97.6	93.3	87.9	97.6	97.6	97.6	97.6	97.6	93.3	97.6	98.2	98.1	98.2	97.4
**39**	A/blue-winged_teal/Guatemala/CIP049H189_08/2019	OP144189	87.5	87.6	95.9	93.6	87.2	87.2	87.1	87.2	88	87.2	96.2	93.7	87.2	87.3	87.1	87.3	97.5	97.5	98.3	93.2	93.2	98.3	97.4	98.2	98.2	93.4	87.7	98.2	98.1	98.2	98.2	98.2	93.4	98.2	98.8	98.6	98.7	98	97.4
**40**	A/blue-winged_teal/Guatemala/CIP049H189_19/2019	OP144198	87.5	87.8	96	93.7	87.6	87.6	87.5	87.6	88.2	87.5	96.1	93.8	87.6	87.7	87.5	87.7	97.4	97.4	98.3	93.3	93.3	98.3	97.3	98.2	97.8	93.6	87.9	97.8	97.8	97.8	97.8	97.8	93.6	97.8	98	97.9	97.8	97.9	96.7	97.1
41	A/Blue_winged_Teal/Ohio/18OS1695/2018	MN431049	87.5	88.1	95.9	94	87.4	87.3	87.3	87.3	88.7	87.4	96	94	87.4	87.5	87.3	87.5	96.3	96.4	96.2	93.7	93.7	96.4	96.3	96.1	95.8	93.8	88.5	95.8	95.8	95.8	95.8	95.8	93.8	95.8	95.9	95.8	95.9	95.6	95.2	95.3	95.3
42	A/blue_winged_teal/Texas/UGAI15_6890/2015	KX024582	87.5	88.1	96	94.2	87.5	87.5	87.5	87.5	88.5	87.5	96.1	94.2	87.5	87.7	87.5	87.7	96.3	96.4	96.4	93.8	93.8	96.5	96.3	96.3	96	94.1	88.2	96	96	96	96	96	94.1	96	96.1	96	96	95.8	95.2	95.3	95.5	97.6
43	A/long_tailed_duck/Wisconsin/10OS3912/2010	KC110596	95.6	96	87.4	88.4	90.1	89.9	89.8	89.9	90.7	90	87.9	88.3	90.1	90.1	90.1	90.1	87.5	87.5	87.5	88.4	88.4	87.6	87.5	87.5	87.9	88.4	90.4	87.9	87.9	87.9	87.9	87.9	88.4	87.9	87.7	87.8	87.8	87.9	87.9	87.5	87.2	87.8	87.9
44	A/long_tailed_duck/Wisconsin/10OS3918/2010	CY133558	95.9	96.1	87.6	88.6	90.2	90	90	90	90.8	90.1	88	88.5	90.2	90.2	90.2	90.2	87.8	87.7	87.6	88.6	88.6	87.7	87.7	87.6	88	88.6	90.6	88	88	88	88	88	88.6	88	87.9	87.9	88	88	88	87.7	87.3	87.9	88	99.6
45	A/long_tailed_duck/Wisconsin/10OS4225/2010	KC110604	95.8	96	87.5	88.5	90.2	90	90	90	90.7	90.1	88	88.4	90.2	90.2	90.2	90.2	87.7	87.7	87.6	88.5	88.5	87.7	87.7	87.6	88	88.5	90.5	88	88	88	88	88	88.5	88	87.8	87.9	87.9	88	88	87.6	87.2	87.9	88	99.6	99.9
46	A/mallard/Wisconsin/10OS3941/2010	CY133271	95.9	96.1	87.6	88.6	90.3	90.1	90	90.1	90.8	90.1	88.1	88.5	90.3	90.3	90.3	90.3	87.8	87.8	87.7	88.6	88.6	87.8	87.8	87.7	88.1	88.6	90.6	88.1	88	88.1	88.1	88.1	88.6	88.1	87.9	88	88	88	88	87.7	87.3	88	88	99.7	100	99.9
47	A/northern_shoveler/California/2696/2011	CY146902	90.4	90.3	87	87.4	94	93.8	93.7	93.8	93.4	93.7	87	87.2	93.9	94	94	94	87.1	87.1	86.8	87.5	87.5	86.7	87.1	86.8	86.7	87.4	93.5	86.7	86.7	86.7	86.7	86.7	87.4	86.7	87.1	87.1	87	86.6	87	86.5	86.7	87.4	87.2	90.5	90.5	90.5	90.6
48	A/Northern_Shoveler/California/D1616217/2016	MK928233	90.2	90.2	88	88.6	94.2	94	94	94	97.9	94.1	88	88.5	94.1	94.3	94.1	94.3	88.2	88.1	88.2	89	89	88.3	88.1	88.2	88.3	88.2	97.6	88.3	88.2	88.3	88.3	88.3	88.2	88.3	88.3	88.4	88.4	88.2	88	87.8	88	88.2	88	90.1	90.3	90.2	90.3	92.8
49	A/northern_shoveler/Mississippi/12OS456/2012	CY167272	90.7	90.4	87.2	87.4	92.7	92.7	92.6	92.7	93.3	92.6	87.6	87.4	92.7	92.8	92.8	92.8	87.5	87.5	87.3	87.8	87.8	87.4	87.5	87.3	87.3	87.5	93.3	87.3	87.3	87.3	87.3	87.3	87.5	87.3	87.6	87.5	87.5	87.3	87.4	86.9	87.2	87.5	87.9	90.8	90.7	90.8	90.8	94.9	92.7
50	A/northern_shoveler/Missouri/10OS4673/2010	CY133386	91.9	91.3	87.6	87.8	94.1	94	93.9	94	94.2	94	87.9	87.6	94.1	94.1	94	94.1	87.8	87.8	87.4	88	88	87.5	87.8	87.4	87.4	87.7	94.2	87.4	87.4	87.4	87.4	87.4	87.7	87.4	87.7	87.8	87.7	87.4	87.7	87	87.3	87.8	87.8	91.8	91.9	91.9	91.9	96	93.4	96.6
51	A/Northern_shoveler/Missouri/16OS6248/2016	MG280558	95.6	98.3	87.9	88.5	90.1	89.9	89.8	89.9	90.8	90	88.1	88.4	90.1	90.1	90	90.1	88.1	88.1	88	88.7	88.7	88	88.1	88	88.3	88.1	90.7	88.3	88.3	88.3	88.3	88.3	88.1	88.3	88.1	88	88	88.4	88	87.6	87.7	88	88	95.3	95.6	95.5	95.6	90.1	90.2	90.5	91.4
52	A/white_winged_scoter/Wisconsin/10OS3922/2010	KC110610	95.8	96	87.5	88.5	90.1	90	89.9	90	90.8	90	88	88.4	90.1	90.1	90.1	90.1	87.7	87.6	87.5	88.5	88.5	87.6	87.6	87.5	88	88.4	90.6	88	87.9	88	88	88	88.4	88	87.8	87.8	87.9	87.9	87.9	87.6	87.2	87.8	87.9	99.5	99.8	99.8	99.9	90.5	90.3	90.7	92	95.5
53	A/Common_Teal/Chany_Lake/29/2019	EPI_ISL_400267	91	90.2	87.6	87.1	90.3	90.2	90.1	90.2	90.3	90.2	88	87.2	90.2	90.2	90.2	90.2	88.3	88.2	88	87.4	87.4	88	88.2	88	88.2	87.3	90.3	88.2	88.1	88.2	88.2	88.2	87.3	88.2	88.2	88.2	88.2	88.2	88.4	87.8	87.8	87.7	87.6	90.1	90.1	90.1	90.2	89.7	90	90	90.6	89.9	90.1
54	A/garganey/Ukraine/05839-NAMRU3/2006	EPI_ISL_120215	91	90.6	87.5	87.3	90.1	90.1	90	90.1	89.7	90.1	87.8	87.3	90	90.1	90	90.1	87.9	87.8	87.6	87.3	87.3	87.6	87.8	87.6	87.8	87.2	89.6	87.8	87.8	87.8	87.8	87.8	87.2	87.8	87.8	87.8	87.7	87.8	88	87.5	87.6	87.5	87.3	90.5	90.7	90.6	90.7	89.9	89.3	90.2	90.7	90.2	90.7	94.2
55	A/goose/Karachi/NARC_13N_969/2014	KX602667	91.2	90.7	87.8	87.6	90.1	90	89.9	90	90.1	90	88.5	87.6	90	90.1	90	90.1	88.8	88.8	88.6	87.8	87.8	88.5	88.8	88.6	88.8	87.7	90.2	88.8	88.8	88.8	88.8	88.8	87.7	88.8	88.7	88.8	88.7	88.8	89	88.5	88.4	87.9	87.9	90.8	91	90.9	91	90.4	89.7	90.1	91.1	90.3	91	95.7	95.5
56	A/mallard/Astrakhan/263/1982	CY130099	92.2	91.9	87.9	88	90.7	90.7	90.7	90.7	90.6	90.8	88.4	87.9	90.7	90.7	90.7	90.7	88.5	88.5	88.1	88.3	88.3	88	88.5	88.1	88.1	87.8	90.7	88.1	88.1	88.1	88.1	88.1	87.8	88.1	88.3	88.3	88.3	88.1	88.7	87.7	87.7	87.8	88	92.1	92.2	92.2	92.3	90.7	90.4	90.9	91.7	91.3	92.2	93.5	94.6	94.5
