# Supplementary material for: Blue-Winged Teals in Guatemala and Their Potential Role in the Ecology of H14 Subtype Influenza a Viruses
Source: Viruses. 2023 Feb 9;15(2):483. doi: 10.3390/v15020483 (PMC9961055; doi:10.3390/v15020483)
Supplement: Supplementary file 1 [file viruses-15-00483-s001.zip › Suppl_Table S9.pdf]

Suppl. Table S9. Detailed nucleotide pairwise identity of ORF sequences of NA N3 gene segment of full-length H14 viruses from Guatemala (n=40), North America (n=12), and Eurasia (n=4) during 1982-2019.

# virus	Strain	Reference ID	1	2	4	5	6	7	9	11	12	14	16	22	17	23	18	24	25	26	27	28	29	30	31	32	33	38	39	48	53	55
1	A/blue_winged_teal/Guatemala/CIP049H105_15/2011	KJ195678																														
2	A/blue_winged_teal/Guatemala/CIP049H105_31/2011	KY644491	94.5																													
4	A/blue_winged_teal/Guatemala/CIP049H108_02/2012	KY644425	95.4	95																												
5	A/blue_winged_teal/Guatemala/CIP049H108_04/2012	KX960401	95.5	95.1	99.9																											
6	A/blue_winged_teal/Guatemala/CIP049H108_11/2012	KX960439	95.5	95	99.8	99.9																										
7	A/blue_winged_teal/Guatemala/CIP049H109_49/2012	KY644327	95.5	95.1	99.9	100	99.9																									
9	A/blue_winged_teal/Guatemala/CIP049H109_76/2012	KX960432	95.5	95.1	99.9	100	99.9	100																								
11	A/blue_winged_teal/Guatemala/CIP049H110_31/2012	KY644476	95.6	95.2	99.5	99.6	99.6	99.6	99.6																							
12	A/blue_winged_teal/Guatemala/CIP049H113_07/2013	KY644194	95.4	95	99.9	99.9	99.8	99.9	99.9	99.5																						
14	A/blue_winged_teal/Guatemala/CIP049H113_74/2013	KY673158	95.3	94.9	99.8	99.8	99.7	99.8	99.8	99.4	99.8																					
16	A/blue_winged_teal/Guatemala/CIP049H116_120/2013	MK327720	95	94.5	98.4	98.6	98.5	98.6	98.6	98.7	98.4	98.4																				
22	A/blue_winged_teal/Guatemala/CIP049H117_13/2013	MK327688	95.1	94.5	98.4	98.6	98.5	98.6	98.6	98.7	98.4	98.4	99.9																			
17	A/blue_winged_teal/Guatemala/CIP049H117_34/2013	MK327696	94.8	94.4	98.3	98.4	98.4	98.4	98.4	98.5	98.3	98.2	99.7	99.7																		
23	A/blue_winged_teal/Guatemala/CIP049H117_123/2013	MK327728	95	94.6	98.5	98.7	98.6	98.7	98.7	98.7	98.5	98.4	99.9	99.9	99.8																	
18	A/blue_winged_teal/Guatemala/CIP049H117_143/2013	MK327760	95	94.6	98.5	98.7	98.6	98.7	98.7	98.7	98.5	98.4	99.9	99.9	99.8	100																
24	A/blue-winged_teal/Guatemala/CIP049H123_13/2014	OP144070	94.7	94.2	98.4	98.5	98.4	98.5	98.5	98.6	98.4	98.3	97.5	97.5	97.4	97.6	97.6															
25	A/blue-winged_teal/Guatemala/CIP049H123_16/2014	OP144078	94.7	94.1	98.3	98.4	98.4	98.4	98.4	98.5	98.3	98.2	97.4	97.4	97.3	97.5	97.5	99.6														
26	A/blue-winged_teal/Guatemala/CIP049H123_33/2014	OP144086	94.7	94.2	98.4	98.5	98.4	98.5	98.5	98.6	98.4	98.3	97.5	97.5	97.4	97.6	97.6	100	99.6													
27	A/blue-winged_teal/Guatemala/CIP049H123_57/2014	OP144094	94.7	94.2	98.4	98.5	98.4	98.5	98.5	98.6	98.4	98.3	97.5	97.5	97.4	97.6	97.6	100	99.6	100												
28	A/blue-winged_teal/Guatemala/CIP049H123_58/2014	OP144102	94.7	94.2	98.4	98.5	98.4	98.5	98.5	98.6	98.4	98.3	97.5	97.5	97.4	97.6	97.6	100	99.6	100	100											
29	A/blue-winged_teal/Guatemala/CIP049H123_59/2014	OP144110	94.7	94.2	98.4	98.5	98.4	98.5	98.5	98.6	98.4	98.3	97.5	97.5	97.4	97.6	97.6	100	99.6	100	100	100										
30	A/blue-winged_teal/Guatemala/CIP049H123_61/2014	OP144118	94.7	94.2	98.4	98.5	98.4	98.5	98.5	98.6	98.4	98.3	97.5	97.5	97.4	97.6	97.6	100	99.6	100	100	100	100									
31	A/blue-winged_teal/Guatemala/CIP049H123_65/2014	OP144126	94.7	94.1	98.3	98.4	98.4	98.4	98.4	98.5	98.3	98.2	97.4	97.4	97.3	97.5	97.5	99.9	99.5	99.9	99.9	99.9	99.9	99.9								
32	A/blue-winged_teal/Guatemala/CIP049H123_75/2014	OP144135	94.7	94.1	98.3	98.4	98.4	98.4	98.4	98.5	98.3	98.2	97.4	97.4	97.3	97.5	97.5	99.6	99.9	99.6	99.6	99.6	99.6	99.6	99.6							
33	A/blue-winged_teal/Guatemala/CIP049H123_76/2014	OP144144	94.7	94.2	98.4	98.5	98.4	98.5	98.5	98.6	98.4	98.3	97.5	97.5	97.4	97.6	97.6	100	99.6	100	100	100	100	100	99.9	99.6						
38	A/blue-winged_teal/Guatemala/CIP049H158_26/2019	OP144184	93.9	93.9	97.1	97.2	97.2	97.2	97.2	97.4	97.1	97	96.8	96.8	96.7	96.9	96.9	97	96.8	97	97	97	97	97	96.9	96.8	97					
39	A/blue-winged_teal/Guatemala/CIP049H189_08/2019	OP144193	94.8	94.3	97.7	97.8	97.7	97.8	97.8	97.9	97.7	97.6	96.9	96.9	96.8	97	97	97.7	97.7	97.7	97.7	97.7	97.7	97.7	97.7	97.7	97.7	96.2				
48	A/Northern_Shoveler/California/D1616217/2016	MK928230	96.3	92.4	93.5	93.7	93.6	93.7	93.7	93.8	93.5	93.5	93.3	93.4	93.1	93.3	93.3	93	93.1	93	93	93	93	93	93.1	93.1	93	92.2	93			
53	A/Common_Teal/Chany_Lake/29/2019	EPI_ISL_400267	84.9	85.2	84.8	84.9	84.8	84.9	84.9	84.9	84.8	84.8	84.9	84.8	84.7	84.9	84.9	84.8	84.7	84.8	84.8	84.8	84.8	84.8	84.8	84.7	84.8	85.5	84.9	84.8		
55	A/goose/Karachi/NARC_13N_969/2014	KX602670	85.2	85.6	84.9	85	85	85	85	85	85	85	85.2	85.1	85.2	85.2	85.2	85.3	85.2	85.3	85.3	85.3	85.3	85.3	85.2	85.2	85.3	85.5	85.4	85.4	93.3	
