# Supplementary figures and images for: Blue-Winged Teals in Guatemala and Their Potential Role in the Ecology of H14 Subtype Influenza a Viruses
Source: Viruses. 2023 Feb 9;15(2):483. doi: 10.3390/v15020483 (PMC9961055; doi:10.3390/v15020483)

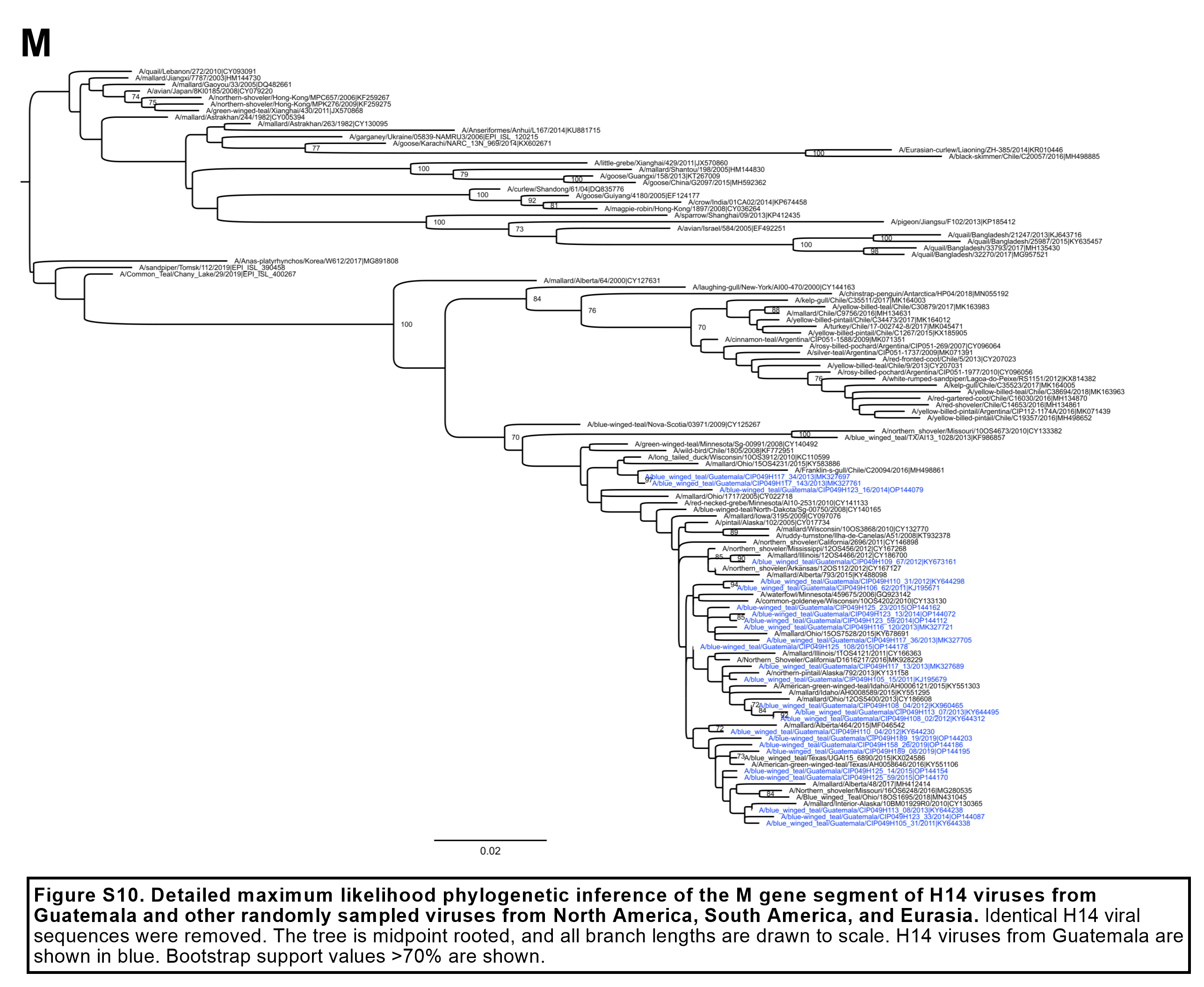

Supplement: Supplementary file 1 [file viruses-15-00483-s001.zip › Suppl_Figure S10_w_legend.tif]

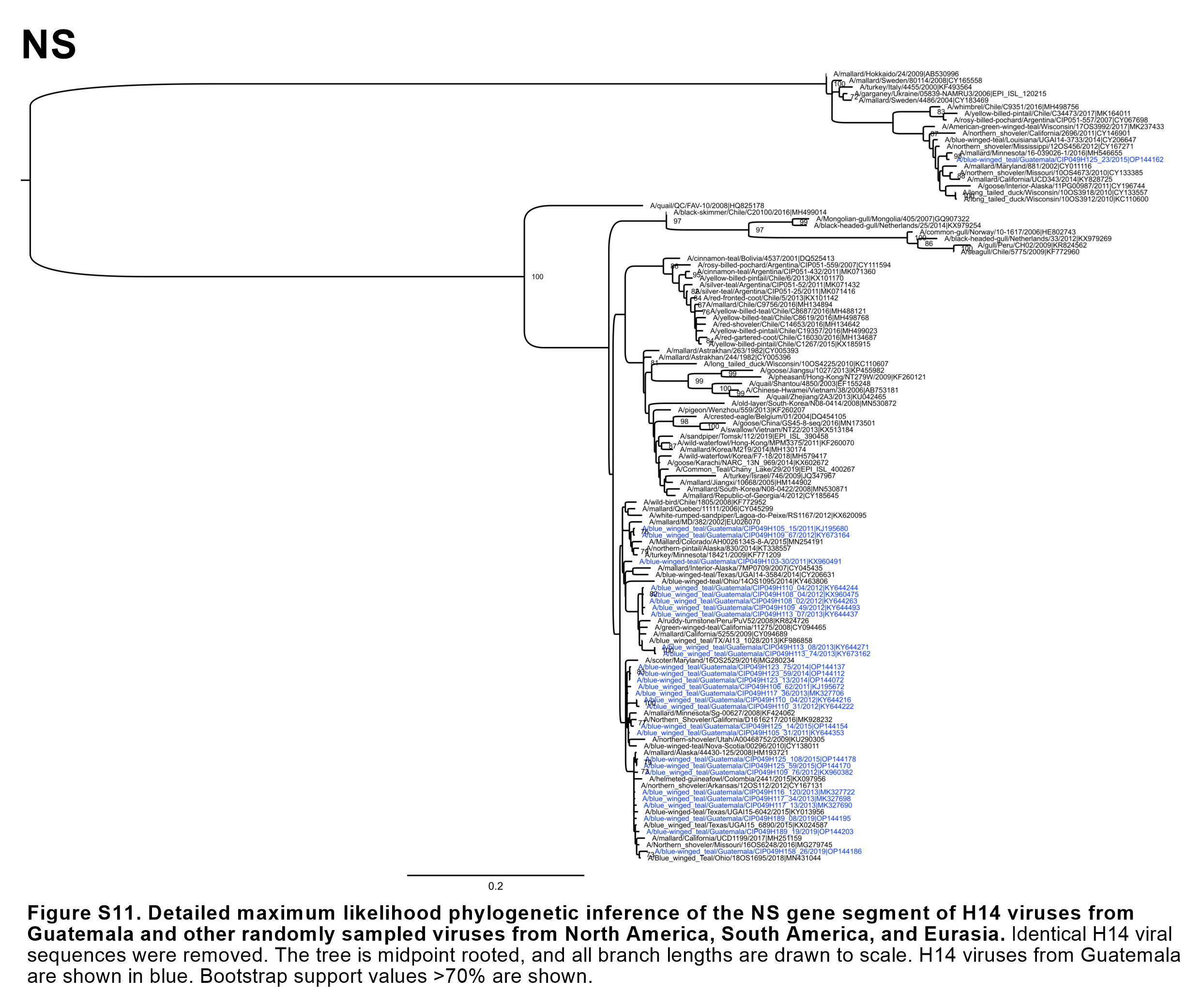

Supplement: Supplementary file 1 [file viruses-15-00483-s001.zip › Suppl_Figure S11_w_legend.tif]

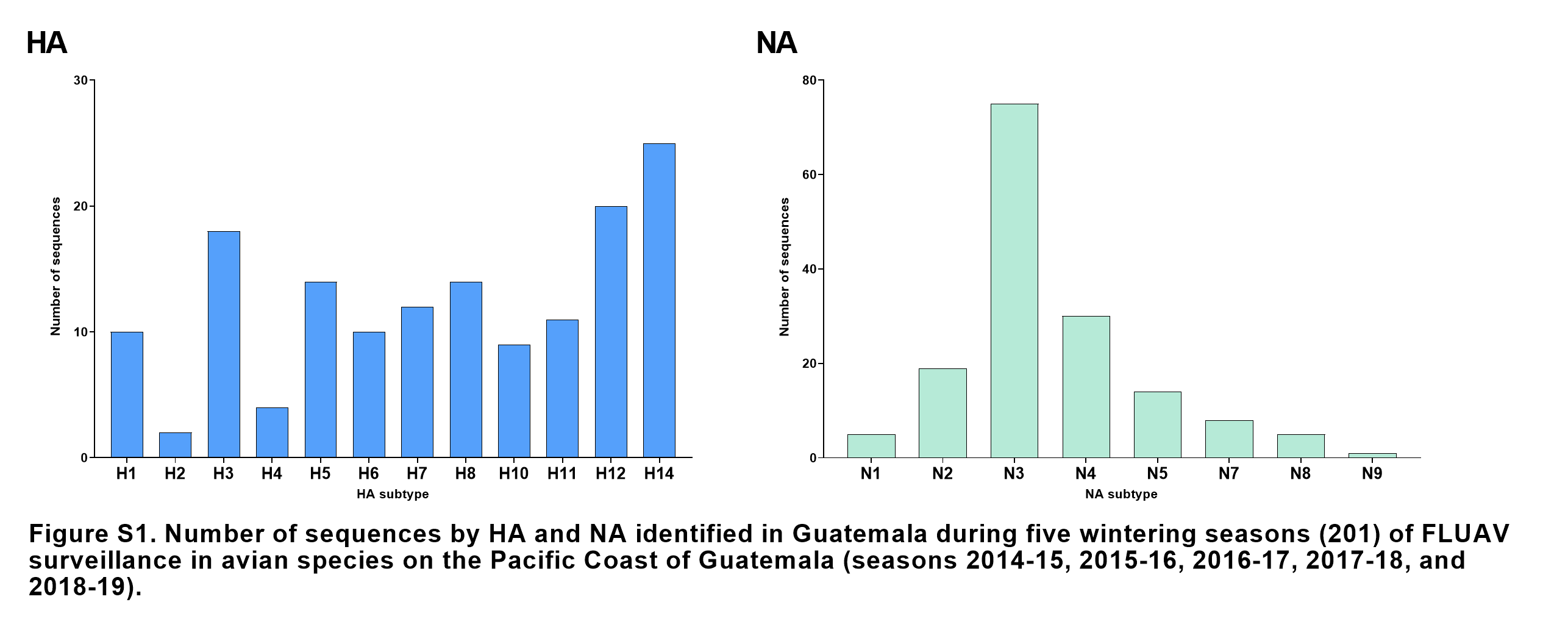

Supplement: Supplementary file 1 [file viruses-15-00483-s001.zip › Suppl_Figure S1_w_legend.tif]

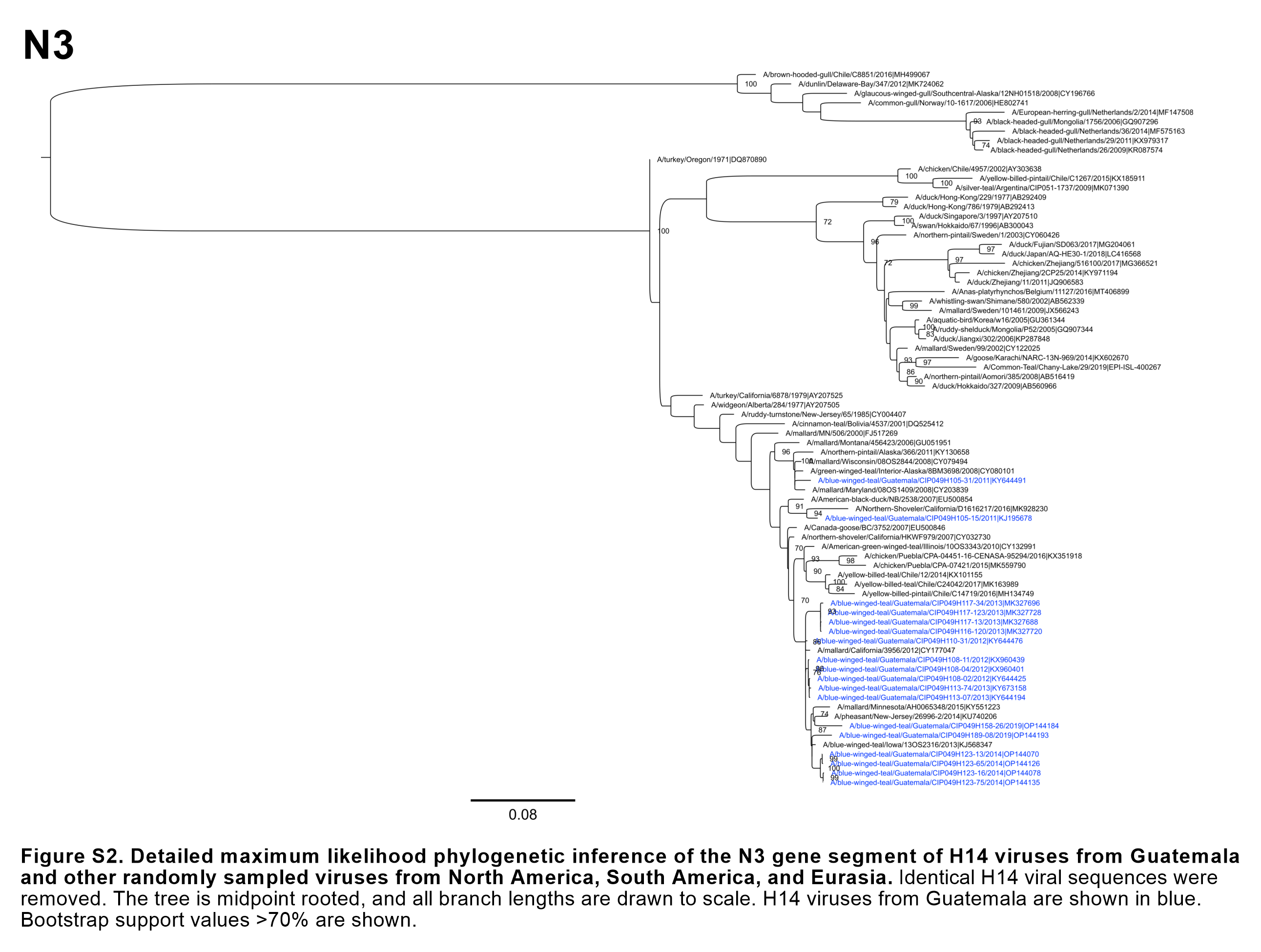

Supplement: Supplementary file 1 [file viruses-15-00483-s001.zip › Suppl_Figure S2_w_legend.tif]

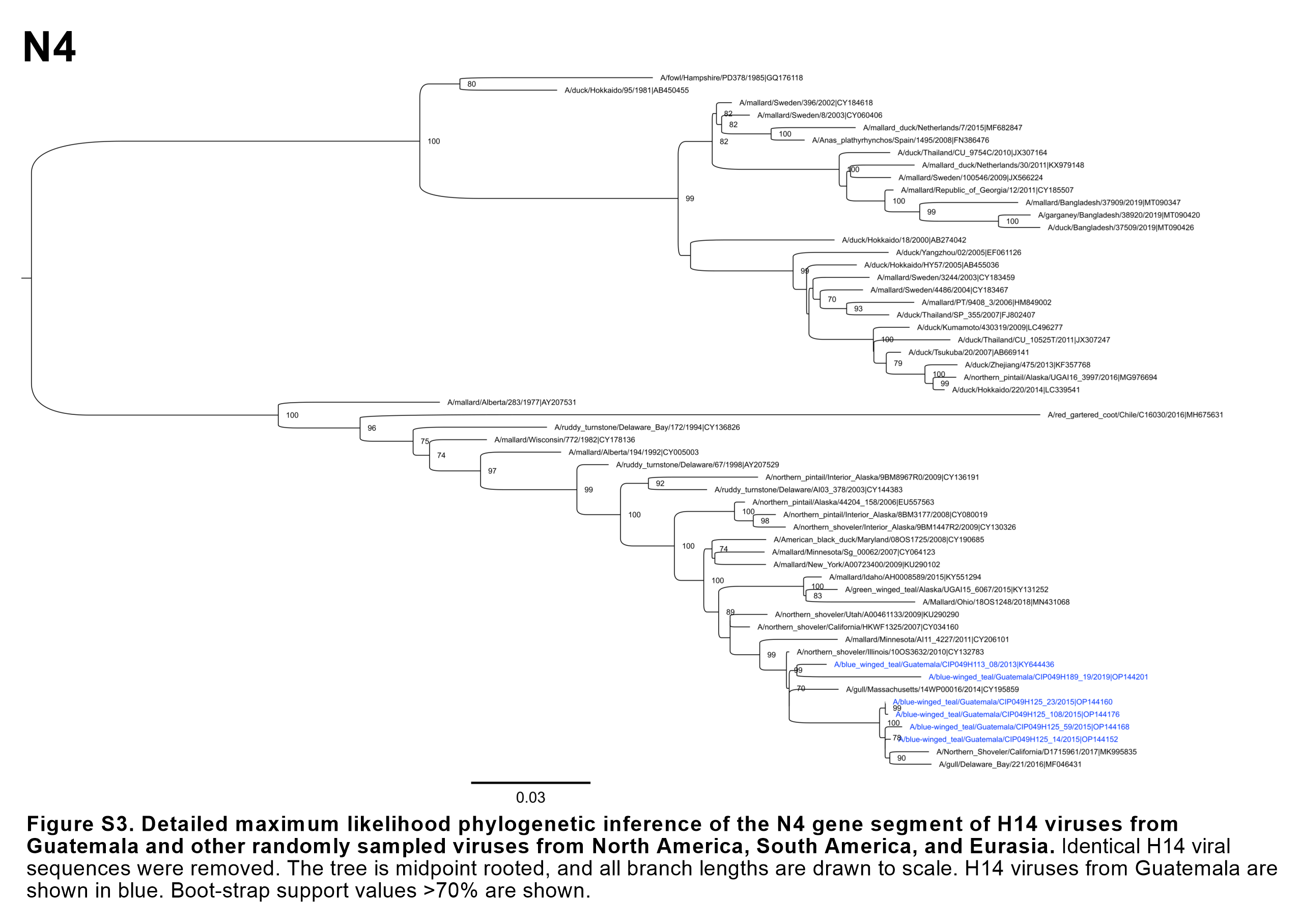

Supplement: Supplementary file 1 [file viruses-15-00483-s001.zip › Suppl_Figure S3_w_legend.tif]

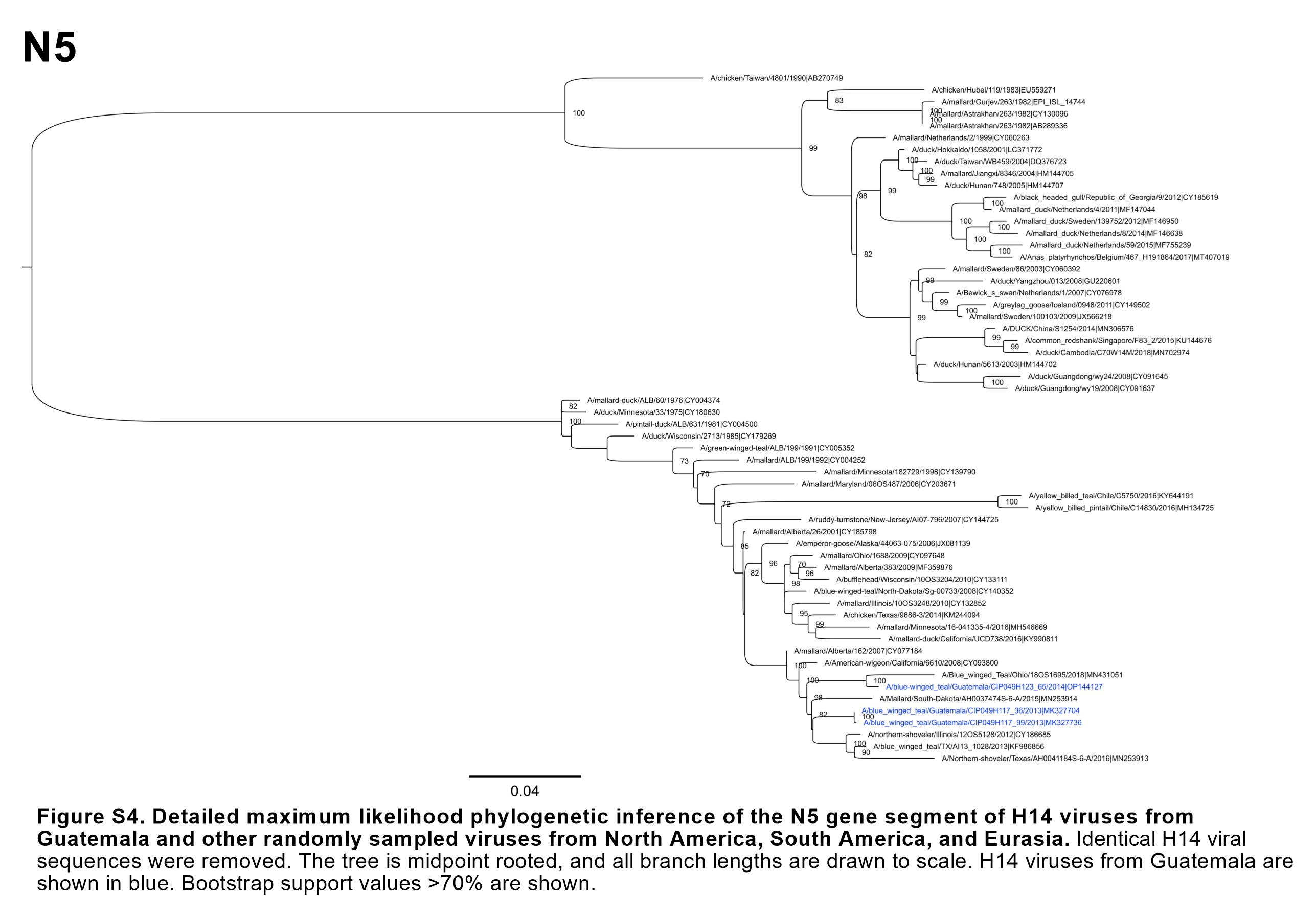

Supplement: Supplementary file 1 [file viruses-15-00483-s001.zip › Suppl_Figure S4_w_legend.tif]

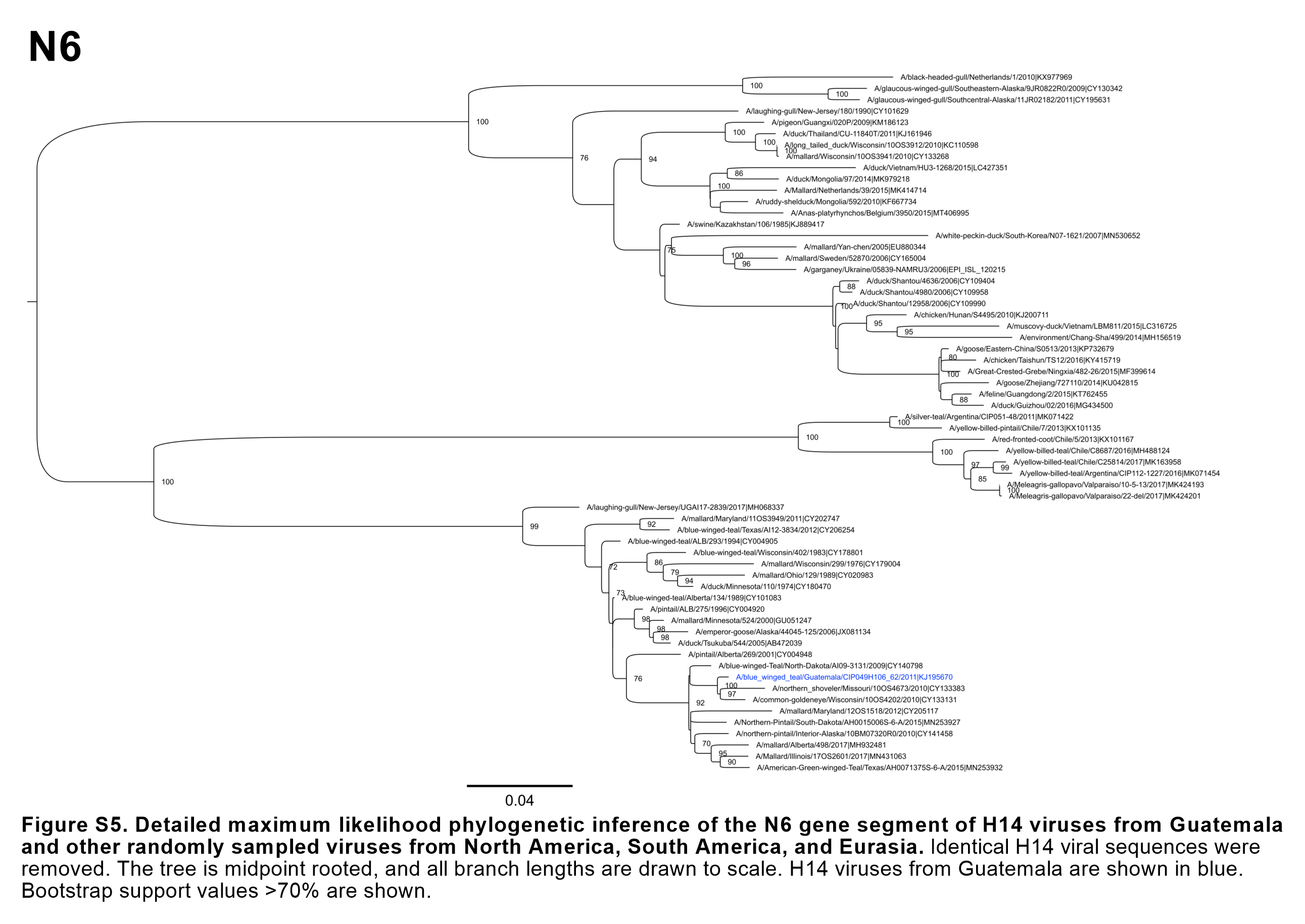

Supplement: Supplementary file 1 [file viruses-15-00483-s001.zip › Suppl_Figure S5_w_legend.tif]

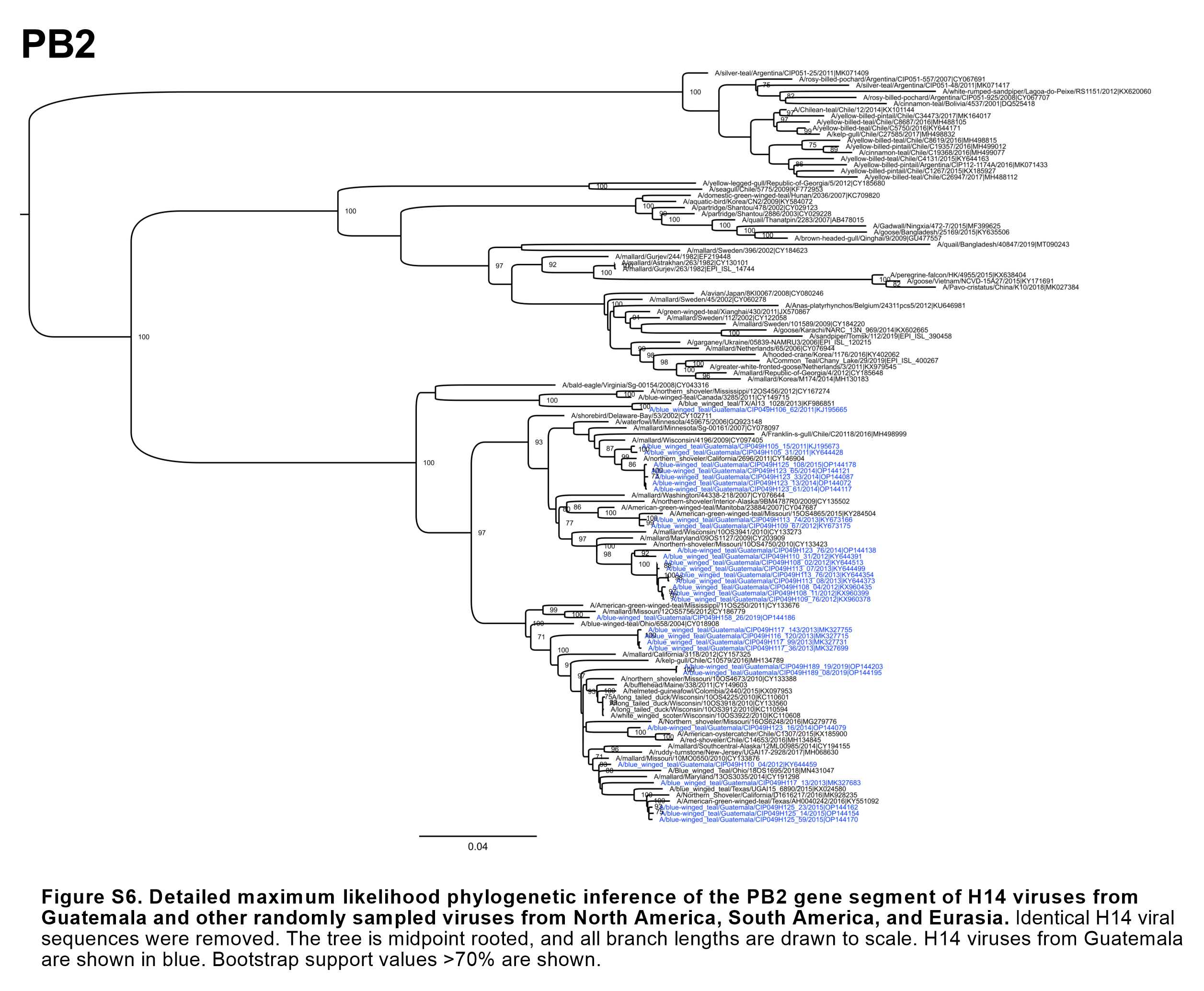

Supplement: Supplementary file 1 [file viruses-15-00483-s001.zip › Suppl_Figure S6_w_legend.tif]

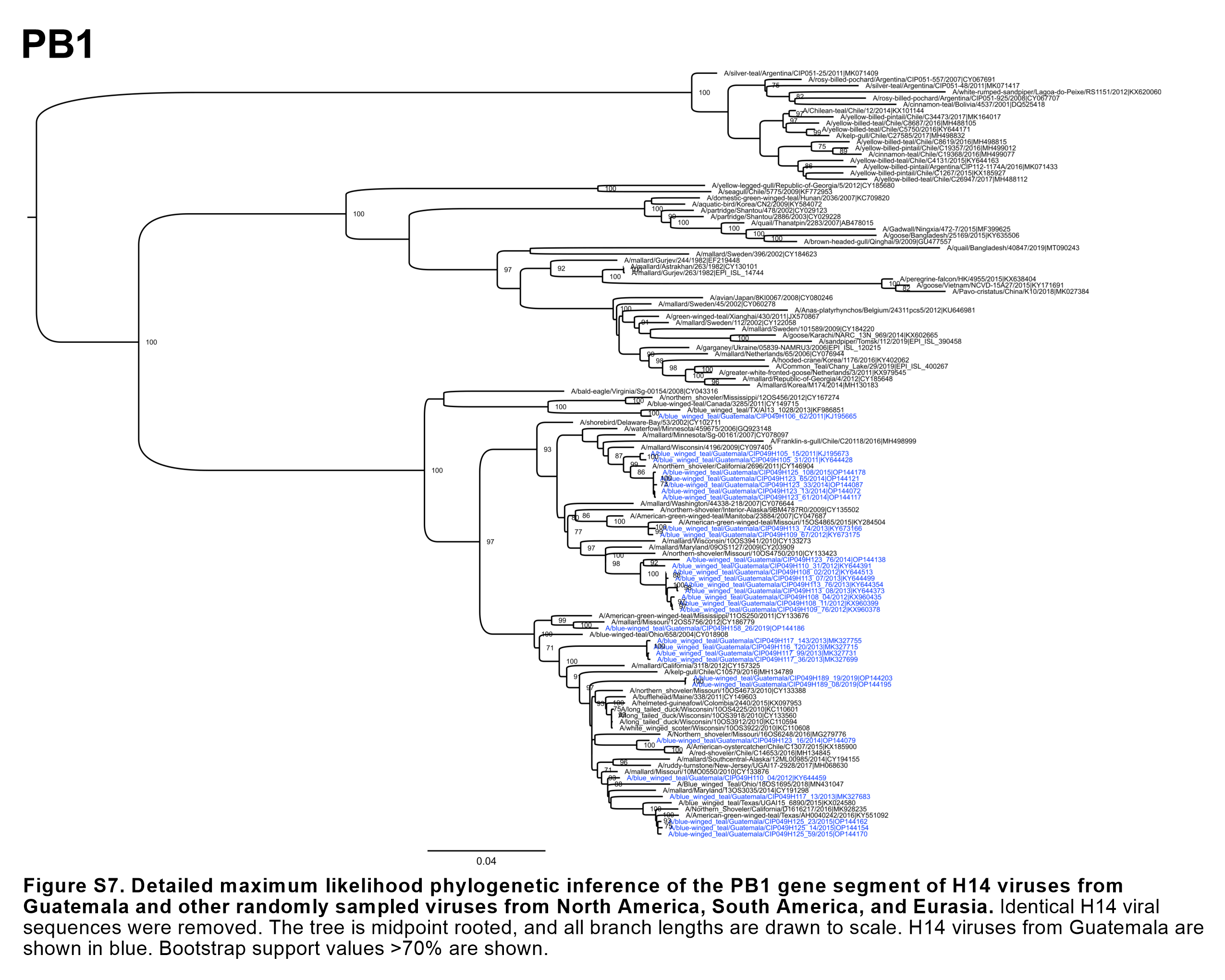

Supplement: Supplementary file 1 [file viruses-15-00483-s001.zip › Suppl_Figure S7_w_legend.tif]

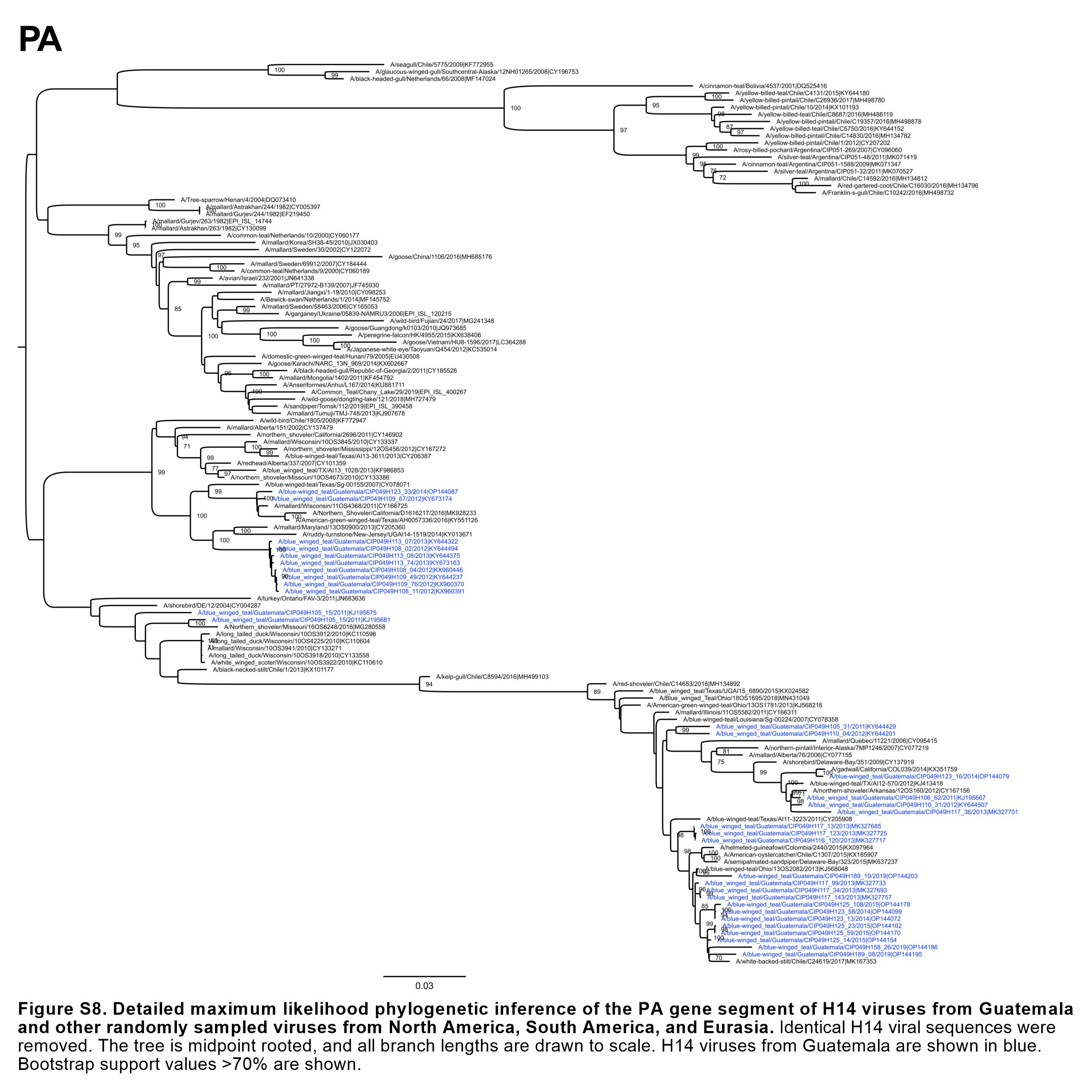

Supplement: Supplementary file 1 [file viruses-15-00483-s001.zip › Suppl_Figure S8_w_legend.tif]

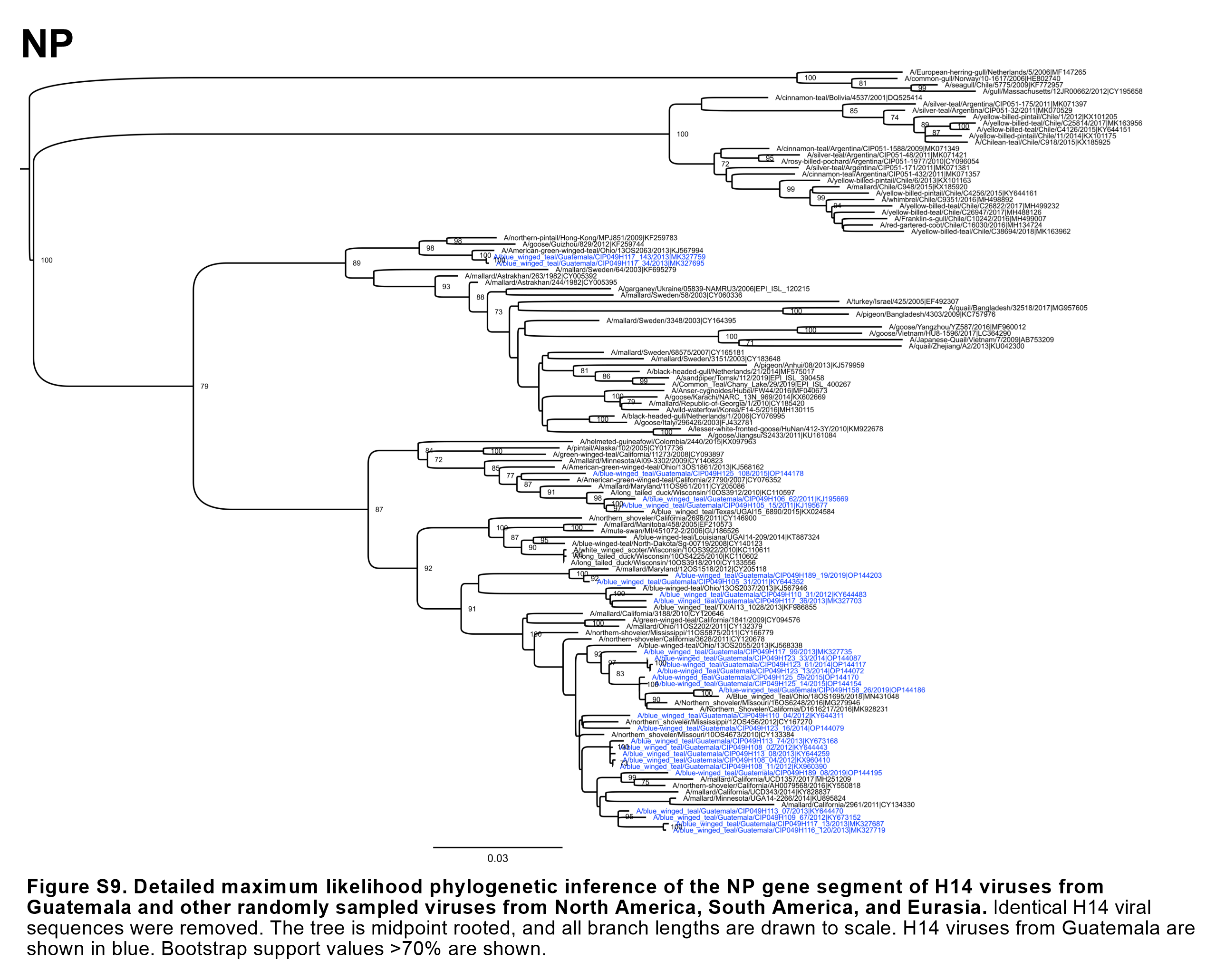

Supplement: Supplementary file 1 [file viruses-15-00483-s001.zip › Suppl_Figure S9_w_legend.tif]
