# Supplementary material for: Blue-Winged Teals in Guatemala and Their Potential Role in the Ecology of H14 Subtype Influenza a Viruses
Source: Viruses. 2023 Feb 9;15(2):483. doi: 10.3390/v15020483 (PMC9961055; doi:10.3390/v15020483)
Supplement: Supplementary file 1 [file Suppl_Table S1.pdf › Suppl_Table S1.pdf]

Suppl. Table S1. Detailed nucleotide pairwise identity of ORF sequences of concatenated gene (PB2, PB1, PA, HA, NP, M and NS) segments of full-length H14 viruses from Guatemala (n=40), North America (n=12), and Eurasia (n=4) during 1982-2019.

# virus	Strain	1-A	1-B	2	3	4	5	6	7	8	9	10-A	10-B	11	12	13	14	15	16	17	18	19	20	21	22	23	24	25	26	27	28	29	30	31	32	33	34	35	36	37	38	39	40	41	42	43	44	45	46	47	48	49	50	51	52	53	54	55	56
**1-A (variant #1)**	A/blue_winged_teal/Guatemala/CIP049H105_15/2011																																																										
**1-B (variant #2)**	A/blue_winged_teal/Guatemala/CIP049H105_15/2011	99.2																																																								
**2**	A/blue_winged_teal/Guatemala/CIP049H105_31/2011	96.2	96.2																																																						
**3**	A/blue_winged_teal/Guatemala/CIP049H106_62/2011	95.2	95.3	95.8																																																				
**4**	A/blue_winged_teal/Guatemala/CIP049H108_02/2012	95.3	95.1	95.1	93.8																																																		
**5**	A/blue_winged_teal/Guatemala/CIP049H108_04/2012	95.2	95.1	95.1	93.7	99.8																																																	
**6**	A/blue_winged_teal/Guatemala/CIP049H108_11/2012	95.2	95.1	95.1	93.7	99.8	99.9																																															
**7**	A/blue_winged_teal/Guatemala/CIP049H109_49/2012	95.2	95.1	95.1	93.7	99.8	99.9	99.9																																														
**8**	A/blue_winged_teal/Guatemala/CIP049H109_67/2012	95.4	95.4	95.2	94	96.9	96.8	96.8	96.8																																												
**9**	A/blue_winged_teal/Guatemala/CIP049H109_76/2012	95.2	95.1	95.2	93.8	99.6	99.7	99.8	99.7	96.8																																											
**10-A (variant #1)**	A/blue_winged_teal/Guatemala/CIP049H110_04/2012	94.3	94.4	96.6	95.4	95.2	95.1	95.1	95.1	95.3	95.2																																										
**10-B (variant #2)**	A/blue_winged_teal/Guatemala/CIP049H110_04/2012	94.3	94.3	96.5	95.3	95.4	95.3	95.3	95.3	95.3	95.1	99.8																																							
**11**	A/blue_winged_teal/Guatemala/CIP049H110_31/2012	94.6	94.7	96.4	96	96.1	96.1	96.1	96.1	95.2	96.2	96.1	95.9																																						
**12**	A/blue_winged_teal/Guatemala/CIP049H113_07/2013	95.2	95.1	95	93.7	99.6	99.4	99.4	99.4	97	99.2	95.1	95.3	96																																					
**13**	A/blue_winged_teal/Guatemala/CIP049H113_08/2013	95.2	95	95.2	93.8	99.3	99.3	99.3	99.3	96.7	99.1	94.9	95.1	95.9	99																																				
**14**	A/blue_winged_teal/Guatemala/CIP049H113_74/2013	95.1	95	95.2	93.7	98.5	98.4	98.4	98.4	97.8	98.3	95	95.2	95.3	98.2	98.5																																			
**15**	A/blue_winged_teal/Guatemala/CIP049H113_76/2013	95.2	95	95.2	93.8	99.3	99.3	99.3	99.3	96.6	99.1	95	95.1	95.9	99	100	98.5																																		
**16**	A/blue_winged_teal/Guatemala/CIP049H116_120/2013	93.6	93.6	95.8	94.6	94.1	94.1	94.1	94.1	94.4	94.2	96.7	96.7	94.9	94.2	94.3	94.1	94.3																																	
**17**	A/blue_winged_teal/Guatemala/CIP049H117_13/2013	94.2	94.2	96.4	94.9	94.8	94.8	94.8	94.7	94.9	94.9	97.8	97.7	95.5	94.9	94.9	94.5	94.9	97.8																																
**18**	A/blue_winged_teal/Guatemala/CIP049H117_34/2013	93.3	93.4	95.1	94.3	93	93	93	93	93.2	93.1	95.7	95.7	94.4	93	93.2	93	93.2	98.1	96																															
**19**	A/blue_winged_teal/Guatemala/CIP049H117_36/2013	93.7	93.8	95.3	95.3	93.8	93.8	93.8	93.8	93.9	93.9	95.8	95.7	96.3	93.7	94.1	93.8	94.1	97.8	95.7	97.3																														
**20**	A/blue_winged_teal/Guatemala/CIP049H117_38/2013	93.7	93.8	95.3	95.3	93.8	93.8	93.8	93.8	93.9	93.9	95.8	95.7	96.3	93.7	94.1	93.8	94.1	97.8	95.7	97.3	100																													
**21**	A/blue_winged_teal/Guatemala/CIP049H117_99/2013	93.6	93.6	95.9	94.6	94.1	94	94	94	94.3	94.1	96.8	96.7	95	94	94.3	94	94.3	99.1	97	98.3	98	98																												
**22**	A/blue_winged_teal/Guatemala/CIP049H117_123/2013	93.5	93.5	95.7	94.5	94	93.9	93.9	93.9	94.3	94.1	96.6	96.6	94.8	94.1	94.2	94	94.2	99.9	97.7	97.9	97.7	97.7	98.9																											
**23**	A/blue_winged_teal/Guatemala/CIP049H117_143/2013	93.3	93.4	95.1	94.3	93	92.9	92.9	92.9	93.1	93.1	95.7	95.6	94.3	93	93.2	93	93.2	98	96	99.9	97.2	97.2	98.2	97.9																										
**24**	A/blue-winged_teal/Guatemala/CIP049H123_13/2014	94.6	94.7	96.8	94.2	94.3	94.3	94.3	94.2	94.5	94.3	95.8	95.7	95.1	94.2	94.6	94.4	94.6	96.6	96.1	95.8	95.5	95.5	97.1	96.5	95.8																									
**25**	A/blue-winged_teal/Guatemala/CIP049H123_16/2014	93.8	93.8	95.4	95.3	94.4	94.3	94.3	94.3	94.4	94.4	96.9	96.8	95.7	94.3	94.6	94.2	94.5	96	96.7	95.2	96.1	96.1	96.1	95.9	95.1	95.4																								
**26**	A/blue-winged_teal/Guatemala/CIP049H123_33/2014	95.7	95.6	95.9	93.7	96.2	96.2	96.2	96.2	97.1	96.3	94.8	94.7	94.5	96.1	96.6	96.3	96.6	94.2	94.8	93.2	94	94	94.5	94.1	93.2	96.5	95.4																							
**27**	A/blue-winged_teal/Guatemala/CIP049H123_57/2014	94.6	94.7	96.9	94.2	94.3	94.3	94.3	94.3	94.5	94.4	95.8	95.7	95.1	94.2	94.6	94.4	94.6	96.7	96.1	95.8	95.5	95.5	97.1	96.5	95.8	100	95.4	96.5																						
**28**	A/blue-winged_teal/Guatemala/CIP049H123_58/2014	94.6	94.7	96.9	94.2	94.3	94.3	94.3	94.3	94.5	94.4	95.8	95.7	95.1	94.2	94.6	94.4	94.6	96.6	96.1	95.8	95.5	95.5	97.1	96.5	95.8	100	95.4	96.5	100																					
**29**	A/blue-winged_teal/Guatemala/CIP049H123_59/2014	95.3	95.3	97.5	94.9	94.9	94.9	94.9	94.8	95.1	94.9	96.4	96.3	95.7	94.8	95.1	94.9	95.1	96.2	96.7	95.4	95	95	96.6	96.1	95.3	98.9	96	97.1	98.9	98.9																				
**30**	A/blue-winged_teal/Guatemala/CIP049H123_61/2014	94.6	94.7	96.8	94.2	94.3	94.3	94.3	94.2	94.5	94.3	95.8	95.7	95.1	94.2	94.6	94.4	94.6	96.6	96	95.8	95.5	95.5	97.1	96.5	95.8	100	95.4	96.5	100	100	98.9																			
**31**	A/blue-winged_teal/Guatemala/CIP049H123_65/2014	94.6	94.7	96.9	94.2	94.3	94.3	94.3	94.3	94.5	94.4	95.8	95.7	95.1	94.2	94.6	94.4	94.6	96.7	96.1	95.8	95.5	95.5	97.1	96.5	95.8	100	95.4	96.5	100	99.9	98.9	99.9																		
**32**	A/blue-winged_teal/Guatemala/CIP049H123_75/2014	93.8	93.7	95.4	95.2	94.4	94.3	94.3	94.3	94.4	94.4	96.9	96.8	95.7	94.2	94.5	94.2	94.5	96	96.7	95.1	96.1	96.1	96.1	95.9	95.1	95.4	100	95.3	95.4	95.4	95.9	95.4	95.4																	
**33**	A/blue-winged_teal/Guatemala/CIP049H123_76/2014	93.8	93.8	96	94.1	95.1	95	95.1	95	94.5	95.1	95.8	95.7	95.9	95	95.3	94.4	95.3	96.6	96	95.8	95.4	95.4	97	96.5	95.7	98.7	95.3	95.3	98.7	98.7	97.7	98.7	98.7	95.3																
**34**	A/blue-winged_teal/Guatemala/CIP049H125_14/2015	93.8	93.8	96.1	94.7	94.6	94.5	94.5	94.5	94.5	94.6	97.5	97.4	95.2	94.5	94.8	94.5	94.8	96.8	97.6	96.1	95.7	95.7	97.3	96.7	96	96.5	96.8	95	96.5	96.5	97.1	96.5	96.6	96.7	96.5															
**35**	A/blue-winged_teal/Guatemala/CIP049H125_23/2015	92.3	92.3	93.6	93.1	91.6	91.5	91.5	91.5	91.6	91.6	94.5	94.5	92.7	91.5	91.8	91.4	91.8	95	94.7	94.9	94.3	94.3	95.3	94.9	94.9	95	93.8	91.9	95	95	94.1	95	95	93.8	95	95.9														
**36**	A/blue-winged_teal/Guatemala/CIP049H125_59/2015	93.8	93.8	96.1	94.7	94.5	94.4	94.5	94.4	94.5	94.6	97.5	97.4	95.1	94.4	94.8	94.4	94.8	96.9	97.8	96.2	95.6	95.6	97.3	96.7	96.1	96.5	96.8	95.1	96.5	96.5	97.4	96.5	96.5	96.7	96.5	99.2	96													
**37**	A/blue-winged_teal/Guatemala/CIP049H125_108/2015	95.3	95.3	96.6	94.7	93.6	93.6	93.6	93.5	93.8	93.7	95	94.9	94.8	93.5	93.8	93.6	93.8	96	95.5	96	95.2	95.2	96.2	95.9	96	98.5	94.5	95.3	98.5	98.5	97.6	98.5	98.5	94.5	97.2	95.7	96.1	95.8												
**38**	A/blue-winged_teal/Guatemala/CIP049H158_26/2019	93.6	93.6	95.5	94.4	94	93.9	93.9	93.9	94	94.1	96.3	96.2	94.7	93.9	94	93.9	94	95.7	96.2	94.9	94.5	94.5	95.9	95.5	94.9	95.2	95.1	94	95.3	95.3	95.9	95.2	95.3	95.1	95.2	96.6	93.3	96.7	94.5											
**39**	A/blue-winged_teal/Guatemala/CIP049H189_08/2019	93.4	93.4	95.4	94.2	93.9	93.8	93.8	93.8	94.1	94	96.6	96.6	94.5	93.9	93.8	93.8	93.8	95.9	96.7	95	94.6	94.6	96	95.8	94.9	95.3	95.6	93.8	95.3	95.3	95.8	95.3	95.3	95.5	95.2	96.7	93.8	96.8	94.7	96.6										
**40**	A/blue-winged_teal/Guatemala/CIP049H189_19/2019	93.3	93.4	95.9	94.3	93.5	93.4	93.4	93.4	93.4	93.5	96	95.9	94.5	93.3	93.4	93.4	93.4	95.2	96	95	94.5	94.5	95.4	95.1	95	94.6	95	93.3	94.6	94.6	95.2	94.5	94.6	95	94.5	95.9	93.6	96	94.6	95.9	97.7									
41	A/Blue_winged_Teal/Ohio/18OS1695/2018	93	93.1	95.1	93.9	93.7	93.6	93.6	93.6	93.7	93.8	96.5	96.4	94.4	93.7	93.7	93.6	93.7	95.3	96.2	94.4	94.4	94.4	95.4	95.2	94.4	94.5	95.3	93.7	94.6	94.6	95.1	94.5	94.6	95.3	94.5	96.6	93.1	96.4	93.9	96.6	96.2	95.6								
42	A/blue_winged_teal/Texas/UGAI15_6890/2015	94.5	94.6	95.6	95.3	93.4	93.4	93.4	93.4	93.6	93.5	96.3	96.3	94.5	93.4	93.4	93.2	93.4	95.3	96.2	95	94.7	94.7	95.2	95.1	94.9	94.2	95.3	93.3	94.2	94.2	94.8	94.2	94.2	95.2	94.2	96.3	94.6	96.4	94.8	95.5	95.8	95.8	96.1							
43	A/long_tailed_duck/Wisconsin/10OS3912/2010	94.4	94.4	92.2	92.5	92.3	92.2	92.2	92.2	92.2	92.2	93	93	91.8	92.2	92.3	92.1	92.3	92.5	92.7	92.5	92.7	92.7	92.6	92.4	92.5	91.7	92.7	92	91.7	91.7	91.5	91.7	91.7	92.7	91.7	92.4	94.9	92.5	92.4	91.8	92.1	92.1	91.8	93.1						
44	A/long_tailed_duck/Wisconsin/10OS3918/2010	93.7	93.7	92.3	91.7	92.5	92.5	92.5	92.5	92.5	92.5	93.3	93.2	92	92.5	92.5	92.4	92.5	92.7	92.8	92.5	92.9	92.9	92.8	92.6	92.5	91.9	93	92.2	91.9	91.9	91.7	91.9	91.9	93	91.9	92.6	94.5	92.7	91.9	91.9	92.3	92.2	92	92.5	99					
45	A/long_tailed_duck/Wisconsin/10OS4225/2010	94.9	95	93.5	93	93.8	93.7	93.7	93.7	93.7	93.7	94.4	94.5	93.2	93.7	93.8	93.6	93.8	94	94	93.8	94.2	94.2	94	93.8	93.7	93.1	94.2	93.4	93.1	93.1	92.9	93.1	93.1	94.2	93.1	93.8	92.8	93.9	93.2	93.1	93.5	93.5	93.2	93.8	97.1	98.1				
46	A/mallard/Wisconsin/10OS3941/2010	95.4	95.5	94.1	93	94.5	94.4	94.4	94.4	94.4	94.4	93.1	93.2	93.8	94.4	94.4	94.3	94.4	93.1	92.9	92.9	93.3	93.3	93.1	93	92.9	93.6	93	93.9	93.6	93.6	93.5	93.6	93.6	93	93.7	92.7	91.7	92.8	93.6	92.5	92.5	92.4	92.1	92.6	95.5	96.5	98.3			
47	A/northern_shoveler/California/2696/2011	94.1	94	93.6	91.7	93.7	93.6	93.6	93.6	93.7	93.7	92.2	92.1	92.4	93.6	93.8	93.9	93.8	91.8	91.9	91.3	91.9	91.9	91.8	91.7	91.3	93	91.6	94.2	93	93	93	92.9	93	91.6	92	91.6	93.1	91.7	92.9	91.4	91.3	91.2	91.1	91.5	94.1	94.6	93	93.5			
48	A/Northern_Shoveler/California/D1616217/2016	93.9	93.9	93.9	93.3	95.2	95.1	95.1	95.1	95.7	95.2	95.3	95.2	93.6	95.1	95.2	95	95.2	94	94.9	93.1	93.8	93.8	94.2	93.8	93.1	93.5	94.6	95.8	93.5	93.5	94	93.5	93.5	94.6	93.5	95.8	92.7	95.8	92.7	94.9	95	94.3	95	95	92.6	92.8	94	92.9	92.5							
49	A/northern_shoveler/Mississippi/12OS456/2012	92.2	92.1	91.9	92.5	93.1	93	93	93	93.2	93	92.3	92.3	91.5	93	93.2	93.1	93.2	92	91.8	90.9	91.6	91.6	92.1	91.9	90.8	91.8	91.8	92.9	91.8	91.8	91.8	91.8	91.8	91.8	91.8	91.7	92.8	91.8	91	91.5	91.1	90.6	91	90.9	93.6	93.8	92.1	92.1	94.5	92.4						
50	A/northern_shoveler/Missouri/10OS4673/2010	91.7	91.6	91.5	90.6	92.9	92.8	92.8	92.8	92.8	92.9	93	93	91	92.8	92.9	92.7	92.9	92.4	92.5	91.3	92	92	92.4	92.3	91.2	91.5	92.5	92.7	91.6	91.5	91.4	91.5	91.6	92.5	91.4	92.2	93.3	92.3	90.8	91.6	91.8	91.3	91.7	91.4	94.8	95.1	93.3	92	94.3	93	94.7					
51	A/Northern_shoveler/Missouri/16OS6248/2016	95	95.5	94	93.3	94.5	94.4	94.4	94.4	94.5	94.6	95.2	95.2	93.6	94.4	94.5	94.3	94.5	94.1	94.8	93.1	93.7	93.7	94.2	94	93.1	93.6	94.7	94.6	93.6	93.6	94.1	93.6	93.6	94.7	93.5	95.1	91.9	95.2	92.9	95	95	94.4	95.2	94.6	93.6	93.8	95	93.9	92	95.8	92	92.8				
52	A/white_winged_scoter/Wisconsin/10OS3922/2010	93.6	93.7	92.3	91.7	92.5	92.4	92.4	92.4	92.5	92.5	93.2	93.2	92	92.4	92.5	92.4	92.5	92.7	92.8	92.5	92.9	92.9	92.7	92.6	92.4	91.8	93	92.2	91.9	91.9	91.7	91.8	91.9	93	91.8	92.5	94.5	92.7	91.9	91.9	92.2	92.2	91.9	92.5	99	99.9	98	96.4	94.6	92.8	93.8	95	93.7			
53	A/Common_Teal/Chany_Lake/29/2019	89.4	89.3	88.7	88.4	89	89	89	89	89	89	88.6	88.6	88.4	89	88.9	88.9	88.9	88.3	88.5	89	88.4	88.4	88.4	88.2	89	88.4	88.2	88.8	88.4	88.4	88.5	88.4	88.4	88.2	88.4	88.4	87.2	88.4	88.6	88.3	88.3	88.4	87.9	88.5	87.9	87.8	89.2	89.1	87.5	88.6	87.3	87.4	88.8	87.7		
54	A/garganey/Ukraine/05839-NAMRU3/2006	88.7	88.6	88.2	87.9	88.5	88.5	88.5	88.5	88.2	88.5	87.8	87.8	88.1	88.5	88.6	88.5	88.6	87.7	87.8	88.2	87.7	87.7	87.7	87.6	88.2	87.7	87.6	88.1	87.7	87.7	87.9	87.7	87.7	87.6	87.8	87.7	89	87.7	87.9	87.6	87.3	87.3	87.1	87.7	89.8	89.8	88.5	88.6	89.5	87.9	89.5	89.3	88	89.7	92.3	
55	A/goose/Karachi/NARC_13N_969/2014	89.5	89.4	88.9	88.7	89.1	89	89.1	89	89	89.1	88.7	88.7	88.6	89	89.1	89	89.1	88.7	88.8	89.2	88.6	88.6	88.7	88.6	89.2	88.8	88.5	89.1	88.8	88.8	88.8	88.8	88.8	88.5	88.8	88.5	87.3	88.6	88.9	88.6	88.3	88.4	88.2	88.5	88	88	89.5	89.4	87.7	88.8	87.5	87.7	89	87.9	95.2	92.9	
56	A/mallard/Astrakhan/263/1982	89.5	89.5	88.7	88.7	89.1	89.1	89.1	89.1	89.1	89.1	88.7	88.7	88.4	89.1	89.1	89	89.1	88.5	88.6	89.1	88.5	88.5	88.5	88.3	89.1	88.5	88.5	89.1	88.5	88.5	88.6	88.5	88.5	88.5	88.5	88.5	87.1	88.6	88.6	88.6	88.3	88.2	88.1	88.6	88	88	89.4	89.5	87.6	89	87.6	87.5	89.3	88	93	92.7	93.5	
