# Supplementary material for: Blue-Winged Teals in Guatemala and Their Potential Role in the Ecology of H14 Subtype Influenza a Viruses
Source: Viruses. 2023 Feb 9;15(2):483. doi: 10.3390/v15020483 (PMC9961055; doi:10.3390/v15020483)
Supplement: Supplementary file 1 [file Suppl_Table S3.pdf › Suppl_Table S3.pdf]

Suppl. Table S3. Detailed nucleotide pairwise identity of ORF sequences of PB1 gene segment of full-length H14 viruses from Guatemala (n=40), North America (n=12), and Eurasia (n=4) during 1982-2019.

# virus	Strain	Reference ID	1	2	3	4	5	6	7	8	9	10	11	12	13	14	15	16	17	18	19	20	21	22	23	24	25	26	27	28	29	30	31	32	33	34	35	36	37	38	39	40	41	42	43	44	45	46	47	48	49	50	51	52	53	54	55	56
1	A/blue_winged_teal/Guatemala/CIP049H105_15-A/2011	KJ195674																																																						
2	A/blue_winged_teal/Guatemala/CIP049H105_31/2011	KY644306	99.3																																																					
3	A/blue_winged_teal/Guatemala/CIP049H106_62/2011	KJ195666	99.5	99.4																																																				
4	A/blue_winged_teal/Guatemala/CIP049H108_02/2012	KY644290	98.6	98.8	98.7																																																			
5	A/blue_winged_teal/Guatemala/CIP049H108_04/2012	KX986864	98.6	98.9	98.7	99.9																																																		
6	A/blue_winged_teal/Guatemala/CIP049H108_11/2012	KX986863	98.6	98.9	98.7	99.9	100																																																	
7	A/blue_winged_teal/Guatemala/CIP049H109_49/2012	KY644291	98.5	98.8	98.6	99.8	99.9	99.9																																																
8	A/blue_winged_teal/Guatemala/CIP049H109_67/2012	KY673169	98.8	99	98.9	98.4	98.4	98.4	98.4																																															
9	A/blue_winged_teal/Guatemala/CIP049H109_76/2012	KX986868	98.6	98.9	98.7	99.9	100	100	99.9	98.4																																													
10	A/blue_winged_teal/Guatemala/CIP049H110_04/2012	KY644275	98.9	99.1	99	98.4	98.5	98.5	98.4	98.5	98.5																																											
11	A/blue_winged_teal/Guatemala/CIP049H110_31/2012	KY644393	98.6	98.9	98.7	99.9	100	100	99.9	98.4	100	98.5																																									
12	A/blue_winged_teal/Guatemala/CIP049H113_07/2013	KY644309	98.5	98.7	98.6	99.9	99.8	99.8	99.7	98.3	99.8	98.3	99.8																																								
13	A/blue_winged_teal/Guatemala/CIP049H113_08/2013	KY644478	98.6	98.8	98.8	99.7	99.8	99.8	99.7	98.4	99.8	98.4	99.8	99.6																																							
14	A/blue_winged_teal/Guatemala/CIP049H113_74/2013	KY673165	98.5	98.8	98.7	99.7	99.7	99.7	99.6	98.3	99.7	98.4	99.7	99.6	100																																					
15	A/blue_winged_teal/Guatemala/CIP049H113_76/2013	KY644270	98.6	98.8	98.8	99.7	99.8	99.8	99.7	98.4	99.8	98.4	99.8	99.6	100	100																																				
16	A/blue_winged_teal/Guatemala/CIP049H116_120/2013	MK327716	96.1	96.1	96	95.8	95.9	95.9	95.9	95.9	95.9	95.7	95.9	95.7	95.9	95.9	95.9																																			
17	A/blue_winged_teal/Guatemala/CIP049H117_13/2013	MK327684	98.8	99	98.9	98.3	98.3	98.3	98.2	98.7	98.3	98.6	98.3	98.2	98.3	98.3	98.3	95.6																																		
18	A/blue_winged_teal/Guatemala/CIP049H117_34/2013	MK327692	96.1	96.1	96	95.8	95.9	95.9	95.9	95.9	95.9	95.7	95.9	95.7	95.9	95.9	95.9	100	95.6																																	
19	A/blue_winged_teal/Guatemala/CIP049H117_36/2013	MK327700	96	96.1	96	95.9	95.9	95.9	95.9	95.8	95.9	95.6	95.9	95.8	96	95.9	96	100	95.5	99.9																																
20	A/blue_winged_teal/Guatemala/CIP049H117_38/2013	MK326654	96	96.1	96	95.9	95.9	95.9	95.9	95.8	95.9	95.6	95.9	95.8	96	95.9	96	100	95.5	99.9	100																															
21	A/blue_winged_teal/Guatemala/CIP049H117_99/2013	MK327732	96	96.1	96	95.8	95.8	95.8	95.8	95.8	95.8	95.6	95.8	95.7	95.9	95.8	95.9	100	95.5	99.9	99.9	99.9																														
22	A/blue_winged_teal/Guatemala/CIP049H117_123/2013	MK327724	95.5	95.6	95.4	95.2	95.3	95.3	95.3	95.3	95.3	95.1	95.3	95.2	95.3	95.3	95.3	99.3	95	99.3	99.3	99.3	99.3																													
23	A/blue_winged_teal/Guatemala/CIP049H117_143/2013	MK327756	96	96.1	96	95.8	95.8	95.8	95.8	95.8	95.8	95.6	95.8	95.7	95.9	95.8	95.9	100	95.5	99.9	99.9	99.9	99.9	99.3																												
24	A/blue-winged_teal/Guatemala/CIP049H123_13/2014	OP144066	95.2	95.3	95.1	95	95.1	95.1	95	95	95.1	95.1	95.1	94.9	95.1	95.1	95.1	97.8	94.9	97.8	97.8	97.8	97.8	97.2	97.8																											
25	A/blue-winged_teal/Guatemala/CIP049H123_16/2014	OP144074	98.3	98.5	98.3	97.7	97.8	97.8	97.7	97.9	97.8	98	97.8	97.6	97.7	97.7	97.7	95.4	98	95.4	95.3	95.3	95.3	94.8	95.3	94.6																										
26	A/blue-winged_teal/Guatemala/CIP049H123_33/2014	OP144082	98.3	98.5	98.3	97.7	97.8	97.8	97.7	97.9	97.8	98	97.8	97.6	97.7	97.7	97.7	95.4	98	95.4	95.3	95.3	95.3	94.8	95.3	94.6	100																									
27	A/blue-winged_teal/Guatemala/CIP049H123_57/2014	OP144090	95.2	95.4	95.2	95.1	95.1	95.1	95	95	95.1	95.1	95.1	95	95.2	95.1	95.2	97.9	94.9	97.9	97.8	97.8	97.8	97.2	97.8	100	94.6	94.6																								
28	A/blue-winged_teal/Guatemala/CIP049H123_58/2014	OP144098	95.3	95.4	95.2	95.1	95.2	95.2	95.1	95.1	95.2	95.2	95.2	95	95.2	95.2	95.2	97.8	94.9	97.8	97.8	97.8	97.8	97.2	97.8	99.9	94.7	94.7	100																							
29	A/blue-winged_teal/Guatemala/CIP049H123_59/2014	OP144106	98.5	98.7	98.5	98	98.1	98.1	98	98.2	98.1	98.2	98.1	97.9	98	98	98	95.6	98.2	95.6	95.6	95.6	95.6	95	95.6	94.9	97.7	97.7	94.9	94.9																											
30	A/blue-winged_teal/Guatemala/CIP049H123_61/2014	OP144114	95.2	95.4	95.2	95.1	95.1	95.1	95	95	95.1	95.1	95.1	95	95.2	95.1	95.2	97.9	94.9	97.9	97.8	97.8	97.8	97.2	97.8	100	94.6	94.6	100	100	94.9																										
31	A/blue-winged_teal/Guatemala/CIP049H123_65/2014	OP144122	95.2	95.4	95.2	95.1	95.1	95.1	95	95	95.1	95.1	95.1	95	95.2	95.1	95.2	97.9	94.9	97.9	97.8	97.8	97.8	97.2	97.8	100	94.6	94.6	100	100	94.9																										
32	A/blue-winged_teal/Guatemala/CIP049H123_75/2014	OP144131	98.3	98.4	98.3	97.7	97.7	97.7	97.6	97.9	97.7	97.9	97.7	97.6	97.7	97.6	97.7	95.3	98	95.3	95.3	95.3	95.3	94.8	95.3	94.6	100	100	94.7	94.7	97.6	94.7	94.7																								
33	A/blue-winged_teal/Guatemala/CIP049H123_76/2014	OP144139	95.2	95.4	95.2	95.1	95.1	95.1	95	95	95.1	95.1	95.1	95	95.2	95.1	95.2	97.9	94.9	97.9	97.8	97.8	97.8	97.2	97.8	100	94.6	94.6	100	100	94.9	100	100	94.7																							
34	A/blue-winged_teal/Guatemala/CIP049H125_14/2015	OP144156	98.2	98.4	98.3	98.4	98.4	98.4	98.3	98	98.4	98	98.4	98.3	98.4	98.3	98.4	95.4	97.9	95.4	95.5	95.5	95.4	94.9	95.4	94.6	97.3	97.3	94.7	94.7	97.9	94.7	94.7	97.3	94.7																						
35	A/blue-winged_teal/Guatemala/CIP049H125_23/2015	OP144164	95.4	95.6	95.3	95.2	95.3	95.3	95.2	95.2	95.3	95.2	95.3	95.2	95.3	95.3	95.3	98	95	98	98	98	98	97.4	98	99.4	94.8	94.8	99.4	99.4	94.9	99.4	99.4	94.9	99.4	94.9																					
36	A/blue-winged_teal/Guatemala/CIP049H125_59/2015	OP144172	98.5	98.7	98.5	98.1	98.2	98.2	98.1	98.2	98.2	98.2	98.2	98	98.1	98.1	98.1	95.6	98.2	95.6	95.6	95.6	95.6	95	95.6	94.8	97.7	97.7	94.8	94.9	99.5	94.8	94.8	97.6	94.8	97.7	94.9																				
37	A/blue-winged_teal/Guatemala/CIP049H125_108/2015	OP144148	95.4	95.5	95.3	95.2	95.2	95.2	95.2	95.2	95.2	95.2	95.2	95.1	95.3	95.2	95.3	98	94.9	98	97.9	97.9	97.9	97.3	97.9	99.3	94.8	94.8	99.4	99.3	94.9	99.4	99.4	94.8	99.4	94.8	100	94.9																			
38	A/blue-winged_teal/Guatemala/CIP049H158_26/2019	OP144180	97.7	97.8	97.8	97.3	97.3	97.3	97.2	97.3	97.3	97.5	97.3	97.2	97.4	97.3	97.4	94.9	97.4	94.9	94.8	94.8	94.8	94.3	94.8	93.9	96.7	96.7	93.9	94	97.1	93.9	93.9	96.7	93.9	97.1	94.1	97.1	94.1																		
39	A/blue-winged_teal/Guatemala/CIP049H189_08/2019	OP144188	98.3	98.5	98.3	97.7	97.8	97.8	97.7	97.9	97.8	98.2	97.8	97.6	97.7	97.7	97.7	95.3	98.2	95.3	95.2	95.2	95.2	94.7	95.2	94.8	97.4	97.4	94.8	94.9	97.8	94.8	94.8	97.4	94.8	97.4	95	97.8	95	96.9																	
40	A/blue-winged_teal/Guatemala/CIP049H189_19/2019	OP144197	97.8	98.1	97.9	97.3	97.4	97.4	97.3	97.4	97.4	97.7	97.4	97.2	97.3	97.3	97.3	94.9	97.6	94.9	94.9	94.9	94.9	94.3	94.9	94.1	96.9	96.9	94.1	94.1	97.1	94.1	94.1	97	94.1	97.1	94.3	97.1	94.2	96.4	97.2																
41	A/Blue_winged_Teal/Ohio/18OS1695/2018	MN431046	97.1	97.3	97.1	97.2	97.3	97.3	97.2	96.8	97.3	97	97.3	97.1	97.2	97.2	97.2	94.6	96.7	94.6	94.6	94.6	94.5	94.1	94.5	93.8	96.4	96.4	93.9	93.9	96.7	93.9	93.9	96.4	93.9	98.4	94.1	96.7	94	96.2	96.2	96															
42	A/blue_winged_teal/Texas/UGAI15_6890/2015	KX024581	98	98.2	98	97.4	97.4	97.4	97.5	98.6	97.4	97.6	97.4	97.3	97.4	97.4	97.4	95.2	97.8	95.2	95.2	95.2	95.1	94.5	95.1	94.2	97.2	97.2	94.2	94.3	97.4	94.2	94.2	97.1	94.2	97.1	94.4	97.3	94.5	96.5	97.1	96.6	96														
43	A/long_tailed_duck/Wisconsin/10OS3912/2010	KC110595	96.9	97	96.8	96.5	96.6	96.6	96.5	96.7	96.6	96.5	96.6	96.5	96.6	96.6	96.6	97.4	96.4	97.4	97.4	97.4	97.4	96.8	97.4	97	96.3	96.3	97	97.1	96.2	97	97	96.3	97	96	97.1	96.2	97	95.4	96.1	95.7	95.1	95.8													
44	A/long_tailed_duck/Wisconsin/10OS3918/2010	CY133559	96.8	97	96.7	96.5	96.5	96.5	96.4	96.6	96.5	96.5	96.5	96.5	96.6	96.5	96.6	97.4	96.3	97.4	97.4	97.4	97.4	96.8	97.4	96.9	96.2	96.2	97	97	96.2	97	97	96.3	97	95.9	97	96.2	97	95.3	96.1	95.6	95	95.8	100												
45	A/long_tailed_duck/Wisconsin/10OS4225/2010	KC110603	96.8	96.9	96.7	96.4	96.5	96.5	96.4	96.6	96.5	96.4	96.5	96.4	96.5	96.5	96.5	97.4	96.3	97.4	97.4	97.4	97.4	96.8	97.4	96.9	96.2	96.2	96.9	97	96.1	96.9	96.9	96.2	96.9	95.9	97	96.1	96.9	95.3	96.1	95.7	95	95.8	99.9	99.9											
46	A/mallard/Wisconsin/10OS3941/2010	CY133272	96.8	97	96.7	96.5	96.5	96.5	96.4	96.6	96.5	96.5	96.5	96.5	96.6	96.5	96.6	97.5	96.3	97.5	97.4	97.4	97.4	96.9	97.4	96.9	96.2	96.2	97	97	96.2	97	97	96.3	97	95.9	97	96.2	97	95.3	96.2	95.7	95	95.7	100	99.9	100										
47	A/northern_shoveler/California/2696/2011	CY146903	95.6	95.8	95.6	95.4	95.4	95.4	95.3	95.4	95.4	95.6	95.4	95.3	95.5	95.4	95.5	95.4	95.5	95.4	95.4	95.4	95.5	94.8	95.4	95	95.1	95.1	95	95.1	95.3	95	95	95	95	95	95	95.2	95	94.3	95.2	94.7	94.2	94.8	96.3	96.3	96.2	96.3									
48	A/Northern_Shoveler/California/D1616217/2016	MK928234	97.7	97.8	97.7	97.3	97.4	97.4	97.4	97.4	97.4	97.5	97.4	97.2	97.3	97.3	97.3	94.8	97.5	94.8	94.7	94.7	94.7	94.1	94.7	94.2	96.7	96.7	94.3	94.3	97	94.3	94.3	96.8	94.3	96.7	94.5	97	94.5	96.2	98	96.7	95.5	96.6	95.5	95.5	95.4	95.5	94.9								
49	A/northern_shoveler/Mississippi/12OS456/2012	CY167273	95.6	95.7	95.4	95.3	95.3	95.3	95.2	95.5	95.3	95.4	95.3	95.2	95.4	95.3	95.4	95.7	95.2	95.7	95.6	95.6	95.7	95.1	95.6	95.1	95.1	95.1	95.1	95.2	95.2	95.1	95.1	95.2	95.1	94.7	95.2	95.1	95.2	94.3	94.6	94.5	93.8	94.7	96.4	96.4	96.4	96.5	95.1	94.3							
50	A/northern_shoveler/Missouri/10OS4673/2010	CY133387	92.5	92.9	92.5	92.3	92.3	92.3	92.2	92.7	92.3	92.6	92.3	92.3	92.3	92.2	92.3	93.4	92.6	93.4	93.4	93.4	93.4	92.9	93.4	93.1	92.6	92.6	93.1	93.1	92.3	93.1	93.1	92.5	93.1	92	93.2	92.2	93.2	91.6	92.3	92.1	91.2	92.1	93.6	93.7	93.5	93.6	92.2	91.9	93.1						
51	A/Northern_shoveler/Missouri/16OS6248/2016	MG280367	97.7	97.9	97.8	97.4	97.4	97.4	97.4	97.4	97.4	97.6	97.4	97.3	97.4	97.3	97.4	94.9	97.5	94.9	94.9	94.9	94.8	94.3	94.8	94.2	97.1	97.1	94.2	94.3	97.1	94.2	94.2	97.1	94.2	97	94.5	97.1	94.5	96.3	97.4	96.8	95.8	96.8	95.5	95.5	95.5	95.6	94.5	96.6	94.4	91.9					
52	A/white_winged_scoter/Wisconsin/10OS3922/2010	KC110609	96.8	97	96.7	96.5	96.5	96.5	96.4	96.7	96.5	96.5	96.5	96.5	96.6	96.5	96.6	97.4	96.4	97.4	97.4	97.4	97.4	96.8	97.4	96.9	96.2	96.2	97	97	96.2	97	97	96.3	97	95.9	97	96.2	97	95.3	96.1	95.6	95	95.7	99.9	99.8	99.8	99.8	96.3	95.5	96.4	93.6	95.5				
53	A/Common_Teal/Chany_Lake/29/2019	EPI_ISL_400267	88.9	88.9	88.7	88.5	88.5	88.5	88.4	88.7	88.5	88.7	88.5	88.4	88.3	88.3	88.3	88.5	88.8	88.5	88.5	88.5	88.6	88	88.6	88.2	88.4	88.4	88.2	88.2	88.8	88.2	88.2	88.4	88.2	88.6	88.3	88.7	88.3	88.5	88.8	88.6	87.6	88.4	88.7	88.8	88.6	88.7	87.7	88.3	88.3	87.9	88.8	88.7			
54	A/garganey/Ukraine/05839-NAMRU3/2006	EPI_ISL_120215	89.3	89.4	89.4	89.3	89.2	89.2	89.2	89.1	89.2	89	89.2	89.2	89.2	89.2	89.2	89	89.4	89	88.9	88.9	89	88.4	89	88.6	89	89	88.7	88.6	89.3	88.7	88.7	89.1	88.7	88.9	88.6	89.2	88.7	89	88.9	88.6	88	88.9	88.8	88.8	88.7	88.7	88.1	88.8	88.8	88	89.1	88.7	92.6		
55	A/goose/Karachi/NARC_13N_969/2014	KX602666	88.5	88.7	88.5	88.1	88.2	88.2	88.1	88.5	88.2	88.3	88.2	88	88.1	88.2	88.1	88.4	88.6	88.4	88.4	88.4	88.5	87.9	88.5	88.3	88.3	88.3	88.4	88.3	88.4	88.4	88.4	88.3	88.4	88.2	88.5	88.3	88.5	88.3	88.1	88.3	87.6	88.1	88.7	88.8	88.6	88.7	87.8	88	88.3	87.8	88.4	88.7	96.7	93	
56	A/mallard/Astrakhan/263/1982	CY130100	89.7	89.8	89.6	89.5	89.4	89.4	89.4	89.6	89.4	89.5	89.4	89.4	89.3	89.3	89.3	89.4	89.7	89.4	89.3	89.3	89.4	88.8	89.4	89	89.4	89.4	89.1	89	89.5	89.1	89.1	89.5	89.1	89.4	89.3	89.5	89.3	89.5	89.3	89.2	88.3	89.4	89.6	89.7	89.5	89.6	89.1	89.3	89.3	88.4	89.7	89.6	94.5	95.7	94.5
