# Supplementary material for: Blue-Winged Teals in Guatemala and Their Potential Role in the Ecology of H14 Subtype Influenza a Viruses
Source: Viruses. 2023 Feb 9;15(2):483. doi: 10.3390/v15020483 (PMC9961055; doi:10.3390/v15020483)
Supplement: Supplementary file 1 [file Suppl_Table S5.pdf › Suppl_Table S5.pdf]

Suppl. Table S5. Detailed nucleotide pairwise identity of ORF sequences of HA H14 gene segment of full-length H14 viruses from Guatemala (n=40), North America (n=12), and Eurasia (n=4) during 1982-2019.

# virus	Strain	Reference ID	1	2	3	4	5	6	7	8	9	10	11	12	13	14	15	16	17	18	19	20	21	22	23	24	25	26	27	28	29	30	31	32	33	34	35	36	37	38	39	40	41	42	43	44	45	46	47	48	49	50	51	52	53	54	55	56
1	A/blue_winged_teal/Guatemala/CIP049H105_15/2011	KJ195676																																																						
2	A/blue_winged_teal/Guatemala/CIP049H105_31/2011	KY644423	99.2																																																					
3	A/blue_winged_teal/Guatemala/CIP049H106_62/2011	KJ195668	99.5	99.4																																																				
4	A/blue_winged_teal/Guatemala/CIP049H108_02/2012	KY644472	98.2	98.7	98.4																																																			
5	A/blue_winged_teal/Guatemala/CIP049H108_04/2012	KX960412	98.1	98.5	98.2	99.8																																																		
6	A/blue_winged_teal/Guatemala/CIP049H108_11/2012	KX960458	98	98.5	98.2	99.8	99.9																																																	
7	A/blue_winged_teal/Guatemala/CIP049H109_49/2012	KY644351	98.1	98.4	98.1	99.7	99.9	99.8																																																
8	A/blue_winged_teal/Guatemala/CIP049H109_67/2012	KY673170	98.1	98.6	98.3	99.9	99.7	99.6	99.6																																															
9	A/blue_winged_teal/Guatemala/CIP049H109_76/2012	KX960451	98.1	98.5	98.2	99.8	100	99.9	99.9	99.7																																														
10	A/blue_winged_teal/Guatemala/CIP049H110_04/2012	KY644317	98.1	98.5	98.2	99.8	99.6	99.6	99.5	99.9	99.6																																												
11	A/blue_winged_teal/Guatemala/CIP049H110_31/2012	KY644243	98.1	98.5	98.2	99.8	99.6	99.6	99.5	99.7	99.6	99.6																																											
12	A/blue_winged_teal/Guatemala/CIP049H113_07/2013	KY644236	97.9	98.4	98.1	99.7	99.5	99.5	99.4	99.6	99.5	99.5	99.5																																										
13	A/blue_winged_teal/Guatemala/CIP049H113_08/2013	KY644232	98.2	98.7	98.4	98.5	98.4	98.3	98.2	98.4	98.4	98.4	98.4	98.2																																									
14	A/blue_winged_teal/Guatemala/CIP049H113_74/2013	KY673155	98.1	98.6	98.3	99.9	99.7	99.6	99.6	99.8	99.7	99.7	99.7	99.7	98.4																																								
15	A/blue_winged_teal/Guatemala/CIP049H113_76/2013	KY644258	98.2	98.7	98.4	98.5	98.4	98.3	98.2	98.4	98.4	98.4	98.4	98.2	100	98.4																																						
16	A/blue_winged_teal/Guatemala/CIP049H116_120/2013	MK327718	97.7	98.2	97.8	98	97.8	97.8	97.7	97.9	97.8	97.8	97.8	97.7	99.4	97.9	99.4																																					
17	A/blue_winged_teal/Guatemala/CIP049H117_13/2013	MK327686	97.7	98.2	97.8	98	97.8	97.8	97.7	97.9	97.8	97.8	97.8	97.7	99.4	97.9	99.4	100																																				
18	A/blue_winged_teal/Guatemala/CIP049H117_34/2013	MK327694	97.6	98.1	97.8	97.9	97.8	97.7	97.7	97.8	97.8	97.8	97.8	97.8	99.3	97.8	99.3	99.9	99.9																																			
19	A/blue_winged_teal/Guatemala/CIP049H117_36/2013	MK327702	97.7	98.2	97.8	98	97.8	97.8	97.7	97.9	97.8	97.8	97.8	97.7	99.4	97.9	99.4	100	100	99.9																																		
20	A/blue_winged_teal/Guatemala/CIP049H117_38/2013	MK326656	97.7	98.2	97.8	98	97.8	97.8	97.7	97.9	97.8	97.8	97.8	97.7	99.4	97.9	99.4	100	100	99.9	100																																	
21	A/blue_winged_teal/Guatemala/CIP049H117_99/2013	MK327734	97.7	98.2	97.8	98	97.8	97.8	97.7	97.9	97.8	97.8	97.8	97.7	99.4	97.9	99.4	100	100	99.9	100	100																																
22	A/blue_winged_teal/Guatemala/CIP049H117_123/2013	MK327726	97.7	98.2	97.8	98	97.8	97.8	97.7	97.9	97.8	97.8	97.8	97.7	99.4	97.9	99.4	100	100	99.9	100	100	100																															
23	A/blue_winged_teal/Guatemala/CIP049H117_143/2013	MK327758	97.6	98.1	97.8	97.9	97.8	97.7	97.7	97.8	97.8	97.8	97.8	97.7	99.3	97.8	99.3	99.9	99.9	99.9	99.9	99.9	99.9	99.9																														
24	A/blue-winged_teal/Guatemala/CIP049H123_13/2014	OP144068	96.8	97.4	97	97.1	96.9	96.8	96.8	97	96.9	96.9	96.9	96.9	98.4	97	98.4	99.1	99.1	99.1	99.1	99.1	99.1	99.1	99																														
25	A/blue-winged_teal/Guatemala/CIP049H123_16/2014	OP144076	96.7	97.2	96.8	96.9	96.7	96.7	96.6	96.8	96.7	96.7	96.7	96.7	98.2	96.8	98.2	98.9	98.9	98.9	98.9	98.9	98.9	98.9	98.8	99.7																															
26	A/blue-winged_teal/Guatemala/CIP049H123_33/2014	OP144084	96.8	97.4	97	97.1	96.9	96.8	96.8	97	96.9	96.9	96.9	96.9	98.4	97	98.4	99.1	99.1	99.1	99.1	99.1	99.1	99.1	99	100	99.7																														
27	A/blue-winged_teal/Guatemala/CIP049H123_57/2014	OP144092	96.8	97.4	97	97.1	96.9	96.8	96.8	97	96.9	96.9	96.9	96.9	98.4	97	98.4	99.1	99.1	99.1	99.1	99.1	99.1	99.1	99	100	99.7	100																													
28	A/blue-winged_teal/Guatemala/CIP049H123_58/2014	OP144100	96.8	97.4	97	97.1	96.9	96.8	96.8	97	96.9	96.9	96.9	96.9	98.4	97	98.4	99.1	99.1	99.1	99.1	99.1	99.1	99.1	99	100	99.7	100	100																												
29	A/blue-winged_teal/Guatemala/CIP049H123_59/2014	OP144108	96.8	97.3	97	97	96.8	96.8	96.7	96.9	96.8	96.8	96.8	96.8	98.4	96.9	98.4	99	99	99.1	99	99	99	99	98.9	99.9	99.6	99.9	99.9	99.9																											
30	A/blue-winged_teal/Guatemala/CIP049H123_61/2014	OP144116	96.8	97.4	97	97.1	96.9	96.8	96.8	97	96.9	96.9	96.9	96.9	98.4	97	98.4	99.1	99.1	99.1	99.1	99.1	99.1	99.1	99	100	99.7	100	100	100	99.9																										
31	A/blue-winged_teal/Guatemala/CIP049H123_65/2014	OP144124	96.8	97.3	97	97	96.8	96.8	96.7	96.9	96.8	96.8	96.8	96.8	98.4	96.9	98.4	99	99	99.1	99	99	99	99	98.9	99.8	99.5	99.8	99.8	99.8	99.8	99.8																									
32	A/blue-winged_teal/Guatemala/CIP049H123_75/2014	OP144133	96.6	97.1	96.8	96.8	96.7	96.6	96.5	96.7	96.7	96.7	96.7	96.7	98.2	96.7	98.2	98.8	98.8	98.9	98.8	98.8	98.8	98.8	98.8	99.6	99.9	99.6	99.6	99.6	99.6	99.6	99.5																								
33	A/blue-winged_teal/Guatemala/CIP049H123_76/2014	OP144141	96.8	97.4	97	97.1	96.9	96.8	96.8	97	96.9	96.9	96.9	96.9	98.4	97	98.4	99.1	99.1	99.1	99.1	99.1	99.1	99.1	99	100	99.7	100	100	100	99.9	100	99.8	99.6																							
34	A/blue-winged_teal/Guatemala/CIP049H125_14/2015	OP144150	96.5	97.1	96.7	96.8	96.6	96.5	96.5	96.7	96.6	96.6	96.6	96.6	98.1	96.7	98.1	98.8	98.8	98.8	98.8	98.8	98.8	98.8	98.7	99.6	99.3	99.6	99.6	99.6	99.5	99.6	99.8	99.2	99.6																						
35	A/blue-winged_teal/Guatemala/CIP049H125_23/2015	OP144158	96.5	97.1	96.7	96.8	96.6	96.5	96.5	96.7	96.6	96.6	96.6	96.6	98.1	96.7	98.1	98.8	98.8	98.8	98.8	98.8	98.8	98.8	98.7	99.6	99.3	99.6	99.6	99.6	99.5	99.6	99.8	99.2	99.6	99.9																					
36	A/blue-winged_teal/Guatemala/CIP049H125_59/2015	OP144166	96.6	97.1	96.8	96.8	96.7	96.6	96.5	96.7	96.7	96.7	96.7	96.7	98.2	96.7	98.2	98.8	98.8	98.9	98.8	98.8	98.8	98.8	98.8	99.6	99.4	99.6	99.6	99.6	99.6	99.6	99.8	99.3	99.6	99.9	99.9																				
37	A/blue-winged_teal/Guatemala/CIP049H125_108/2015	OP144174	96.5	97	96.7	96.7	96.5	96.5	96.4	96.6	96.5	96.5	96.5	96.5	98.1	96.6	98.1	98.7	98.7	98.8	98.7	98.7	98.7	98.7	98.7	99.5	99.2	99.5	99.5	99.5	99.5	99.5	99.7	99.2	99.5	99.8	99.8	99.9																			
38	A/blue-winged_teal/Guatemala/CIP049H158_26/2019	OP144182	95.1	95.4	95.1	94.8	94.8	94.7	94.7	94.7	94.8	94.7	94.7	94.7	94.8	94.7	94.8	94.3	94.3	94.2	94.3	94.3	94.3	94.3	94.3	93.9	93.7	93.9	93.9	93.9	93.8	93.9	93.8	93.7	93.9	93.7	93.7	93.8	93.8																		
39	A/blue-winged_teal/Guatemala/CIP049H189_08/2019	OP144190	94.8	95.2	94.9	94.6	94.6	94.5	94.5	94.6	94.6	94.5	94.4	94.3	94.4	94.5	94.4	94	94	94	94	94	94	94	94	93.8	93.6	93.8	93.8	93.8	93.7	93.8	93.7	93.5	93.8	93.6	93.6	93.7	93.7	98.1																	
40	A/blue-winged_teal/Guatemala/CIP049H189_19/2019	OP144199	94.8	95.2	94.9	94.6	94.6	94.5	94.5	94.6	94.6	94.5	94.4	94.3	94.4	94.5	94.4	94	94	94	94	94	94	94	94	93.8	93.6	93.8	93.8	93.8	93.7	93.8	93.7	93.5	93.8	93.6	93.6	93.7	93.7	98.1	100																
41	A/Blue_winged_Teal/Ohio/18OS1695/2018	MN431050	94.7	95.1	94.8	94.5	94.5	94.4	94.4	94.4	94.5	94.4	94.4	94.3	94.4	94.4	94.4	93.9	93.9	93.8	93.9	93.9	93.9	93.9	93.8	93.4	93.3	93.4	93.4	93.4	93.4	93.4	93.4	93.2	93.4	93.3	93.3	93.3	93.3	97.5	97.7	97.7															
42	A/blue_winged_teal/Texas/UGAI15_6890/2015	KX024583	96.4	96.8	96.5	96	96	95.9	95.8	95.9	96	95.8	95.8	95.7	96.1	95.9	96.1	95.5	95.5	95.5	95.5	95.5	95.5	95.5	95.5	95.2	95	95.2	95.2	95.2	95.1	95.2	95.1	95	95.2	94.9	94.9	95	95	97.4	97.2	97.2	97.1														
43	A/long_tailed_duck/Wisconsin/10OS3912/2010	JN696314	96.6	97.1	96.8	96.2	96.1	96	96	96.1	96.1	96	96	95.9	96.1	96.1	96.1	95.8	95.8	95.7	95.8	95.8	95.8	95.8	95.7	95.1	95	95.1	95.1	95.1	95.1	95.1	95	95	95.1	94.8	94.8	94.8	94.7	93.8	93.7	93.7	93.6	94.6													
44	A/long_tailed_duck/Wisconsin/10OS3918/2010	CY133553	96.6	97.1	96.8	96.2	96.1	96	96	96.1	96.1	96	96	95.9	96.1	96.1	96.1	95.8	95.8	95.7	95.8	95.8	95.8	95.8	95.7	95.1	95	95.1	95.1	95.1	95.1	95.1	95	95	95.1	94.8	94.8	94.8	94.7	93.8	93.7	93.7	93.6	94.6	100												
45	A/long_tailed_duck/Wisconsin/10OS4225/2010	JN696316	96.6	97.1	96.8	96.2	96.1	96	96	96.1	96.1	96	96	95.9	96.1	96.1	96.1	95.8	95.8	95.7	95.8	95.8	95.8	95.8	95.7	95.1	95	95.1	95.1	95.1	95.1	95.1	95	95	95.1	94.8	94.8	94.8	94.7	93.8	93.7	93.7	93.6	94.6	100	100											
46	A/mallard/Wisconsin/10OS3941/2010	CY133266	96.5	97.1	96.7	96.1	96	96	95.9	96	96	96	96	95.8	96.1	96	96.1	95.7	95.7	95.7	95.7	95.7	95.7	95.7	95.7	95.1	95.1	95.1	95.1	95.1	95.2	95.1	95.1	95	95.1	94.8	94.8	94.9	94.8	93.8	93.7	93.7	93.5	94.7	99.9	99.9	99.9										
47	A/northern_shoveler/California/2696/2011	CY146897	98.8	99.2	98.9	98.3	98.1	98.1	98	98.2	98.1	98.1	98.1	98	98.2	98.2	98.2	97.7	97.7	97.7	97.7	97.7	97.7	97.7	97.7	96.9	96.7	96.9	96.9	96.9	96.8	96.9	96.8	96.7	96.9	96.6	96.6	96.7	96.5	95.5	95.2	95.2	95.2	96.9	96.8	96.8	96.8	96.8									
48	A/Northern_Shoveler/California/D1616217/2016	MK928228	95.8	96.2	96	95.4	95.4	95.3	95.2	95.3	95.4	95.2	95.2	95.1	95.6	95.3	95.6	95.2	95.2	95.1	95.2	95.2	95.2	95.2	95.1	95	94.8	95	95	95	94.9	95	94.9	94.7	95	94.7	94.7	94.7	94.7	97.8	97.7	97.7	97.4	98.5	94.6	94.6	94.6	94.7	96.3								
49	A/northern_shoveler/Mississippi/12OS456/2012	CY167267	98.8	99.4	98.9	98.3	98.1	98.1	98	98.2	98.1	98.1	98.1	98	98.2	98.2	98.2	97.8	97.8	97.7	97.8	97.8	97.8	97.8	97.7	97	96.9	97	97	97	96.9	97	96.9	96.8	97	96.7	96.7	96.7	96.6	95.1	94.9	94.9	94.8	96.5	96.8	96.8	96.8	96.8	98.8	96							
50	A/northern_shoveler/Missouri/10OS4673/2010	CY133381	97.2	97.8	97.4	96.8	96.7	96.6	96.5	96.7	96.7	96.7	96.7	96.5	96.9	96.7	96.9	96.4	96.4	96.4	96.4	96.4	96.4	96.4	96.4	95.6	95.4	95.6	95.6	95.6	95.7	95.6	95.5	95.4	95.6	95.3	95.3	95.4	95.2	94.3	94.1	94.1	94	95.2	97.1	97.1	97.1	97	97.2	94.8	97.4						
51	A/Northern_shoveler/Missouri/16OS6248/2016	MG280044	95.5	96	95.7	95.4	95.3	95.2	95.2	95.2	95.3	95.2	95.2	95.1	95.3	95.2	95.3	94.8	94.8	94.7	94.8	94.8	94.8	94.8	94.7	94.4	94.3	94.4	94.4	94.4	94.4	94.4	94.4	94.2	94.4	94.1	94.1	94.2	94.2	97.6	97.2	97.2	97.1	98.1	94	94	94	94	96	98.4	95.7	94.8					
52	A/white_winged_scoter/Wisconsin/10OS3922/2010	JN696315	96.5	97.1	96.7	96.1	96	96	95.9	96	96	96	96	95.8	96.1	96	96.1	95.7	95.7	95.7	95.7	95.7	95.7	95.7	95.7	95	95	95	95	95	95.1	95	95	94.9	95	94.7	94.7	94.8	94.7	93.7	93.6	93.6	93.5	94.5	99.9	99.9	99.9	99.9	96.8	94.5	96.8	97	93.9				
53	A/Common_Teal/Chany_Lake/29/2019	EPI_ISL_400267	89.6	90.2	89.9	89.8	89.8	89.8	89.7	89.7	89.8	89.6	89.6	89.6	89.5	89.7	89.5	89.2	89.2	89.1	89.2	89.2	89.2	89.2	89.1	88.9	88.8	88.9	88.9	88.9	88.9	88.9	88.9	88.7	88.9	88.7	88.7	88.7	88.7	88.3	88.4	88.4	88	88.7	90.2	90.2	90.2	90.1	89.8	88.7	89.9	90.4	88.7	90.1			
54	A/garganey/Ukraine/05839-NAMRU3/2006	EPI_ISL_120215	95	95.5	95.1	94.8	94.7	94.6	94.5	94.7	94.7	94.6	94.6	94.6	95	94.7	95	94.5	94.5	94.4	94.5	94.5	94.5	94.5	94.4	93.8	93.6	93.8	93.8	93.8	93.8	93.8	93.7	93.5	93.8	93.5	93.5	93.5	93.4	92.5	92.5	92.5	92.2	93.3	96	96	96	95.9	95.2	93.3	95.1	95.5	93	95.9	92.1		
55	A/goose/Karachi/NARC_13N_969/2014	KX602668	91.6	92.1	91.9	91.6	91.6	91.5	91.4	91.4	91.6	91.4	91.4	91.4	91.6	91.4	91.6	91.1	91.1	91.1	91.1	91.1	91.1	91.1	91.1	90.8	90.6	90.8	90.8	90.8	90.8	90.8	90.7	90.6	90.8	90.5	90.5	90.6	90.5	89.9	89.6	89.6	89.8	90.7	92.3	92.3	92.3	92.3	91.8	90.7	91.9	92.3	90.4	92.3	95.4	94.4	
56	A/mallard/Astrakhan/263/1982	AB289335	88.5	88.9	88.7	88.2	88.2	88.2	88	88.1	88.2	88	88	88.2	88.3	88.2	88.3	88	88	87.9	88	88	88	88	87.9	87.8	87.6	87.8	87.8	87.8	87.8	87.8	87.7	87.6	87.8	87.6	87.6	87.7	87.6	87.7	87.4	87.4	87.7	87.7	89.1	89.1	89.1	89.1	88.6	87.9	88.5	89.1	88.3	89	88	90	89	
