# Supplementary material for: Blue-Winged Teals in Guatemala and Their Potential Role in the Ecology of H14 Subtype Influenza a Viruses
Source: Viruses. 2023 Feb 9;15(2):483. doi: 10.3390/v15020483 (PMC9961055; doi:10.3390/v15020483)
Supplement: Supplementary file 1 [file Suppl_Table S6.pdf › Suppl_Table S6.pdf]

Suppl. Table S6. Detailed nucleotide pairwise identity of ORF sequences of NP gene segment of full-length H14 viruses from Guatemala (n=40), North America (n=12), and Eurasia (n=4) during 1982-2019.

# virus	Strain	Reference ID	1	2	3	4	5	6	7	8	9	10	11	12	13	14	15	16	17	18	19	20	21	22	23	24	25	26	27	28	29	30	31	32	33	34	35	36	37	38	39	40	41	42	43	44	45	46	47	48	49	50	51	52	53	54	55	56
1	A/blue_winged_teal/Guatemala/CIP049H105_15/2011	KJ195677																																																						
2	A/blue_winged_teal/Guatemala/CIP049H105_31/2011	KY644352	93.4																																																						
3	A/blue_winged_teal/Guatemala/CIP049H106_62/2011	KJ195669	99	93.4																																																				
4	A/blue_winged_teal/Guatemala/CIP049H108_02/2012	KY644443	92.2	95	92.1																																																		
5	A/blue_winged_teal/Guatemala/CIP049H108_04/2012	KX960410	92.2	94.9	92.1	99.8																																																
6	A/blue_winged_teal/Guatemala/CIP049H108_11/2012	KX960390	92.2	95	92.1	99.9	99.9																																														
7	A/blue_winged_teal/Guatemala/CIP049H109_49/2012	KY644359	92.2	95	92.1	99.9	99.9	100																																												
8	A/blue_winged_teal/Guatemala/CIP049H109_67/2012	KY673152	91.8	94.3	91.8	97.9	97.9	97.9	97.9																																										
9	A/blue_winged_teal/Guatemala/CIP049H109_76/2012	KX960397	92.2	95	92.1	99.9	99.9	100	100	97.9																																								
10	A/blue_winged_teal/Guatemala/CIP049H110_04/2012	KY644311	91.8	94	91.8	98	98.1	98.1	98.1	97.8	98.1																																						
11	A/blue_winged_teal/Guatemala/CIP049H110_31/2012	KY644483	91.8	94.1	91.8	94.1	94	94.1	94.1	93.5	94.1	93.6																																				
12	A/blue_winged_teal/Guatemala/CIP049H113_07/2013	KY644470	92.2	94.2	92.1	98.1	98.1	98.1	98.1	99.1	98.1	97.9	93.9																																		
13	A/blue_winged_teal/Guatemala/CIP049H113_08/2013	KY644259	92.2	95	92.2	99.8	99.7	99.8	99.8	97.9	99.8	97.9	94.1	98.1																																	
14	A/blue_winged_teal/Guatemala/CIP049H113_74/2013	KY673168	91.9	94.9	91.8	99.6	99.5	99.6	99.6	97.7	99.6	97.7	94.1	97.9	99.5																															
15	A/blue_winged_teal/Guatemala/CIP049H113_76/2013	KY644344	92.2	95	92.2	99.8	99.7	99.8	99.8	97.9	99.8	97.9	94.1	98.1	100	99.5																														
16	A/blue_winged_teal/Guatemala/CIP049H116_120/2013	MK327719	91.8	93.7	91.8	97.3	97.2	97.3	97.3	98.2	97.3	97	93.2	98.6	97.2	97.1	97.2																													
17	A/blue_winged_teal/Guatemala/CIP049H117_13/2013	MK327687	91.9	93.8	91.8	97.2	97.1	97.2	97.2	98.1	97.2	96.9	93.2	98.5	97.1	96.9	97.1	99.8																												
18	A/blue_winged_teal/Guatemala/CIP049H117_34/2013	MK327695	90.4	89.8	90.4	89.6	89.6	89.6	89.6	89.5	89.6	90.1	89.7	89.8	89.7	89.6	89.7	89.3	89.2																											
19	A/blue_winged_teal/Guatemala/CIP049H117_36/2013	MK327703	92	94.4	91.8	94.3	94.3	94.3	94.3	93.6	94.3	93.9	98.1	94	94.3	94.3	94.3	93.3	93.4	90.2																										
20	A/blue_winged_teal/Guatemala/CIP049H117_38/2013	MK326657	92	94.4	91.8	94.3	94.3	94.3	94.3	93.6	94.3	93.9	98.1	94	94.3	94.3	94.3	93.3	93.4	90.2	100																									
21	A/blue_winged_teal/Guatemala/CIP049H117_99/2013	MK327735	91.8	94.3	91.8	97.7	97.6	97.7	97.7	97.2	97.7	97.5	93.7	97.5	97.7	97.4	97.7	96.5	96.5	89.8	93.4	93.4																								
22	A/blue_winged_teal/Guatemala/CIP049H117_123/2013	MK327727	91.8	93.7	91.8	97.3	97.2	97.3	97.3	98.2	97.3	97	93.2	98.6	97.2	97.1	97.2	100	99.8	89.3	93.3	93.3	96.5																							
23	A/blue_winged_teal/Guatemala/CIP049H117_143/2013	MK327759	90.5	89.8	90.5	89.6	89.6	89.6	89.6	89.5	89.6	90.1	89.8	89.8	89.7	89.6	89.7	89.4	89.3	99.9	90.3	90.3	89.8	89.4																						
24	A/blue-winged_teal/Guatemala/CIP049H123_13/2014	OP144069	91.8	94.6	92	97.5	97.5	97.5	97.5	96.8	97.5	97.3	93.3	97.1	97.6	97.3	97.6	96.3	96.2	89.6	93.4	93.4	98	96.3	89.6																					
25	A/blue-winged_teal/Guatemala/CIP049H123_16/2014	OP144077	92	94.3	91.8	98.1	98.1	98.1	98.1	97.4	98.1	97.7	93.9	97.6	98.1	98	98.1	96.9	96.8	89.8	94	94	97.3	96.9	89.8	97.1																				
26	A/blue-winged_teal/Guatemala/CIP049H123_33/2014	OP144085	91.7	94.7	91.9	97.6	97.5	97.6	97.6	96.9	97.6	97.3	93.4	97.2	97.7	97.3	97.7	96.3	96.3	89.6	93.5	93.5	98.1	96.3	89.6	99.9	97.2																			
27	A/blue-winged_teal/Guatemala/CIP049H123_57/2014	OP144093	91.8	94.6	92	97.5	97.5	97.5	97.5	96.8	97.5	97.3	93.3	97.1	97.6	97.3	97.6	96.3	96.2	89.6	93.4	93.4	98	96.3	89.6	100	97.1	99.9																		
28	A/blue-winged_teal/Guatemala/CIP049H123_58/2014	OP144101	91.8	94.6	92	97.5	97.5	97.5	97.5	96.8	97.5	97.3	93.3	97.1	97.6	97.3	97.6	96.3	96.2	89.6	93.4	93.4	98	96.3	89.6	100	97.1	99.9	100																	
29	A/blue-winged_teal/Guatemala/CIP049H123_59/2014	OP144109	91.8	94.6	92	97.5	97.5	97.5	97.5	96.8	97.5	97.3	93.3	97.1	97.6	97.3	97.6	96.3	96.2	89.6	93.4	93.4	98	96.3	89.6	100	97.1	99.9	100	100																
30	A/blue-winged_teal/Guatemala/CIP049H123_61/2014	OP144117	91.8	94.5	92	97.5	97.4	97.5	97.5	96.7	97.5	97.2	93.2	97.1	97.5	97.2	97.5	96.2	96.1	89.6	93.4	93.4	97.9	96.2	89.6	99.9	97.1	99.9	99.9	99.9	99.9															
31	A/blue-winged_teal/Guatemala/CIP049H123_65/2014	OP144125	91.8	94.6	92	97.5	97.5	97.5	97.5	96.8	97.5	97.3	93.3	97.1	97.6	97.3	97.6	96.3	96.2	89.6	93.4	93.4	98	96.3	89.6	100	97.1	99.9	100	100	100	99.9														
32	A/blue-winged_teal/Guatemala/CIP049H123_75/2014	OP144134	92	94.3	91.8	98.1	98.1	98.1	98.1	97.4	98.1	97.7	93.9	97.6	98.1	98	98.1	96.9	96.8	89.8	94	94	97.3	96.9	89.8	97.1	100	97.2	97.1	97.1	97.1	97.1	97.1													
33	A/blue-winged_teal/Guatemala/CIP049H123_76/2014	OP144142	91.8	94.6	92	97.5	97.5	97.5	97.5	96.8	97.5	97.3	93.3	97.1	97.6	97.3	97.6	96.3	96.2	89.6	93.4	93.4	98	96.3	89.6	100	97.1	99.9	100	100	100	99.9	100	97.1												
34	A/blue-winged_teal/Guatemala/CIP049H125_14/2015	OP144151	91.5	94.1	91.8	97.7	97.6	97.7	97.7	96.9	97.7	97.1	93.4	97.3	97.9	97.4	97.9	96.3	96.2	89.8	93.4	93.4	98.1	96.3	89.8	98.1	97	98.2	98.1	98.1	98.1	98.1	98.1	97	98.1											
35	A/blue-winged_teal/Guatemala/CIP049H125_23/2015	OP144159	96.7	93.6	96.3	92.1	92.1	92.1	92.1	91.8	92.1	91.8	91.5	92	92.2	91.8	92.2	91.4	91.4	90.4	91.8	91.8	91.7	91.4	90.4	92	91.7	91.9	92	92	92	92	92	91.7	92	91.7										
36	A/blue-winged_teal/Guatemala/CIP049H125_59/2015	OP144167	91.7	94.3	92	97.7	97.7	97.7	97.7	96.9	97.7	97.2	93.5	97.2	97.9	97.5	97.9	96.2	96.1	90	93.5	93.5	98.2	96.2	90	98.2	97.1	98.3	98.2	98.2	98.2	98.1	98.2	97.1	98.2	99.7	91.9									
37	A/blue-winged_teal/Guatemala/CIP049H125_108/2015	OP144175	96.7	93.6	96.3	92.1	92.1	92.1	92.1	91.8	92.1	91.8	91.5	92	92.2	91.8	92.2	91.4	91.4	90.4	91.8	91.8	91.7	91.4	90.4	92	91.7	91.9	92	92	92	92	92	91.7	92	91.7	100	91.9								
38	A/blue-winged_teal/Guatemala/CIP049H158_26/2019	OP144183	90.8	93	91	96.3	96.3	96.3	96.3	96.1	96.3	96.1	92.6	96.2	96.5	96.2	96.5	95.3	95.3	89.4	92.6	92.6	96.7	95.3	89.4	96.8	96.1	96.9	96.8	96.8	96.8	96.7	96.8	96.1	96.8	98.1	90.8	98.2	90.8							
39	A/blue-winged_teal/Guatemala/CIP049H189_08/2019	OP144192	91.6	93.9	91.8	97.3	97.2	97.3	97.3	97.4	97.3	97	93.2	98	97.2	97	97.2	96.8	96.8	89.4	93.4	93.4	96.9	96.8	89.4	96.6	96.7	96.7	96.6	96.6	96.6	96.5	96.6	96.7	96.6	96.6	91.8	96.5	91.8	95.3																		
40	A/blue-winged_teal/Guatemala/CIP049H189_19/2019	OP144200	92.8	97.9	92.6	93.9	93.9	93.9	93.9	93.2	93.9	92.8	93.2	93.1	93.9	93.8	93.9	92.6	92.7	90.2	93.5	93.5	93.2	92.6	90.2	93.4	93.3	93.5	93.4	93.4	93.4	93.4	93.4	93.3	93.4	93	92.8	93.2	92.8	92	92.7																
41	A/Blue_winged_Teal/Ohio/18OS1695/2018	MN431048	90.6	92.9	90.8	96.2	96.2	96.2	96.2	96	96.2	96.1	92.4	96.2	96.4	96.1	96.4	95.6	95.5	89.3	92.6	92.6	96.5	95.6	89.3	96.7	95.8	96.7	96.7	96.7	96.7	96.6	96.7	95.8	96.7	98	90.8	98.1	90.8	99	95.5	91.8															
42	A/blue_winged_teal/Texas/UGAI15_6890/2015	KX024584	98.9	93.4	98.4	92	92	92	92	91.7	92	91.6	91.3	92	92.1	91.8	92.1	91.6	91.7	90.1	91.6	91.6	91.6	91.6	90.2	91.8	91.8	91.7	91.8	91.8	91.8	91.8	91.8	91.8	91.8	91.4	96.2	91.6	96.2	90.6	91.5	92.6	90.4														
43	A/long_tailed_duck/Wisconsin/10OS3912/2010	KC110597	98.9	93.7	98.5	92.2	92.2	92.2	92.2	91.8	92.2	91.7	91.8	92.2	92.3	92	92.3	91.9	92	90.4	91.9	91.9	92	91.9	90.5	92	92	91.9	92	92	92	92	92	92	92	91.7	96.9	91.9	96.9	91	91.8	93	90.8	98.3													
44	A/long_tailed_duck/Wisconsin/10OS3918/2010	CY133556	93.2	94.5	92.7	94.1	94	94.1	94.1	93.4	94.1	93.2	93.2	93.6	94.1	93.9	94.1	92.8	92.8	90.1	93.3	93.3	93.2	92.8	90.1	93.1	93.9	93.2	93.1	93.1	93.1	93	93.1	93.9	93.1	92.8	93.4	93	93.4	91.9	93.2	93.7	91.5	93.2	93.2												
45	A/long_tailed_duck/Wisconsin/10OS4225/2010	KC110602	93.2	94.5	92.7	94	93.9	94	94	93.3	94	93.1	93.2	93.5	94	93.8	94	92.7	92.7	90.1	93.3	93.3	93.1	92.7	90.1	93	93.8	93.1	93	93	93	92.9	93	93.8	93	92.7	93.5	92.9	93.5	91.8	93.1	93.8	91.4	93.1	93.2	99.9											
46	A/mallard/Wisconsin/10OS3941/2010	CY133269	93.2	94.5	92.7	94.1	94	94.1	94.1	93.4	94.1	93.2	93.2	93.6	94.1	93.9	94.1	92.8	92.8	90.1	93.3	93.3	93.2	92.8	90.1	93.1	93.9	93.2	93.1	93.1	93.1	93	93.1	93.9	93.1	92.8	93.4	93	93.4	91.9	93.2	93.7	91.5	93.2	93.2	100	99.9										
47	A/northern_shoveler/California/2696/2011	CY146900	93.2	93.9	92.8	93.7	93.6	93.7	93.7	93.2	93.7	93.1	93.1	93.5	93.8	93.6	93.8	92.6	92.7	89.7	93	93	93.3	92.6	89.7	93	93.5	93.1	93	93	93	93	93	93.5	93	93	93	93.2	93	92	93.4	93.4	91.8	93	93.2	96.7	96.6	96.7									
48	A/Northern_Shoveler/California/D1616217/2016	MK928231	91.2	93.5	91.4	96.6	96.5	96.6	96.6	96.1	96.6	96.3	92.8	96.3	96.8	96.3	96.8	95.3	95.3	89.8	93	93	97.1	95.3	89.8	97.3	96.3	97.4	97.3	97.3	97.3	97.3	97.3	96.3	97.3	98.5	91.4	98.6	91.4	97.6	95.9	92.7	97.3	91.1	91.4	92.6	92.5	92.6	92.6								
49	A/northern_shoveler/Mississippi/12OS456/2012	CY167270	92.2	94.7	92.2	98.3	98.2	98.3	98.3	97.7	98.3	98	93.7	97.9	98.2	98.1	98.2	97	96.9	89.5	93.9	93.9	97.7	97	89.5	97.5	98.1	97.6	97.5	97.5	97.5	97.5	97.5	98.1	97.5	97.4	91.8	97.5	91.8	96.1	97	93.6	95.9	92	92.1	93.6	93.5	93.6	93.4	96.6							
50	A/northern_shoveler/Missouri/10OS4673/2010	CY133384	91.9	94.7	91.9	98.6	98.5	98.6	98.6	98.2	98.6	98.3	94.1	98.3	98.5	98.3	98.5	97.5	97.4	89.8	94.3	94.3	97.9	97.5	89.8	97.7	98.2	97.8	97.7	97.7	97.7	97.7	97.7	98.2	97.7	97.6	91.8	97.7	91.8	96.5	97.3	93.5	96.3	91.8	92.1	93.9	93.8	93.9	93.6	96.9	98.5						
51	A/Northern_shoveler/Missouri/16OS6248/2016	MG279946	91.7	93.8	91.9	97.1	97	97.1	97.1	96.7	97.1	96.9	93.2	96.9	97.3	96.8	97.3	95.9	95.9	89.7	93.2	93.2	97.9	95.9	89.7	97.7	96.7	97.7	97.7	97.7	97.7	97.6	97.7	96.7	97.7	99.1	91.9	99.2	91.9	97.9	96.1	92.8	97.8	91.6	91.9	92.8	92.7	92.8	93.1	98.5	96.9	97.1					
52	A/white_winged_scoter/Wisconsin/10OS3922/2010	KC110611	93.2	94.5	92.8	94.1	94.1	94.1	94.1	93.4	94.1	93.3	93.2	93.7	94.2	94	94.2	92.8	92.9	90.2	93.4	93.4	93.3	92.8	90.2	93.2	94	93.2	93.2	93.2	93.2	93.1	93.2	94	93.2	92.9	93.5	93.1	93.5	92	93.2	93.8	91.6	93.2	93.2	99.9	99.8	99.9	96.7	92.7	93.7	93.9	92.8				
53	A/Common_Teal/Chany_Lake/29/2019	EPI ISL 400267	90.9	89.8	90.4	88.8	88.9	88.9	88.9	88.5	88.9	88.9	89.2	88.9	88.9	88.8	88.9	88.2	88.3	93	89.5	89.5	89.2	88.2	93	88.6	88.9	88.7	88.6	88.6	88.6	88.7	88.6	88.9	88.6	88.5	90.5	88.7	90.5	88.1	88.4	89.5	88.1	90.5	90.7	89.7	89.7	89.7	89.5	88.6	88.7	88.6	88.8	89.7			
54	A/garganey/Ukraine/05839-NAMRU3/2006	EPI ISL 120215	89.8	89.2	89.6	88.8	88.8	88.9	88.9	88.2	88.9	89	88.8	88.6	88.8	88.7	88.8	88.2	88.1	92.7	89	89	88.8	88.2	92.8	88.4	89.2	88.4	88.4	88.4	88.4	88.4	88.4	89.2	88.4	88.5	90	88.7	90	88.2	88.3	88.6	88	89.6	89.9	89.4	89.5	89.4	88.8	88.6	88.4	88.6	88.4	89.5	93.9		
55	A/goose/Karachi/NARC_13N_969/2014	KX602669	89.8	89.5	89.6	89	88.9	89	89	88.6	89	89	89.3	89	89	89	89	88.6	88.5	92.8	89.6	89.6	89.1	88.6	92.9	88.9	89.4	89	88.9	88.9	88.9	89	88.9	89.4	88.9	88.8	90	89	90	88.4	88.8	89.5	88.4	89.2	89.6	89.2	89.3	89.2	88.8	88.9	88.8	89	88.8	89.3	94.8	94.5	
56	A/mallard/Astrakhan/263/1982	CY005392	90.8	89.8	90.5	90.1	90.2	90.2	90.2	89.6	90.2	90.7	90	90.2	90.2	90.1	90.2	89.4	89.5	94.9	90.2	90.2	90.4	89.4	95	90.4	90.4	90.3	90.4	90.4	90.4	90.4	90.4	90.4	90.4	90.2	90.7	90.4	90.7	89.8	90.1	89.6	89.8	90.6	90.6	90.3	90.3	90.3	89.6	90.2	89.9	89.9	90.2	90.4	94.7	95.8	95.7	
