# Supplementary material for: Blue-Winged Teals in Guatemala and Their Potential Role in the Ecology of H14 Subtype Influenza a Viruses
Source: Viruses. 2023 Feb 9;15(2):483. doi: 10.3390/v15020483 (PMC9961055; doi:10.3390/v15020483)
Supplement: Supplementary file 1 [file Suppl_Table S7.pdf › Suppl_Table S7.pdf]

Suppl. Table S7. Detailed nucleotide pairwise identity of ORF sequences of M gene segment of full-length H14 viruses from Guatemala (n=40), North America (n=12), and Eurasia (n=4) during 1982-2019.

# virus	Strain	Reference ID	1	2	3	4	5	6	7	8	9	10	11	12	13	14	15	16	17	18	19	20	21	22	23	24	25	26	27	28	29	30	31	32	33	34	35	36	37	38	39	40	41	42	43	44	45	46	47	48	49	50	51	52	53	54	55	56
1	A/blue_winged_teal/Guatemala/CIP049H105_15/2011	KJ195679																																																						
2	A/blue_winged_teal/Guatemala/CIP049H105_31/2011	KY644338	98.4																																																					
3	A/blue_winged_teal/Guatemala/CIP049H106_62/2011	KJ195671	98.5	98.3																																																			
4	A/blue_winged_teal/Guatemala/CIP049H108_02/2012	KY644312	98.8	98.3	97.9																																																	
5	A/blue_winged_teal/Guatemala/CIP049H108_04/2012	KX960465	99.2	98.7	98.3	99.6																																															
6	A/blue_winged_teal/Guatemala/CIP049H108_11/2012	KX960489	99.2	98.7	98.3	99.6	100																																													
7	A/blue_winged_teal/Guatemala/CIP049H109_49/2012	KY644246	99.2	98.7	98.3	99.6	100	100																																												
8	A/blue_winged_teal/Guatemala/CIP049H109_67/2012	KY673161	97.8	97.8	98.2	97.1	97.5	97.5	97.5																																											
9	A/blue_winged_teal/Guatemala/CIP049H109_76/2012	KX960478	99.2	98.7	98.3	99.6	100	100	100	97.5																																										
10	A/blue_winged_teal/Guatemala/CIP049H110_04/2012	KY644230	98.7	98.7	98.8	98	98.4	98.4	98.4	98	98.4																																									
11	A/blue_winged_teal/Guatemala/CIP049H110_31/2012	KY644298	98.2	97.9	99.3	97.5	97.9	97.9	97.9	98	97.9	98.4																																								
12	A/blue_winged_teal/Guatemala/CIP049H113_07/2013	KY644495	98.8	98.3	97.9	99.7	99.3	99.3	99.3	97.1	99.3	98	97.5																																							
13	A/blue_winged_teal/Guatemala/CIP049H113_08/2013	KY644238	98.3	99.6	98.2	97.9	98.3	98.3	98.3	97.9	98.3	98.5	97.8	98.2																																						
14	A/blue_winged_teal/Guatemala/CIP049H113_74/2013	KY673167	98.3	99.6	98.2	97.9	98.3	98.3	98.3	97.9	98.3	98.5	97.8	98.2	100																																					
15	A/blue_winged_teal/Guatemala/CIP049H113_76/2013	KY644277	98.3	99.6	98.2	97.9	98.3	98.3	98.3	97.9	98.3	98.5	97.8	98.2	100	100																																				
16	A/blue_winged_teal/Guatemala/CIP049H116_120/2013	MK327721	98.2	98.2	98.5	97.5	97.9	97.9	97.9	97.8	97.9	98.4	98.2	97.5	98	98	98																																			
17	A/blue_winged_teal/Guatemala/CIP049H117_13/2013	MK327689	99.2	98.2	98.3	98.5	98.9	98.9	98.9	97.5	98.9	98.4	97.9	98.5	98	98	98	97.9																																		
18	A/blue_winged_teal/Guatemala/CIP049H117_34/2013	MK327697	97	97	97.1	96.8	97.2	97.2	97.2	96.6	97.2	97.2	97	96.8	96.8	96.8	96.8	96.7	96.7																																	
19	A/blue_winged_teal/Guatemala/CIP049H117_36/2013	MK327705	98.2	97.9	98.2	97.2	97.6	97.6	97.6	97.4	97.6	98	97.8	97.2	97.8	97.8	97.8	98.8	97.6	96.8																																
20	A/blue_winged_teal/Guatemala/CIP049H117_38/2013	MK326659	98.2	97.9	98.2	97.2	97.6	97.6	97.6	97.4	97.6	98	97.8	97.2	97.8	97.8	97.8	98.8	97.6	96.8	100																															
21	A/blue_winged_teal/Guatemala/CIP049H117_99/2013	MK327737	98.2	97.9	98.2	97.2	97.6	97.6	97.6	97.4	97.6	98	97.8	97.2	97.8	97.8	97.8	98.8	97.6	96.8	100	100																														
22	A/blue_winged_teal/Guatemala/CIP049H117_123/2013	MK327729	98.2	98.2	98.5	97.5	97.9	97.9	97.9	97.8	97.9	98.4	98.2	97.5	98	98	98	100	97.9	96.7	98.8	98.8	98.8																													
23	A/blue_winged_teal/Guatemala/CIP049H117_143/2013	MK327761	96.8	96.8	97	96.7	97.1	97.1	97.1	96.4	97.1	97.1	96.8	96.7	96.7	96.7	96.7	96.6	96.6	99.9	96.7	96.7	96.7	96.6																												
24	A/blue-winged_teal/Guatemala/CIP049H123_13/2014	OP144071	98	98.5	98.4	97.4	97.8	97.8	97.8	98	97.8	98.5	98.2	97.6	98.4	98.4	98.4	98.8	97.8	96.7	98.4	98.4	98.4	98.8	96.6																											
25	A/blue-winged_teal/Guatemala/CIP049H123_16/2014	OP144079	96.4	96.7	96.2	96.3	96.7	96.7	96.7	96.4	96.7	96.3	96	96.3	96.8	96.8	96.8	96.3	96.4	97.2	96.3	96.3	96.3	96.3	97.1	96.6																										
26	A/blue-winged_teal/Guatemala/CIP049H123_33/2014	OP144087	97.9	99.2	97.8	97.5	97.9	97.9	97.9	97.5	97.9	98.2	97.4	97.8	99.3	99.3	99.3	97.9	97.6	96.4	97.6	97.6	97.6	97.9	96.3	98.3	96.7																									
27	A/blue-winged_teal/Guatemala/CIP049H123_57/2014	OP144095	98	98.5	98.4	97.4	97.8	97.8	97.8	98	97.8	98.5	98.2	97.6	98.4	98.4	98.4	98.8	97.8	96.7	98.4	98.4	98.4	98.8	96.6	100	96.6	98.3																								
28	A/blue-winged_teal/Guatemala/CIP049H123_58/2014	OP144103	98	98.5	98.4	97.4	97.8	97.8	97.8	98	97.8	98.5	98.2	97.6	98.4	98.4	98.4	98.8	97.8	96.7	98.4	98.4	98.4	98.8	96.6	100	96.6	98.3	100																							
29	A/blue-winged_teal/Guatemala/CIP049H123_59/2014	OP144111	98.2	98.4	98.5	97.5	97.9	97.9	97.9	98	97.9	98.7	98.2	97.5	98.3	98.3	98.3	99.2	97.9	96.7	98.5	98.5	98.5	99.2	96.6	99.6	96.6	98.2	99.6	99.6																						
30	A/blue-winged_teal/Guatemala/CIP049H123_61/2014	OP144119	98	98.5	98.4	97.4	97.8	97.8	97.8	98	97.8	98.5	98.2	97.6	98.4	98.4	98.4	98.8	97.8	96.7	98.4	98.4	98.4	98.8	96.6	100	96.6	98.3	100	100	99.6																					
31	A/blue-winged_teal/Guatemala/CIP049H123_65/2014	OP144128	98	98.5	98.4	97.4	97.8	97.8	97.8	98	97.8	98.5	98.2	97.6	98.4	98.4	98.4	98.8	97.8	96.7	98.4	98.4	98.4	98.8	96.6	100	96.6	98.3	100	100	99.6	100																				
32	A/blue-winged_teal/Guatemala/CIP049H123_75/2014	OP144136	96.4	96.7	96.2	96.3	96.7	96.7	96.7	96.4	96.7	96.3	96	96.3	96.8	96.8	96.8	96.3	96.4	97.2	96.3	96.3	96.3	96.3	97.1	96.6	100	96.7	96.6	96.6	96.6	96.6	96.6																			
33	A/blue-winged_teal/Guatemala/CIP049H123_76/2014	OP144145	98	98.5	98.4	97.4	97.8	97.8	97.8	98	97.8	98.5	98.2	97.6	98.4	98.4	98.4	98.8	97.8	96.7	98.4	98.4	98.4	98.8	96.6	100	96.6	98.3	100	100	99.6	100	100	96.6																		
34	A/blue-winged_teal/Guatemala/CIP049H125_14/2015	OP144153	98.3	99.1	98.3	97.9	98.3	98.3	98.3	98	98.3	98.7	97.9	98.2	99.2	99.2	99.2	98.2	98	97	98	98	98	98.2	96.8	98.5	96.7	98.8	98.5	98.5	98.4	98.5	98.5	96.7	98.5																	
35	A/blue-winged_teal/Guatemala/CIP049H125_23/2015	OP144161	98.3	98.3	98.7	97.9	98	98	98	97.9	98	98.5	98.3	97.9	98.2	98.2	98.2	99.1	98	96.8	98.7	98.7	98.7	99.1	96.7	98.9	96.4	98.3	98.9	98.9	99.1	98.9	98.9	96.4	98.9	98.3																
36	A/blue-winged_teal/Guatemala/CIP049H125_59/2015	OP144169	98.3	99.1	98.3	97.9	98.3	98.3	98.3	98	98.3	98.7	97.9	98.2	99.5	99.5	99.5	98.2	98	97	98	98	98	98.2	96.8	98.5	96.7	98.8	98.5	98.5	98.4	98.5	98.5	96.7	98.5	99.5	98.3															
37	A/blue-winged_teal/Guatemala/CIP049H125_108/2015	OP144177	99.2	99.2	99.1	98.5	98.9	98.9	98.9	98.5	98.9	99.2	98.7	98.5	99.1	99.1	99.1	98.9	98.9	97.5	98.7	98.7	98.7	98.9	97.4	98.8	96.7	98.7	98.8	98.8	98.9	98.8	98.8	96.7	98.8	99.1	99.1	99.1														
38	A/blue-winged_teal/Guatemala/CIP049H158_26/2019	OP144185	98.3	98.8	98.3	97.9	98.3	98.3	98.3	97.5	98.3	98.7	97.9	98.2	98.7	98.7	98.7	97.9	98	97	98	98	98	97.9	96.8	98.3	96.4	98.3	98.3	98.3	98.2	98.3	98.3	96.4	98.3	98.9	98	98.9	98.8													
39	A/blue-winged_teal/Guatemala/CIP049H189_08/2019	OP144194	97.9	98.7	97.9	97.5	97.9	97.9	97.9	97.4	97.9	98.3	97.5	97.8	98.5	98.5	98.5	98.3	97.6	96.6	98.4	98.4	98.4	98.3	96.4	98.7	96.7	98.4	98.7	98.7	98.5	98.7	98.7	96.7	98.7	99.1	98.4	98.8	98.7	98.5												
40	A/blue-winged_teal/Guatemala/CIP049H189_19/2019	OP144202	97.9	98.7	97.9	97.8	98.2	98.2	98.2	97.4	98.2	98.3	97.5	97.5	98.3	98.3	98.3	97.8	97.9	97.1	97.6	97.6	97.6	97.8	97	97.9	96.3	98.2	97.9	97.9	98	97.9	97.9	96.3	97.9	98.5	98.2	98.5	98.7	98.3	98.2											
41	A/Blue_winged_Teal/Ohio/18OS1695/2018	MN431045	97.9	98.4	97.8	97.5	97.9	97.9	97.9	97.8	97.9	97.9	97.6	97.8	98.5	98.5	98.5	97.6	97.6	96.7	97.4	97.4	97.4	97.6	96.6	97.8	96.4	98.4	97.8	97.8	97.6	97.8	97.8	96.4	97.8	98.5	97.8	98.5	98.7	98	97.9	97.6										
42	A/blue_winged_teal/Texas/UGAI15_6890/2015	KX024586	98.3	99.1	98.3	97.9	98.3	98.3	98.3	97.8	98.3	98.7	97.9	98.2	98.9	98.9	98.9	98.2	98	97	98	98	98	98.2	96.8	98.5	96.4	98.5	98.5	98.5	98.4	98.5	98.5	96.4	98.5	99.5	98.3	99.2	99.1	98.9	99.3	98.5	98.3									
43	A/long_tailed_duck/Wisconsin/10OS3912/2010	KC110599	97.2	96.7	97.1	96.8	97.2	97.2	97.2	96.6	97.2	97.2	96.7	96.8	96.8	96.8	96.8	96.7	97	99.2	96.8	96.8	96.8	96.7	99.1	96.6	97	96.4	96.6	96.6	96.7	96.6	96.6	97	96.6	97	96.8	97	97.5	96.7	96.3	96.8	96.7	96.7								
44	A/long_tailed_duck/Wisconsin/10OS3918/2010	CY133554	97.2	96.7	97.1	96.8	97.2	97.2	97.2	96.6	97.2	97.2	96.7	96.8	96.8	96.8	96.8	96.7	97	99.2	96.8	96.8	96.8	96.7	99.1	96.6	97	96.4	96.6	96.6	96.7	96.6	96.6	97	96.6	97	96.8	97	97.5	96.7	96.3	96.8	96.7	96.7	100							
45	A/long_tailed_duck/Wisconsin/10OS4225/2010	KC110606	97.2	96.7	97.1	96.8	97.2	97.2	97.2	96.6	97.2	97.2	96.7	96.8	96.8	96.8	96.8	96.7	97	99.2	96.8	96.8	96.8	96.7	99.1	96.6	97	96.4	96.6	96.6	96.7	96.6	96.6	97	96.6	97	96.8	97	97.5	96.7	96.3	96.8	96.7	96.7	100	100						
46	A/mallard/Wisconsin/10OS3941/2010	CY133267	97.2	96.7	97.1	96.8	97.2	97.2	97.2	96.6	97.2	97.2	96.7	96.8	96.8	96.8	96.8	96.7	97	99.2	96.8	96.8	96.8	96.7	99.1	96.6	97	96.4	96.6	96.6	96.7	96.6	96.6	97	96.6	97	96.8	97	97.5	96.7	96.3	96.8	96.7	96.7	100	100	100					
47	A/northern_shoveler/California/2696/2011	CY146898	98	97.8	97.9	97.1	97.5	97.5	97.5	97.6	97.5	98.3	97.8	97.1	97.9	97.9	97.9	97.8	97.5	96.8	97.6	97.6	97.6	97.8	96.7	97.6	95.9	97.5	97.6	97.6	97.8	97.6	97.6	95.9	97.6	98	98.2	98	98.5	97.5	97.4	97.4	97.8	97.8	97.1	97.1	97.1	97.1				
48	A/Northern_Shoveler/California/D1616217/2016	MK928229	99.1	98.5	98.7	98.4	98.8	98.8	98.8	97.9	98.8	98.8	98.3	98.4	98.4	98.4	98.4	98.3	98.8	97.1	98	98	98	98.3	97	98.2	96.3	98	98.2	98.2	98.3	98.2	98.2	96.3	98.2	98.4	98.4	98.4	99.3	98.4	98	98	98	98.4	97.1	97.1	97.1	97.1	97.9			
49	A/northern_shoveler/Mississippi/12OS456/2012	CY167268	98.3	98.3	98.4	97.6	98	98	98	99.2	98	98.7	98	97.6	98.4	98.4	98.4	98.3	98	96.8	97.9	97.9	97.9	98.3	96.7	98.2	96.4	98	98.2	98.2	98.3	98.2	98.2	96.4	98.2	98.5	98.4	98.5	99.1	98	97.9	97.9	98	98.3	97.1	97.1	97.1	97.1	98.2	98.4		
50	A/northern_shoveler/Missouri/10OS4673/2010	CY133382	93.8	93.8	93.5	93.9	94.1	94.1	94.1	93.5	94.1	93.9	93.1	93.7	93.9	93.9	93.9	93.8	93.5	93.5	93.9	93.9	93.9	93.8	93.4	93.8	94.1	93.5	93.8	93.8	94.2	93.8	93.8	94.1	93.8	93.9	93.8	93.9	93.8	93.9	93.8	94.3	93.5	93.7	93.5	93.5	93.5	93.5	93.5	93.8	93.5						
51	A/Northern_shoveler/Missouri/16OS6248/2016	MG280535	97.8	98.5	97.6	97.4	97.8	97.8	97.8	98.2	97.8	98	97.2	97.6	98.7	98.7	98.7	97.5	97.5	96.3	97.2	97.2	97.2	97.5	96.2	97.9	96.6	98.3	97.9	97.9	97.8	97.9	97.9	96.6	97.9	98.7	97.6	98.7	98.5	98.2	98	97.8	99.1	98.4	96.3	96.3	96.3	96.3	97.4	97.9	98.4	93.4					
52	A/white_winged_scoter/Wisconsin/10OS3922/2010	KC110613	97.2	96.7	97.1	96.8	97.2	97.2	97.2	96.6	97.2	97.2	96.7	96.8	96.8	96.8	96.8	96.7	97	99.2	96.8	96.8	96.8	96.7	99.1	96.6	97	96.4	96.6	96.6	96.7	96.6	96.6	97	96.6	97	96.8	97	97.5	96.7	96.3	96.8	96.7	96.7	100	100	100	100	97.1	97.1	97.1	93.5	96.3				
53	A/Common_Teal/Chany_Lake/29/2019	EPI_ISL_400267	92.7	93.3	92.9	92.6	92.7	92.7	92.7	92.7	92.7	93.3	92.7	92.6	93.4	93.4	93.4	92.6	92.2	93.4	92.9	92.9	92.9	92.6	93.3	93.3	93.1	92.9	93.3	93.3	93.3	93.3	93.3	93.1	93.3	93.3	92.9	93.3	93.3	93	93	92.9	93	93.3	92.9	92.9	92.9	92.9	93	92.6	93	91.2	93.1	92.9			
54	A/garganey/Ukraine/05839-NAMRU3/2006	EPI_ISL_120215	92	92.2	91.8	91.8	92	92	92	91.4	92	92.2	92.2	91.8	92.1	92.1	92.1	92.1	91.4	92.1	92.5	92.5	92.5	92.1	92	92	92.3	92.1	92	92	92.2	92	92	92.3	92	92.2	92.3	92	92.2	92	92	92.2	92	92.2	91.6	91.6	91.6	91.6	91.7	91.6	92	90	92.1	91.6	95.1		
55	A/goose/Karachi/NARC_13N_969/2014	KX602671	92.9	93.1	92.7	93	93.1	93.1	93.1	92.2	93.1	93.3	93.1	93	93.4	93.4	93.4	92.7	92.6	92.7	92.6	92.6	92.6	92.7	92.6	92.6	93.1	93.1	92.6	92.6	92.9	92.6	92.6	93.1	92.6	93.3	92.7	93.3	93.1	93.1	93	93.1	93.3	93.1	92.3	92.3	92.3	92.3	92.5	92.7	92.5	91.7	92.9	92.3	94.6	95.8	
56	A/mallard/Astrakhan/263/1982	CY130095	92.5	93	92.3	92.9	92.7	92.7	92.7	92.2	92.7	93	92.7	92.9	93.1	93.1	93.1	92.6	92.2	92.3	92.5	92.5	92.5	92.6	92.2	92.5	93.4	92.9	92.5	92.5	92.7	92.5	92.5	93.4	92.5	93	92.9	93	93	92.7	92.9	92.7	92.7	93	91.8	91.8	91.8	91.8	92.2	92.3	92.7	90.6	92.9	91.8	96	97.2	96.6	
