# Supplementary material for: Blue-Winged Teals in Guatemala and Their Potential Role in the Ecology of H14 Subtype Influenza a Viruses
Source: Viruses. 2023 Feb 9;15(2):483. doi: 10.3390/v15020483 (PMC9961055; doi:10.3390/v15020483)
Supplement: Supplementary file 1 [file Suppl_Table S8.pdf › Suppl_Table S8.pdf]

Suppl. Table S8. Detailed nucleotide pairwise identity of ORF sequences of NS gene segment of full-length H14 viruses from Guatemala (n=40), North America (n=12), and Eurasia (n=4) during 1982-2019.

# virus	Strain	Reference ID	1	2	3	4	5	6	7	8	9	10-A	10-B	11	12	13	14	15	16	17	18	19	20	21	22	23	24	25	26	27	28
1	A/blue_winged_teal/Guatemala/CIP049H105_15/2011	KJ195680																													
2	A/blue_winged_teal/Guatemala/CIP049H105_31/2011	KY644353	97.7																											
3	A/blue_winged_teal/Guatemala/CIP049H106_62/2011	KJ195672	97.7	99.3																										
4	A/blue_winged_teal/Guatemala/CIP049H108_02/2012	KY644263	97	97.5	97.3																									
5	A/blue_winged_teal/Guatemala/CIP049H108_04/2012	KX960475	96.8	97.4	97.1	99.9																								
6	A/blue_winged_teal/Guatemala/CIP049H108_11/2012	KX960422	97	97.5	97.3	100	99.9																							
7	A/blue_winged_teal/Guatemala/CIP049H109_49/2012	KY644493	96.7	97.3	97	99.7	99.6	99.7																						
8	A/blue_winged_teal/Guatemala/CIP049H109_67/2012	KY673164	99.7	97.7	97.7	97	96.8	97	96.7																					
9	A/blue_winged_teal/Guatemala/CIP049H109_76/2012	KX960382	96.5	98.3	98.1	96.7	96.5	96.7	96.4	96.5																				
10-A (variant #1)	A/blue_winged_teal/Guatemala/CIP049H110_04/2012	KY644216	97	98.7	98.6	96.5	96.4	96.5	96.2	97	97.5																			
10-B (variant #2)	A/blue_winged_teal/Guatemala/CIP049H110_04/2012	KY644244	96.8	97.4	97.1	99.9	99.7	99.9	99.6	96.8	96.5	96.4																		
11	A/blue_winged_teal/Guatemala/CIP049H110_31/2012	KY644222	96.7	98.4	98.3	96.2	96.1	96.2	95.9	96.7	97.3	99.7	96.1																	
12	A/blue_winged_teal/Guatemala/CIP049H113_07/2013	KY644437	96.8	97.4	97.1	99.9	99.7	99.9	99.6	96.8	96.5	96.4	99.7	96.1																
13	A/blue_winged_teal/Guatemala/CIP049H113_08/2013	KY644271	95.9	96.8	96.8	98.4	98.3	98.4	98.1	95.9	95.9	96.2	98.3	95.9	98.3															
14	A/blue_winged_teal/Guatemala/CIP049H113_74/2013	KY673162	95.8	96.7	96.7	98.3	98.1	98.3	98	95.8	95.8	96.1	98.1	95.8	98.1	99.9														
15	A/blue_winged_teal/Guatemala/CIP049H113_76/2013	KY644330	95.9	96.8	96.8	98.4	98.3	98.4	98.1	95.9	95.9	96.2	98.3	95.9	98.3	100	99.9													
16	A/blue_winged_teal/Guatemala/CIP049H116_120/2013	MK327722	96.8	98.6	98.4	97	96.8	97	96.7	96.8	99.4	97.8	96.8	97.5	96.8	96.2	96.1	96.2												
17	A/blue_winged_teal/Guatemala/CIP049H117_13/2013	MK327690	96.7	98.4	98.3	96.8	96.7	96.8	96.5	96.7	99.3	97.7	96.7	97.4	96.7	96.1	95.9	96.1	99.6											
18	A/blue_winged_teal/Guatemala/CIP049H117_34/2013	MK327698	97.1	98.6	98.4	97.3	97.1	97.3	97	97.1	99.4	97.8	97.1	97.5	97.1	96.5	96.4	96.5	99.7	99.6										
19	A/blue_winged_teal/Guatemala/CIP049H117_36/2013	MK327706	97.8	99.6	99.4	97.4	97.3	97.4	97.1	97.8	98.4	98.8	97.3	98.6	97.3	96.7	96.5	96.7	98.7	98.6	98.7									
20	A/blue_winged_teal/Guatemala/CIP049H117_38/2013	MK326660	97.8	99.6	99.4	97.4	97.3	97.4	97.1	97.8	98.4	98.8	97.3	98.6	97.3	96.7	96.5	96.7	98.7	98.6	98.7	100								
21	A/blue_winged_teal/Guatemala/CIP049H117_99/2013	MK327738	97.8	99.6	99.4	97.4	97.3	97.4	97.1	97.8	98.4	98.8	97.3	98.6	97.3	96.7	96.5	96.7	98.7	98.6	98.7	100	100							
22	A/blue_winged_teal/Guatemala/CIP049H117_123/2013	MK327730	96.8	98.6	98.4	97	96.8	97	96.7	96.8	99.4	97.8	96.8	97.5	96.8	96.2	96.1	96.2	100	99.6	99.7	98.7	98.7	98.7						
23	A/blue_winged_teal/Guatemala/CIP049H117_143/2013	MK327762	97.1	98.6	98.4	97.3	97.1	97.3	97	97.1	99.4	97.8	97.1	97.5	97.1	96.5	96.4	96.5	99.7	99.6	100	98.7	98.7	98.7	99.7					
24	A/blue-winged_teal/Guatemala/CIP049H123_13/2014	OP144072	97.7	99.4	99.3	97.3	97.1	97.3	97	97.7	98.3	98.7	97.1	98.4	97.1	96.5	96.4	96.5	98.6	98.4	98.6	99.6	99.6	99.6	98.6	98.6				
25	A/blue-winged_teal/Guatemala/CIP049H123_16/2014	OP144080	97.7	99.4	99.3	97.3	97.1	97.3	97	97.7	98.3	98.7	97.1	98.4	97.1	96.5	96.4	96.5	98.6	98.4	98.6	99.6	99.6	99.6	98.6	98.6	100			
26	A/blue-winged_teal/Guatemala/CIP049H123_33/2014	OP144088	97.7	99.4	99.3	97.3	97.1	97.3	97	97.7	98.3	98.7	97.1	98.4	97.1	96.5	96.4	96.5	98.6	98.4	98.6	99.6	99.6	99.6	98.6	98.6	100	100		
27	A/blue-winged_teal/Guatemala/CIP049H123_57/2014	OP144096	97.7	99.4	99.3	97.3	97.1	97.3	97	97.7	98.3	98.7	97.1	98.4	97.1	96.5	96.4	96.5	98.6	98.4	98.6	99.6	99.6	99.6	98.6	98.6	100	100	100	
28	A/blue-winged_teal/Guatemala/CIP049H123_58/2014	OP144104	97.7	99.4	99.3	97.3	97.1	97.3	97	97.7	98.3	98.7	97.1	98.4	97.1	96.5	96.4	96.5	98.6	98.4	98.6	99.6	99.6	99.6	98.6	98.6	100	100	100	100	
29	A/blue-winged_teal/Guatemala/CIP049H123_59/2014	OP144112	97.5	99.3	99.1	97.1	97	97.1	96.8	97.5	98.1	98.6	97	98.3	97	96.4	96.2	96.4	98.4	98.3	98.4	99.4	99.4	99.4	98.4	98.4	99.9	99.9	99.9	99.9	99.9
30	A/blue-winged_teal/Guatemala/CIP049H123_61/2014	OP144120	97.7	99.4	99.3	97.3	97.1	97.3	97	97.7	98.3	98.7	97.1	98.4	97.1	96.5	96.4	96.5	98.6	98.4	98.6	99.6	99.6	99.6	98.6	98.6	100	100	100	100	100
31	A/blue-winged_teal/Guatemala/CIP049H123_65/2014	OP144129	97.7	99.4	99.3	97.3	97.1	97.3	97	97.7	98.3	98.7	97.1	98.4	97.1	96.5	96.4	96.5	98.6	98.4	98.6	99.6	99.6	99.6	98.6	98.6	100	100	100	100	100
32	A/blue-winged_teal/Guatemala/CIP049H123_75/2014	OP144137	97.5	99.3	99.1	97.1	97	97.1	96.8	97.5	98.1	98.6	97	98.3	97	96.4	96.2	96.4	98.4	98.3	98.4	99.4	99.4	99.4	98.4	98.4	99.9	99.9	99.9	99.9	99.9
33	A/blue-winged_teal/Guatemala/CIP049H123_76/2014	OP144146	97.7	99.4	99.3	97.3	97.1	97.3	97	97.7	98.3	98.7	97.1	98.4	97.1	96.5	96.4	96.5	98.6	98.4	98.6	99.6	99.6	99.6	98.6	98.6	100	100	100	100	100
34	A/blue-winged_teal/Guatemala/CIP049H125_14/2015	OP144154	97.5	99.3	98.8	97.1	97	97.1	96.8	97.5	97.8	98.3	97	98	97	96.4	96.2	96.4	98.1	98	98.1	99.1	99.1	99.1	98.1	98.1	99	99	99	99	99
35	A/blue-winged_teal/Guatemala/CIP049H125_23/2015	OP144162	71.1	71.2	71.3	70.8	70.8	70.8	70.8	70.9	71.1	70.9	70.9	70.9	70.9	70.5	70.5	70.5	71.1	70.9	71.2	71.6	71.6	71.6	71.1	71.2	71.3	71.3	71.3	71.3	71.3
36	A/blue-winged_teal/Guatemala/CIP049H125_59/2015	OP144170	96.7	98.4	98.3	96.8	96.7	96.8	96.5	96.7	99.3	97.7	96.7	97.4	96.7	96.1	95.9	96.1	99.6	99.4	99.6	98.6	98.6	98.6	99.6	99.6	98.4	98.4	98.4	98.4	98.4
37	A/blue-winged_teal/Guatemala/CIP049H125_108/2015	OP144178	96.5	98.3	98.1	96.7	96.5	96.7	96.4	96.5	99.1	97.5	96.5	97.3	96.5	95.9	95.8	95.9	99.4	99.3	99.4	98.4	98.4	98.4	99.4	99.4	98.3	98.3	98.3	98.3	98.3
38	A/blue-winged_teal/Guatemala/CIP049H158_26/2019	OP144186	95.5	97.3	97.1	95.9	95.8	95.9	95.7	95.8	98.4	97.4	95.8	97.1	95.8	95.5	95.4	95.5	98.4	98.3	98.4	97.4	97.4	97.4	98.4	98.4	97.3	97.3	97.3	97.3	97.3
39	A/blue-winged_teal/Guatemala/CIP049H189_08/2019	OP144195	97	98.4	98.3	96.8	96.7	96.8	96.5	97	99.3	97.7	96.7	97.4	96.7	96.1	95.9	96.1	99.6	99.4	99.6	98.6	98.6	98.6	99.6	99.6	98.4	98.4	98.4	98.4	98.4
40	A/blue-winged_teal/Guatemala/CIP049H189_19/2019	OP144203	96.4	98.1	98	97.4	97.3	97.4	97.1	96.4	99	97.4	97.3	97.1	97.3	96.7	96.5	96.7	99.3	99.1	99.3	98.3	98.3	98.3	99.3	99.3	98.1	98.1	98.1	98.1	98.1
41	A/Blue_winged_Teal/Ohio/18OS1695/2018	MN431044	96.4	98	97.8	96.7	96.5	96.7	96.4	96.4	98.8	97.8	96.5	97.5	96.5	95.9	95.8	95.9	99.1	99	99.1	98.1	98.1	98.1	99.1	99.1	98	98	98	98	98
42	A/blue_winged_teal/Texas/UGAI15_6890/2015	KX024587	96.7	98.4	98.3	97.1	97	97.1	96.8	96.7	99.3	97.7	97	97.4	97	96.4	96.2	96.4	99.6	99.4	99.6	98.6	98.6	98.6	99.6	99.6	98.4	98.4	98.4	98.4	98.4
43	A/long_tailed_duck/Wisconsin/10OS3912/2010	KC110600	70.6	70.5	70.6	70	70	70	70	70.8	70.5	70.5	70.2	70.5	70.2	70.3	70.3	70.3	70.6	70.5	70.8	70.9	70.9	70.9	70.6	70.8	70.6	70.6	70.6	70.6	70.6
44	A/long_tailed_duck/Wisconsin/10OS3918/2010	CY133557	70.8	70.6	70.8	70.2	70.2	70.2	70.2	70.9	70.6	70.6	70.3	70.6	70.3	70.5	70.5	70.5	70.8	70.6	70.9	71.1	71.1	71.1	70.8	70.9	70.8	70.8	70.8	70.8	70.8
45	A/long_tailed_duck/Wisconsin/10OS4225/2010	KC110607	91.6	91.2	91.5	91.9	91.8	91.9	91.6	91.6	91.5	90.7	91.8	90.4	91.8	91.2	91	91.2	91.5	91.3	91.8	91.3	91.3	91.3	91.5	91.8	91.5	91.5	91.5	91.5	91.5
46	A/mallard/Wisconsin/10OS3941/2010	CY133270	91.6	91.2	91.5	91.9	91.8	91.9	91.6	91.6	91.5	90.7	91.8	90.4	91.8	91.2	91	91.2	91.5	91.3	91.8	91.3	91.3	91.3	91.5	91.8	91.5	91.5	91.5	91.5	91.5
47	A/northern_shoveler/California/2696/2011	CY146901	71.8	71.6	71.8	71.1	71.1	71.1	71.1	71.6	71.3	71.6	70.9	71.6	71.2	71.3	71.3	71.3	71.5	71.3	71.6	71.8	71.8	71.8	71.5	71.6	71.8	71.8	71.8	71.8	71.8
48	A/Northern_Shoveler/California/D1616217/2016	MK928232	97	99	98.6	97.1	97	97.1	96.8	97	97.5	98	97	98	97	96.1	95.9	96.1	97.8	97.7	97.8	98.8	98.8	98.8	97.8	97.8	98.7	98.7	98.7	98.7	98.7
49	A/northern_shoveler/Mississippi/12OS456/2012	CY167271	71.1	71.2	71.3	70.8	70.8	70.8	70.8	70.9	71.1	71.2	70.9	71.2	70.9	71.1	71.1	71.1	71.1	70.9	71.2	71.6	71.6	71.6	71.1	71.2	71.3	71.3	71.3	71.3	71.3
50	A/northern_shoveler/Missouri/10OS4673/2010	CY133385	70.8	71.2	71.3	70.8	70.8	70.8	70.8	70.6	71.2	71.2	70.9	71.2	70.9	70.8	70.8	70.8	71.3	71.2	71.5	71.6	71.6	71.6	71.3	71.5	71.3	71.3	71.3	71.3	71.3
51	A/Northern_shoveler/Missouri/16OS6248/2016	MG279745	96.4	98.1	98	96.8	96.7	96.8	96.5	96.4	99	97.7	96.7	97.4	96.7	96.1	95.9	96.1	99.3	99.1	99.3	98.3	98.3	98.3	99.3	99.3	98.1	98.1	98.1	98.1	98.1
52	A/white_winged_scoter/Wisconsin/10OS3922/2010	KC110614	70.8	70.6	70.8	70.2	70.2	70.2	70.2	70.9	70.6	70.6	70.3	70.6	70.3	70.5	70.5	70.5	70.8	70.6	70.9	71.1	71.1	71.1	70.8	70.9	70.8	70.8	70.8	70.8	70.8
53	A/Common_Teal/Chany_Lake/29/2019	EPI_ISL_400267	94.1	93.6	93.9	94.4	94.2	94.4	94.1	94.1	93.6	93.3	94.2	93.1	94.2	93.3	93.2	93.3	93.6	93.5	93.9	93.8	93.8	93.8	93.6	93.9	93.9	93.9	93.9	93.9	93.9
54	A/garganey/Ukraine/05839-NAMRU3/2006	EPI_ISL_120215	70.8	71.2	70.8	71.5	71.5	71.5	71.5	70.6	71.2	71.1	71.3	71.1	71.6	71.8	71.8	71.8	71.1	70.9	71.2	71.1	71.1	71.1	71.1	71.2	71.2	71.2	71.2	71.2	71.2
55	A/goose/Karachi/NARC_13N_969/2014	KX602672	94.5	94.2	94.5	94.4	94.2	94.4	94.1	94.5	94.2	93.9	94.2	93.6	94.2	93.3	93.2	93.3	94.2	94.1	94.5	94.4	94.4	94.4	94.2	94.5	94.5	94.5	94.5	94.5	94.5
56	A/mallard/Astrakhan/263/1982	CY005393	95.2	94.5	94.8	94.4	94.2	94.4	94.1	95.5	94.5	93.8	94.2	93.5	94.2	93.3	93.2	93.3	94.5	94.4	94.8	94.6	94.6	94.6	94.5	94.8	94.8	94.8	94.8	94.8	94.8

# virus	29	30	31	32	33	34	35	36	37	38	39	40	41	42	43	44	45	46	47	48	49	50	51	52	53	54	55	56
30	99.9																											
31	99.9	100																										
32	99.7	99.9	99.9																									
33	99.9	100	100	99.9																								
34	98.8	99	99	98.8	99																							
35	71.2	71.3	71.3	71.2	71.3	71.3																						
36	98.3	98.4	98.4	98.3	98.4	98	71.2																					
37	98.1	98.3	98.3	98.1	98.3	97.8	71.1	99.9																				
38	97.1	97.3	97.3	97.1	97.3	97.1	70.8	98.3	98.1																			
39	98.3	98.4	98.4	98.3	98.4	98.3	70.9	99.4	99.3	98.3																		
40	98	98.1	98.1	98	98.1	97.7	70.8	99.1	99	98.3	99.1																	
41	97.8	98	98	97.8	98	97.5	70.8	99	98.8	99	99	99																
42	98.3	98.4	98.4	98.3	98.4	98	71.1	99.4	99.3	98.8	99.4	99.4	99.6															
43	70.5	70.6	70.6	70.5	70.6	70.6	97.5	70.8	70.6	70.6	70.5	70.3	70.5	70.6														
44	70.6	70.8	70.8	70.6	70.8	70.8	97.7	70.9	70.8	70.8	70.6	70.5	70.6	70.8	99.9													
45	91.3	91.5	91.5	91.3	91.5	91.2	70.3	91.3	91.5	90.7	91.6	91.6	90.9	91.3	69.8	69.9												
46	91.3	91.5	91.5	91.3	91.5	91.2	70.3	91.3	91.5	90.7	91.6	91.6	90.9	91.3	69.8	69.9	100											
47	71.6	71.8	71.8	71.6	71.8	71.8	96.1	71.6	71.5	71.2	71.3	71.2	71.2	71.5	96.2	96.4	70.6	70.6										
48	98.6	98.7	98.7	98.6	98.7	99.1	71.3	97.7	97.5	96.5	97.7	97.4	97.3	97.7	70.6	70.8	90.7	90.7	71.8									
49	71.2	71.3	71.3	71.2	71.3	71.3	98.1	71.2	71.1	70.8	70.9	70.8	70.8	71.1	97.4	97.5	69.6	69.6	96.5	71.3								
50	71.2	71.3	71.3	71.2	71.3	71.3	97.7	71.5	71.3	71.1	71.2	71.1	71.1	71.3	97.5	97.7	70	70	96.4	71.3	98.1							
51	98	98.1	98.1	98	98.1	97.7	71.1	99.1	99	98.6	99.1	99.1	99.3	99.4	70.6	70.8	91	91	71.5	97.4	71.1	71.3						
52	70.6	70.8	70.8	70.6	70.8	70.8	97.7	70.9	70.8	70.8	70.6	70.5	70.6	70.8	99.9	100	69.9	69.9	96.4	70.8	97.5	97.7	70.8					
53	93.8	93.9	93.9	93.8	93.9	93.6	70.9	93.5	93.3	92.9	93.8	93.5	93.3	93.5	70	70.2	94.6	94.6	70.9	93.2	70.5	70	93.2	70.2				
54	71.3	71.2	71.2	71.1	71.2	71.3	91.2	71.2	71.1	71.2	71.1	70.8	71.1	71.2	90.7	90.9	70	70	90.9	71.3	91.6	91	71.1	90.9	71.2			
55	94.4	94.5	94.5	94.4	94.5	94.2	70.8	94.1	93.9	93.5	94.4	94.1	93.9	94.1	69.9	70	94.9	94.9	70.8	93.8	70.3	70.5	93.8	70	98.8	70.9		
56	94.6	94.8	94.8	94.6	94.8	94.5	70.9	94.4	94.2	94.1	94.6	94.1	93.9	94.4	70.2	70.3	94.1	94.1	71.6	94.1	70.3	70.5	94.1	70.3	96.4	70.9	96.7	
